# Shortest-Path Network Analysis Is a Useful Approach toward Identifying Genetic Determinants of Longevity

**DOI:** 10.1371/journal.pone.0003802

**Published:** 2008-11-25

**Authors:** J. R. Managbanag, Tarynn M. Witten, Danail Bonchev, Lindsay A. Fox, Mitsuhiro Tsuchiya, Brian K. Kennedy, Matt Kaeberlein

**Affiliations:** 1 Center for the Study of Biological Complexity, Virginia Commonwealth University, Richmond, Virginia, United States of America; 2 Department of Mathematics and Applied Mathematics, Virginia Commonwealth University, Richmond, Virginia, United States of America; 3 Department of Biochemistry, University of Washington, Seattle, Washington, United States of America; 4 Department of Pathology, University of Washington, Seattle, Washington, United States of America; Centre for Genomic Regulation, Spain

## Abstract

**Background:**

Identification of genes that modulate longevity is a major focus of aging-related research and an area of intense public interest. In addition to facilitating an improved understanding of the basic mechanisms of aging, such genes represent potential targets for therapeutic intervention in multiple age-associated diseases, including cancer, heart disease, diabetes, and neurodegenerative disorders. To date, however, targeted efforts at identifying longevity-associated genes have been limited by a lack of predictive power, and useful algorithms for candidate gene-identification have also been lacking.

**Methodology/Principal Findings:**

We have utilized a shortest-path network analysis to identify novel genes that modulate longevity in *Saccharomyces cerevisiae*. Based on a set of previously reported genes associated with increased life span, we applied a shortest-path network algorithm to a pre-existing protein–protein interaction dataset in order to construct a shortest-path longevity network. To validate this network, the replicative aging potential of 88 single-gene deletion strains corresponding to predicted components of the shortest-path longevity network was determined. Here we report that the single-gene deletion strains identified by our shortest-path longevity analysis are significantly enriched for mutations conferring either increased or decreased replicative life span, relative to a randomly selected set of 564 single-gene deletion strains or to the current data set available for the entire haploid deletion collection. Further, we report the identification of previously unknown longevity genes, several of which function in a conserved longevity pathway believed to mediate life span extension in response to dietary restriction.

**Conclusions/Significance:**

This work demonstrates that shortest-path network analysis is a useful approach toward identifying genetic determinants of longevity and represents the first application of network analysis of aging to be extensively validated in a biological system. The novel longevity genes identified in this study are likely to yield further insight into the molecular mechanisms of aging and age-associated disease.

## Introduction

Network-based approaches are one particularly useful method for representing complex biological systems [1]. As the technical acumen for characterizing complex interactions has advanced, so has the feasibility of building large scale networks via sophisticated computational tools that facilitate elucidation of such systems [2]. Network analysis methods have provided insights into many types of biological interactions, including transcriptional regulation, genetic interaction, protein–protein interactions, expression correlation, sequence homology and redundant patterns of associations called motifs [3], [4], [5]. Network theory has been employed in the discovery of hereditary disease-genes in people, thanks in part to the availability of human genome-wide protein–protein interaction (PPI) data sets and powerful computational tools that are able to assess the complex topology of PPI networks [6].

Originally proposed by Witten [7], [8], network methods are now being applied to unravel the complexity of aging. For example, Xue et al. have characterized protein–protein interaction network modules associated with aging in humans and flies and performed limited biological validation of their network in *Caenorhabditis elegans* [9]. In another study, several potential longevity-associated genes (LAGs) were predicted in *C. elegans*, based on interactions with previously described LAGs [10]. Thus far, however, a comprehensive analysis of an entire putative longevity network has not been described and the utility of network approaches to predict novel longevity genes and pathways remains to be determined.

The budding yeast *Saccharomyces cerevisiae* has served as a particularly useful model system for identifying genetic and environmental factors that modulate cellular longevity [11], [12]. Two distinct types of aging have been described in yeast: replicative aging, which refers to the number of cell divisions a mother cell is capable of completing, and chronological aging, which is defined by the length of time a cell can survive in a non-dividing state. Several known yeast LAGs have counterparts that influence aging in multicellular eukaryotes [13], providing confidence that at least some mechanisms of yeast aging are likely to be evolutionarily conserved.

## Results

**Figure 1:**
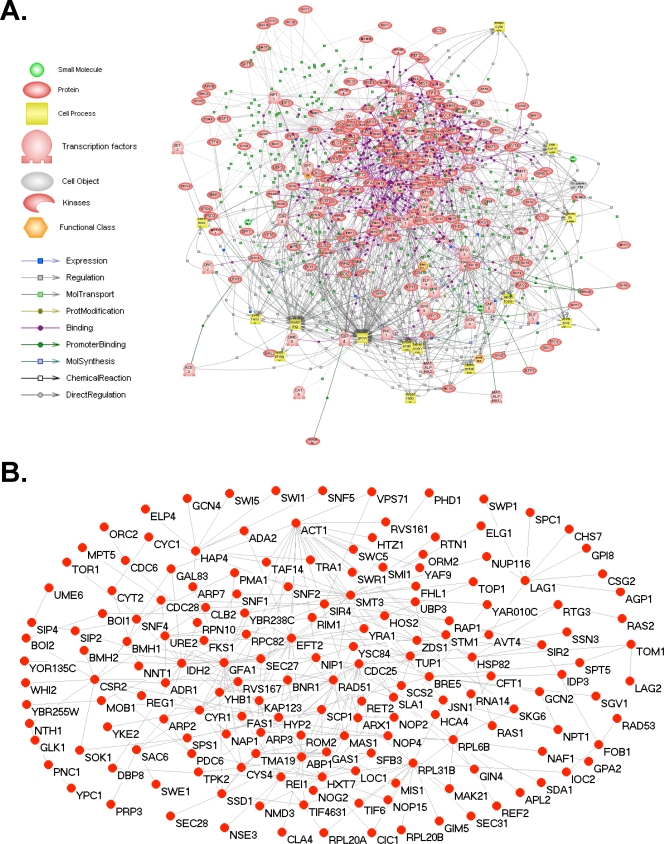
A shortest-path longevity network in yeast. (A) A Composite shortest path longevity network was constructed using the set of yeast longevity associated genes listed in Table S1. (B) The Binding shortest-path longevity network was extracted from the Composite shortest-path longevity network by only considering protein–protein interactions.

We have applied a shortest-path network strategy (see **Materials and Methods**) in order to identify previously unknown LAGs that modulate yeast replicative life span (RLS). This approach is based on the conjecture that proteins connecting pairs of other proteins with well-defined biological functions (e.g., modulating longevity) have a higher probability to share that function, as compared to those selected at random [10]. In order to construct the shortest path longevity network (SPLN), we first generated a list of 40 previously reported LAGs, with the criteria that only genes associated with *increased* RLS were included in the set (**Table S1**). Using this set of RLS LAGs and a dataset of known interactions (Pathway Studio 5.0, Ariadne Genomics, Rockville, MD), we first generated a Composite SPLN defined by the least number of interactions required to link each pair-wise combination of input LAGs (**Figure 1a**). The Composite SPLN (**Table S2**) was comprised of 220 genes/proteins associated by several interaction types that included physical binding, genetic relationships, and transcriptional regulation, among others. We then extracted the binding (protein–protein) interactions from the Composite SPLN to build a Binding SPLN for RLS analysis (**Table S3**). The resulting Binding SPLN contained 171 genes/proteins, including 33 of the 40 input LAGs (7 of the input LAGs are not integrated in the Binding SPLN but are components of the Composite SPLN) and 138 putative novel longevity genes (**Figure 1b**).

**Figure 2:**
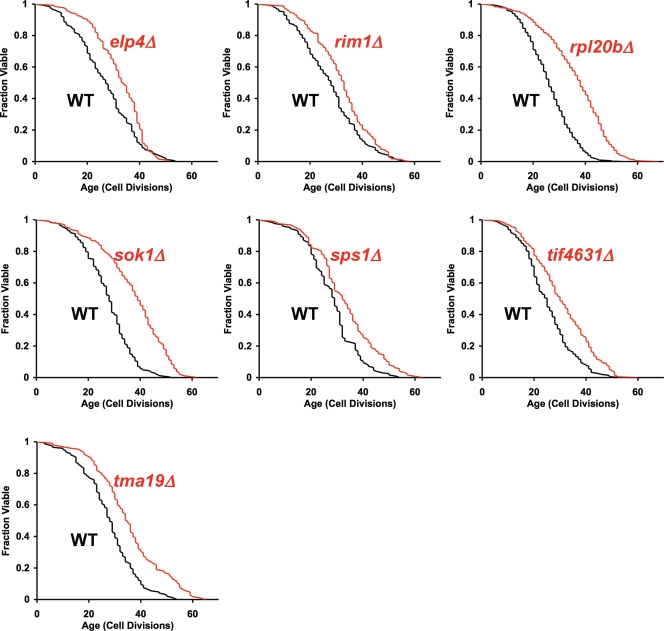
Gene deletions that are significantly long-lived in both haploid mating types predicted from the binding shortest path longevity network. Replicative life span is plotted for *elp4Δ*, *rim1Δ*, *rpl20bΔ*, *sok1Δ*, *sps1Δ*, *tif4631Δ*, and *tma19Δ* relative to experiment matched wild type (WT) cells. Replicative life span extension was significant in both mating types (p<0.05, Wilcoxon Rank-Sum Test). Pooled data from both mating types is shown. Mean life spans, numbers of mother cells analyzed, and p-values are provided in Table S4.

**Figure 3:**
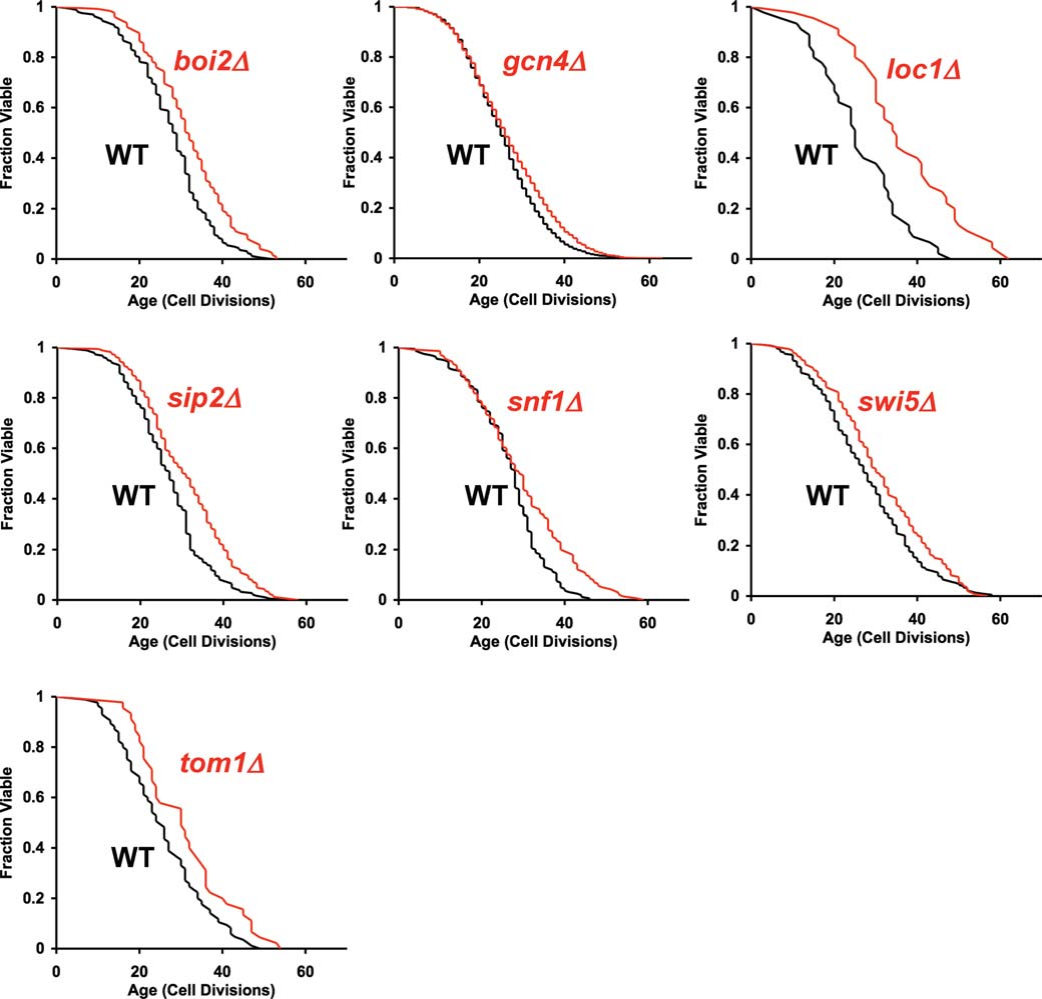
Gene deletions that are significantly long-lived when data is pooled from both haploid mating types predicted from the binding shortest path longevity network. Replicative life span data is plotted for *boi2Δ*, *gcn4Δ*, *loc1Δ*, *sip2Δ*, *snf1Δ*, *swi5Δ*, and *tom1Δ*, relative to experiment matched wild type cells. Replicative life span extension was significant in data pooled from both mating types (p<0.05, Wilcoxon Rank-Sum Test). Pooled data from both mating types is shown. Mean life spans, numbers of mother cells analyzed, and p-values are provided in Table S4.

In order to determine whether our Binding SPLN successfully predicted new LAGs, we performed RLS analysis on 88 single-gene deletion strains corresponding to each of the genes/proteins contained in the Binding SPLN for which a deletion strain was available in the yeast ORF deletion collection (**Table S4**). We utilized a previously described iterative approach for large-scale RLS analysis [14]: each deletion mutant was initially assayed in strains derived from the *MATα* ORF deletion collection and, in cases where a statistically significant (p<0.05) increase in median RLS was observed, RLS was determined for independently derived isogenic cells obtained from the *MAT* **a** ORF deletion collection [14]. This analysis led to the identification of seven single-gene deletion strains (*elp4Δ*, *rim1Δ*, *rpl20bΔ*, *sok1Δ*, *sps1Δ*, *tif4631Δ*, and *tma19Δ*) that were significantly long-lived in both haploid mating types (**Figure 2**) and seven additional single-gene deletions (*boi2Δ*, *gcn4Δ*, *loc1Δ*, *sip2Δ*, *snf1Δ*, *swi5Δ*, and *tom1Δ*) that were significantly long-lived if data from both mating types were pooled, but which had not reached statistical significance in the *MAT* **a** mating type at the time this manuscript was being prepared (**Figure 3**). Nine additional strains (*abp1Δ*, *clb2Δ*, *idp3Δ*, *phd1Δ*, *rtn1Δ*, *rvs161Δ*, *sec28Δ*, *taf14Δ*, and *ysc84Δ*) showed a significant increase in RLS in the *MATα* background, but were not long-lived in the *MAT* **a** background. We have previously observed divergent phenotypes in *MATα* and *MAT* **a** strains lacking the same gene taken from the ORF deletion collection [13], [15]. While mating-specific effects on longevity cannot be excluded a priori, in most cases these differences are likely due to additional genetic changes in one or the other mating type that arose during creation or propagation of the strain sets. RLS data for each of the 88 deletion strains analyzed are provided in **Table S4**.

**Figure 4:**
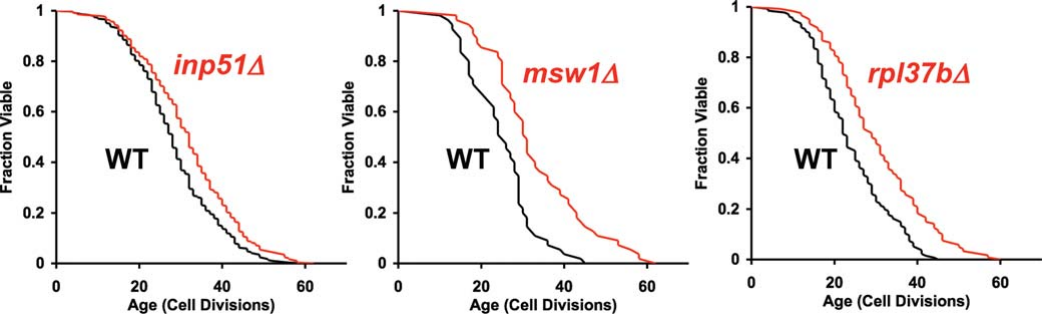
Novel longevity associated genes identified from replicative life span analysis of 564 randomly selected single-gene deletion strains. Replicative life span is plotted for *inp51Δ*, *msw1Δ*, and *rpl37bΔ* relative to experiment matched wild type (WT) cells. Replicative life span extension was significant in both mating types (p<0.05, Wilcoxon Rank-Sum Test). Pooled data from both mating types is shown. Mean life spans, numbers of mother cells analyzed, and p-values are provided in Table S5.

Based on comparable RLS analysis of 564 randomly chosen single-gene deletion strains, we can estimate the expected frequency of life span extension in strains carrying deletion alleles of non-essential genes across the entire yeast genome (RLS data for 564 randomly selected deletion strains provided **Table S5**). Among the 564 randomly selected deletion strains, 2.7% (15/564) were significantly long-lived in both mating types, while 4.3% (24/564) were long-lived if data were pooled for both mating types (**Table 1**). A majority of these long-lived deletion mutants contained in the set of 564 randomly selected deletion strains have been previously described [15]; however, here we report three novel long-lived strains (*inp51Δ*, *msw1Δ*, and *rpl37bΔ*) that show increased RLS in both mating types (**Figure 4**). Inp51 codes for a phosphatidylinositol 4,5-bisphosphate 5-phosphatase, Msw1 codes for a mitochondrial tryptophanyl-tRNA synthetase, and Rpl37b codes for a protein component of the large ribosomal subunit. The mechanism(s) by which these proteins modulate longevity has not been further characterized; however, abundance of the ribosomal large subunit has recently emerged as a key longevity determinant in yeast [16] and it seems likely that deletion of *RPL37B* is increasing life span via a similar mechanism.

**Table 1:** Increased life span in single-gene deletion strains from the binding shortest-path longevity network relative to randomly selected strains.

Dataset	Both Haploid Mating Types	*p* - value	Pooled<0.05	*p* - value
Binding SPLN (88)	8.0 % (7)	-	15.9% (14)	-
R564 (564)	2.7 % (15)	0.02	4.3% (24)	1.6×10^−4^
DELSET (4681)	1.5% (72)	8.2×10^−4^	6.1% (288)	1.1×10^−3^

The percentage of single-gene deletion strains that are significantly long-lived in the Binding shortest-path longevity network ( Binding SPLN), a randomly selected set of 564 deletion strains (R564), or the 4681 deletion strains from the deletion collection for which RLS data has been obtained (DELSET) is shown. The number of strains in each category is shown in parentheses. The *p*-value category refers to the results of a Fisher's Exact test comparing the frequency of increased RLS in each random set (R564 or DELSET) relative to the Binding SPLN.

If the Binding SPLN algorithm successfully predicted novel LAGs, then we expect that deletion of Binding SPLN components will be more likely to be associated with altered RLS compared to randomly selected gene deletions. Consistent with this prediction, increased RLS is significantly enriched among the 88 deletion strains from the Binding SPLN relative to the randomly selected set of 564 deletion strains using the highly stringent criterion of increased RLS in both mating types (*p* = 0.02, Fisher's exact test). A significant enrichment is also observed using the less stringent criterion of increased RLS in data pooled between mating types (*p* = 1.6×10^−4^, Fisher's exact test). Among the Binding SPLN deletion strains, 8% (7/88) are significantly long-lived in both mating types and 15.9% (14/88) are significantly long-lived when data from both mating types are pooled, compared to only 2.7% (15/564) and 4.3% (24) in each category for the randomly selected set of 564 deletion strains (Table 1). The set of 88 deletion strains corresponding to the Binding SPLN is also significantly enriched for increased RLS when compared against data obtained as part of an ongoing genome-wide analysis of RLS across the approximately 4700 single-gene deletion strains contained in the haploid yeast ORF deletion collection [14] (**Table 1**), in which only 1.5% (72/4681) currently show a significant increase in life span in both mating types and 6.1% (288/4681) are significantly long-lived when data is pooled across haploid mating types. These data demonstrate that the Binding SPLN is significantly enriched for genes that limit RLS, suggesting that the SPLN successfully predicts LAGs.

As a further test of the predictive power of the SPLN, we next asked whether the 88 Binding SPLN-associated gene deletions were also enriched for decreased RLS. Since the Binding SPLN was built based on PPIs, which include both positive and negative regulatory interactions, many of the Binding SPLN components are likely to function in a longevity-promoting role, and deletion of these components may be associated with shortened life span. To determine whether this was the case, we used *MATα* mean RLS cutoff values of 20 generations and 15 generations (the average RLS of the parental strain is ∼26 generations) and asked whether short-lived gene deletions occur more frequently in the Binding SPLN, relative to either the set of 564 randomly chosen deletion strains or the current data available for the entire deletion collection. These cutoff values were arbitrarily chosen; however, computational analysis of greater than 5000 individual mother cell life spans indicates that a wild type life span distribution will yield a 5-cell mean RLS of 15 or lower less than 0.3% of the time and 20 or lower approximately 7% of the time (**Figure S1**). Thus, deletion mutants with a mean RLS of less than 20 generations are likely to be short-lived and those with mean RLS less than 15 are almost certainly short-lived, relative to the parental strain. Among the Binding SPLN deletion strains, 36% (32/88) had a mean RLS less than 20 generations, while only 17% of either randomly selected deletions (96/564) or the entire deletion collection (818/4681) had a mean RLS less than 20 generations (**Table 2**). At a mean RLS cutoff of 15 generations, enrichment for short RLS in the Binding SPLN is even more pronounced: 15% (13/88) of Binding SPLN deletion strains, 3% (19/564) of the randomly selected deletions, and 5% (231/4681) of the entire deletion collection had mean RLSs below this value. In every case, the Binding SPLN was significantly enriched (p<0.05, Fisher's exact test) for short-lived deletion mutants relative to either the set of 564 randomly selected deletions or the entire deletion collection.

**Table 2:** Decreased life span in single-gene deletion strains from the binding shortest-path longevity network relative to randomly selected strains.

Dataset	Mean RLS<15	*p* - value	Mean RLS<20	*p* - value
Binding SPLN (88)	14.8 % (13)	-	36.4% (32)	-
R564 (564)	3.4 % (19)	8.2×10^−5^	17.0% (96)	5.5×10^−5^
DELSET (4681)	5% (231)	4.6×10^−4^	17.5% (818)	2.3×10^−5^

The percentage of single-gene deletion strains with an observed mean replicative life span (MRLS) less than 20 or 15 in the Binding shortest-path longevity network (SPLN), a randomly selected set of 564 deletion strains (R564), or the 4681 deletion strains from the deletion collection for which RLS data has been obtained (DELSET) is shown. The number of strains in each category is shown in parentheses. The *p*-value category refers to the results of a Fisher's Exact test comparing the frequency of short RLS in each random set (R564 or DELSET) relative to the Binding SPLN.

We considered the possibility that genes contained in both the Binding SPLN and the randomly selected set of 564 deletion strains might complicate our quantification of enrichment for LAGs in the Binding SPLN. A total of eleven genes were present in both the predicted LAGs from the Binding SPLN and the random set of 564 deletion strains (**Table S6**). If these genes are excluded from the analysis, the Binding SPLN is still significantly enriched for both long-lived and short-lived (*p*<0.05, Fisher's exact test in every case) deletion mutants relative to the randomly selected set (**Table S6**). Therefore, we conclude that the set of predicted LAGs in the Binding SPLN is enriched for both longevity-promoting and longevity-limiting genes/proteins, and that our Binding SPLN analysis successfully predicts LAGs at a rate significantly greater than can be achieved by randomly selecting genes/proteins from the genome.

## Discussion

We are encouraged by the success of our initial SPLN analysis at predicting novel LAGs; however, we also recognize that some features of our experimental design may have limited the predictive power of the SPLN. For example, the set of previously reported LAGs used to derive the SPLN was generated based on RLS data from studies performed by multiple laboratories using a variety of yeast isolates of diverse genetic composition (**Table S1**). This approach was taken in order to be as inclusive as possible when building the initial SPLN; however, strain-specific effects are known to influence RLS, and some of the mutations reported to increase RLS in other strain backgrounds do not have a similar life span-extending effect in the parental strains of the ORF deletion collection used for all RLS analysis in this study [17]. Indeed, among the 40 previously reported LAGs used to build the SPLN (**Table S1**), less than half are significantly long-lived as deletion alleles in the parental strains used for this study. Future iterations of the SPLN are therefore likely to benefit by limiting the input genes/proteins to only those known to influence aging in BY4741 and BY4742.

A second feature that may have limited the predictive power of the Binding SPLN is the sole use of PPIs in order to build the network. Reported protein–protein interactions from large-scale data sets may have a significant false-positive rate, which can result in sub-networks of the Binding SPLN that do not represent true *in vivo* interactions. In addition, PPIs represent only a fraction of all possible interactions that can occur in cells. For example, transcriptional regulation (or repression) of a gene by a transcription factor would not be represented in the Binding SPLN, yet may be highly relevant in determining longevity. Thus, a comprehensive analysis of the Composite SPLN is likely to identify additional RLS LAGs that were not identified here. This study has yet to be performed; however, based on our analysis to date, the Composite SPLN contains at least one additional long-lived deletion strain that is not present in the Binding SPLN, *ypt6Δ* (**Figure S2**), and is already enriched for both long- and short-lived mutants relative to the rest of the deletion collection (**Table S7**). *YPT6* codes for a RAS-like GTPase required for fusion of endosome-derived vesicles with the late Golgi and is homologous to the *C. elegans* LAG *rab-10* [13], which is thought to act in the same pathway as dietary restriction (DR) and has recently been shown to modulate resistance to polyglutamine toxicity in a nematode model of Huntington's disease [18], [19]. Following comprehensive analysis of the Composite SPLN, it will be of interest to determine whether the Composite SPLN is more or less efficient at predicting LAGs compared to the Binding SPLN.

An alternative approach to the Binding SPLN algorithm used in this study would be to focus solely on genes coding for proteins that interact directly with known LAGs. If only direct PPIs involving the 40 previously reported LAGs contained in **Table S1** are considered, a “first neighbors” longevity network containing 1375 genes/proteins is obtained (**Table S8**). Although complete biological validation of this first neighbors longevity network is not currently feasible, an estimate of the predictive power of such a strategy can be obtained by considering the 937 gene deletions from this network for which some limited RLS data has already been obtained as part of our ongoing genome-wide study. Relative to the entire deletion collection, the first neighbors longevity network is significantly enriched for short-lived deletions (**Table S8**). There is a trend toward enrichment for long-lived deletions, but this trend has not reached statistical significance. In each case, the SPLN was a better predictor of altered life span than the first neighbors longevity network. Thus, we conclude that by constraining our network to shortest-path interactions, the network size is reduced by approximately 10-fold and true-positive interactions are likely to be enriched.

Despite the limitations of this first iteration of the SPLN, the algorithm successfully predicted novel LAGs at a rate significantly greater than expected by random chance. In addition, several features of the network are notable. For example, *SOK1*, deletion of which was observed to increase RLS in both haploid mating types, interacts genetically with both the target of rapamycin (TOR) kinase and the cyclic AMP-dependent protein kinase (PKA); overexpression of *SOK1* suppresses phenotypes associated with reduced PKA activity and confers resistance to the TOR-inhibitor rapamycin [20], [21]. Our data demonstrate that deletion of *SOK1* is sufficient to confer increased RLS (**Figure 2**). Taken together, these results support the hypothesis that Sok1 functions in parallel with TOR and PKA to modulate longevity. This is of particular interest, as both TOR and PKA, along with the ribosomal S6 kinase ortholog Sch9, are thought to mediate life span extension in response to DR, and epistasis analysis places deletion of *TOR1*, deletion of *SCH9*, and mutations that reduce PKA activity in the same pathway as DR and in a pathway parallel to the histone deacetylase Sir2 [15], [22], [23], [24], [25]. We therefore propose that deletion of *SOK1* is a novel genetic mimic of DR in yeast.

In addition to *SOK1*, at least three other gene deletions found to increase life span in both haploid mating types are likely to function in the same longevity pathway downstream of TOR, PKA, and Sch9 by regulating mRNA translation: *RPL20B*, which codes for a constituent of the large ribosomal subunit, *TIF4631*, which codes for a translation initiation factor, and *TMA19*, which codes for a homolog of translationally controlled tumor protein and physically associates with ribosomes [26]. Tif4631 was recently identified as one member of a set of evolutionarily conserved longevity factors that modulate aging by regulating mRNA translation [13]. In a separate study, we have recently reported that Rpl20b also functions downstream of TOR and Sch9 and modulates replicative life span by a mechanism that involves increased translation of Gcn4 [16]. Both Gcn4 and the eIF2 kinase Gcn2, which regulates translation of Gcn4, were identified by our SPLN analysis, as was *LOC1*, which is constituent of 66S pre-ribosomal particles and is required for normal abundance of ribosomal 60S subunits [16].

The fact that the Binding SPLN identified new components of the nutrient responsive TOR longevity pathway is an encouraging indication that the structure of the Binding SPLN accurately reflects the most important aspects of longevity control in yeast. It seems unlikely, however, that the LAGs contained in the SPLN are confined to TOR-related functions, as several of the previously reported LAGs used to generated the SPLN are thought to function in pathways parallel to TOR signaling, and many of the novel LAGs identified from this study have no known link to TOR signaling. For example, Boi2 is a fungal-specific protein involved in polar growth and bud emergence. We speculate Boi2 influences RLS, which is a measure of the capacity to form buds, via it's role in bud emergence, perhaps by modulating retention of extrachromosomal rDNA circles or oxidatively damaged proteins in the mother cell, both of which are associated with yeast aging [27], [28]. Other examples of novel LAGs identified from the Binding SPLN that are likely to be unrelated to TOR activity include Elp4, Sps1 and Rim1. Epl4 functions as an elongator complex subunit required for modification of wobble nucleosides in tRNA; Sps1 is required for localization of enzymes involved in spore wall synthesis; and Rim1 is a single-stranded DNA-binding protein essential for mitochondrial genome maintenance. Thus, the SPLN algorithm successfully identified novel LAGs of diverse function, allowing for future determination of the varied genetic relationships and molecular mechanism(s) by which replicative aging is modulated in yeast.

The ability to accurately predict genetic and environmental determinants of longevity has previously proven difficult. Several approaches toward longevity gene identification have been attempted, including characterization of gene expression and physiological biomarkers of longevity, candidate gene analyses, and unbiased genomic screening. The data presented here demonstrate that SPLN analysis is a useful tool for predicting genes/proteins that modulate longevity. Although the analysis described here was limited to genes/proteins that modulate yeast RLS, similar approaches are equally applicable to other organismal systems for which large-scale interaction data sets are available, including worms, flies, and mice. To the best of our knowledge, this is the first example of a quantitatively predictive algorithm for identifying novel LAGs and, as such, represents an important advance in aging-related science. As more sophisticated data sets become widely available, the predictive ability of our SPLN approach is likely to improve substantially, and may prove particularly useful for identifying candidate longevity loci in humans, where life span analysis is not a feasible approach.

## Materials and Methods

### Construction of composite and binding shortest path longevity networks

A set of genes that have been reported to be associated with increased replicative life span (RLS) in *Saccharomyces cerevisiae* was generated. This set was derived from our Aging Genes/Interventions Database (formerly the SAGE KE Genes/Interventions Database [29]; http://www.kaeberleinlab.org/ageid), the GenAge database [30], and from independent literature searches. Based on this set of longevity associated genes (LAGs), a Composite shortest-path longevity network (SPLN) was constructed by introducing names of genes (canonical names) from our compiled set into PATHWAY STUDIO 5 software (Ariadne Genomics, Rockville, MD) and selecting the shortest path mode for network building [10]. The software identified associations among the input genes by referencing a proprietary yeast interaction database. The composite SPLN integrated many classes of gene/protein interactions such as binding interactions, genetic interactions, transcriptional regulatory interactions, post-translational chemical interactions, and others. The Composite SPLN yielded many predicted LAGs that were located along the paths of the input genes (**Figure S1**). Next, we extracted all the binding interactions from the Composite SPLN and used those to construct a Binding SPLN that consisted of easily testable vertices (i.e. canonical genes as opposed to mechanical processes, complexes, etc). The Binding SPLN was comprised of 171 genes/proteins, including the 33 of the 40 input LAGs (7 of the input LAGs did not have any binding interactions in the binding SPLN but had other interactions in the composite SPLN), 45 essential genes, and 5 genes that either had no known deletions or had representative strains that were not viable. The remaining 88 genes from the Binding SPLN were examined by RLS analysis as single-gene deletions.

### Replicative life span analysis

Replicative life span was assayed as described [17], [31]. The deletion strains employed in the replicative aging experiments were derivatives of BY4742 (MATα his3Δ1 leu2Δ0 lys2Δ0 ura3Δ0) and BY4741 (MATa his3Δ1 leu2Δ0 met15Δ0 ura3Δ0). The parental wild-type strains (BY4741, BY4742) and the yeast ORF deletion collection [32] were obtained from Research Genetics (Carlsbad, CA). The deletion strains chosen for life-span experiments were thawed from frozen stock (−80°C, 25% glycerol) and plated onto rich media (YPD). After 48-hour incubation in 30°C, selected colonies were patched to YPD. The following evening, the cells were lightly patched onto fresh YPD plates for RLS determination. After an overnight incubation at 30°C, the yeast cells were arrayed on YPD plate with a micromanipulator and left to undergo 1–2 divisions. RLS was measured for individually selected virgin cells. Plates that were not used during the experiments were wrapped with parafilm to preclude desiccation. Life span plates were stored at 4–6°C overnight. Daughter cells were separated from mother cells by gentle manipulation with fine dissecting needle and tabulated every 2–4 hours. All experiments were performed ‘blind’, such that the strains were coded in a manner precluding identification of any strain by individuals performing microdissection during the course of the experiment.

The iterative process for large-scale RLS determination was previously described [15]. Based on a numerical analysis described in the prior report [15], it was established that a majority of long-lived strains can be accurately classified based on initial determination of RLS for 5 mother cells from a *MATα* single-gene deletion strain, followed by further analysis of additional mother cells from those *MATα* deletion strains that have a 5-cell mean RLS greater than 26 generations. Those strains that achieve a p-value of 0.05 or less as *MATα* deletions are verified by examining the RLS of the corresponding deletion independently derived in the *MAT* **a** mating type. This iterative approach was used to identify long-lived single-gene deletion strains from 564 randomly selected mutants [15] and is currently in use to characterize the aging potential of 4775 single-gene deletions strains in the yeast ORF deletion collection, of which RLS data has been obtained for 4680 (at least 5 mother cells examined).

### Statistical Analysis

P-values for replicative life span analysis were calculated using a 2-tailed Wilcoxon Rank-Sum test. In each case, mother cell replicative life spans for the deletion mutant under examination were compared to mother cell replicative life spans for experiment matched wild type (BY4742 for *MATα* cells, BY4741 for *MAT* **a)** mother cells. The MATLAB 7.2 ‘ranksum’ function was used to calculate all Wilcoxon test p-values. To determine whether Binding SPLN or the Composite SPLN was enriched for short- or long-lived deletion strains relative to the randomly selected set of 564 deletion mutants [14] or the current data set for the entire deletion collection, a Fisher's exact test was performed using the Fisher's exact test calculator at http://www.langsrud.com/fisher.htm.

## Supporting Information

Figure S1 Distribution of mean replicative life spans for 5 and 10 cell sets of BY4742 mother cells.
(0.13 MB PDF)

Figure S2 Deletion of YPT6, a component of the composite shortest-path longevity network, increases replicative life span.
(0.11 MB PDF)

Table S1 Set of yeast genes reported to be associated with increased replicative life span used to construct the shortest-path longevity network.
(0.11 MB PDF)

Table S2 Relation table for the composite shortest path longevity network.
(0.16 MB PDF)

Table S3 Components of the Binding shortest-path longevity network.
(0.10 MB PDF)

Table S4 Replicative life span analysis of single-gene deletion strains corresponding to genes in the binding shortest-path longevity network.
(0.14 MB PDF)

Table S5 Replicative life span analysis of 564 randomly selected single-gene deletion strains.
(0.36 MB PDF)

Table S6 Genes contained in both the randomly selected set of 564 single-gene deletion strains and the predicted longevity associated genes in the binding shortest path longevity network.
(0.12 MB PDF)

Table S7 Enrichment of longevity-associated genes in the Composite shortest-path longevity network.
(0.08 MB PDF)

Table S8 The first neighbors longevity network has less predictive power than the Binding SPLN.
(0.17 MB PDF)
