# Supplementary material for: Shortest-Path Network Analysis Is a Useful Approach toward Identifying Genetic Determinants of Longevity
Source: PLoS One. 2008 Nov 25;3(11):e3802. doi: 10.1371/journal.pone.0003802 (PMC2583956; doi:10.1371/journal.pone.0003802)
Supplement: Figure S1 — Distribution of mean replicative life spans for 5 and 10 cell sets of BY4742 mother cells. (0.13 MB PDF) [file pone.0003802.s001.pdf]

Figure S1. Distribution of mean replicative life spans for 5 and 10 cell sets of BY4742 mother cells. Replicative life span (RLS) was determined for 5058 individual mother cells from the BY4742 strain background. These data were derived from more than 100 independent RLS experiments. (**A**) Survival plot of 5058 BY4742 mother cells. (**B**) Frequency at which the mean RLS of 5-cell sets randomly selected from the 5058 mother cell RLS values falls within 5-generation intervals. (**C**) Frequency at which the mean RLS of 10-cell sets randomly selected from the 5058 mother cell RLS values falls within 5-generation intervals. Each row represents the results of an independent computational simulation in which 50,000 5 or 10 cell sets were randomly chosen from the 5058 BY4742 RLS values and mean RLS of each randomly selected set was calculated. The values shown are the frequency that the randomly selected sets had a mean RLS within the given interval within each simulation. These values can be used to predict the probability that a given mutant has a life span distribution similar to that of BY4742. For example, if a 5-cell set from a particular deletion mutant is 15, then the probability of that mutant having a wild type life span is approximately 0.0025.

A

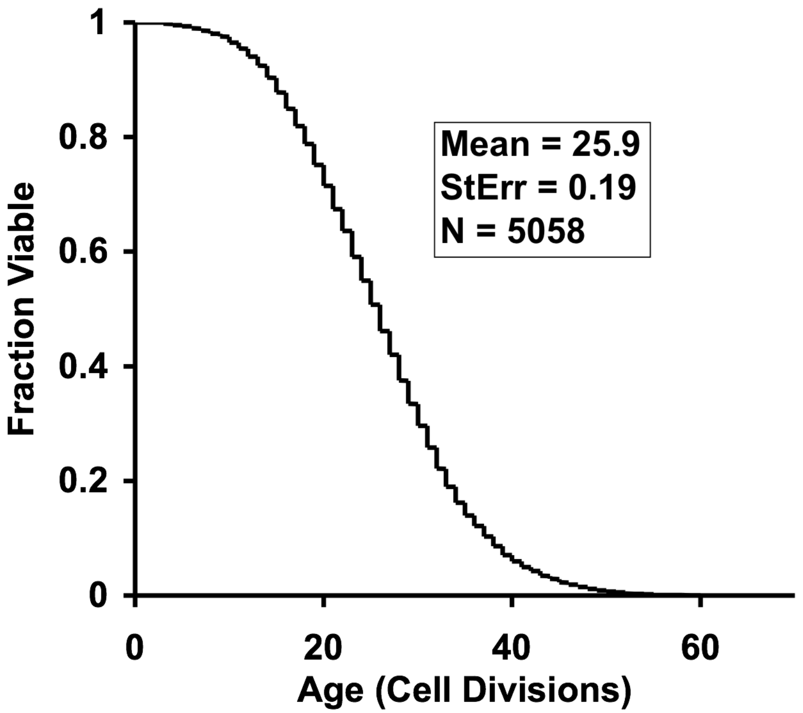

B

5-cell Sets

	< 5	5-10	10-15	15-20	20-25	25-30	30-35	35-40	40-45	45-50	>50
**Sim1**	0	0	0.00248	0.07084	0.3564	0.41992	0.13644	0.0135	0.00042	0	0
**Sim2**	0	0	0.0025	0.06822	0.3542	0.42346	0.13812	0.01298	0.0005	0.00002	0
**Sim3**	0	0.00002	0.0023	0.07014	0.35268	0.42572	0.13572	0.01302	0.0004	0	0
**Sim4**	0	0	0.00234	0.06954	0.35636	0.42012	0.13852	0.01268	0.00044	0	0
**Sim5**	0	0.00002	0.00262	0.07042	0.3533	0.42214	0.1379	0.013	0.0006	0	0
**Sim6**	0	0.00002	0.00212	0.06956	0.35416	0.42228	0.139	0.01252	0.00034	0	0
**Sim7**	0	0	0.00252	0.07178	0.35412	0.4219	0.13542	0.01386	0.0004	0	0
**Sim8**	0	0	0.00242	0.06958	0.3547	0.4224	0.1367	0.01372	0.00046	0.00002	0
**Sim9**	0	0	0.0024	0.0704	0.35672	0.4193	0.1369	0.01388	0.0004	0	0
**Sim10**	0	0	0.00246	0.06816	0.36082	0.41954	0.13486	0.0137	0.00046	0	0

C

10-cell Sets

	< 5	5-10	10-15	15-20	20-25	25-30	30-35	35-40	40-45	45-50	>50
**Sim1**	0	0	0.00002	0.01768	0.37108	0.5353	0.07492	0.001	0	0	0
**Sim2**	0	0	0	0.0179	0.36704	0.53938	0.07488	0.0008	0	0	0
**Sim3**	0	0	0	0.0184	0.36612	0.53952	0.07512	0.00084	0	0	0
**Sim4**	0	0	0	0.01776	0.36542	0.53924	0.07654	0.00104	0	0	0
**Sim5**	0	0	0.00004	0.0176	0.37114	0.53664	0.07372	0.00086	0	0	0
**Sim6**	0	0	0.00002	0.01756	0.37092	0.53622	0.07404	0.00124	0	0	0
**Sim7**	0	0	0.00004	0.0176	0.36386	0.5434	0.0741	0.001	0	0	0
**Sim8**	0	0	0	0.01806	0.36666	0.53812	0.07608	0.00108	0	0	0
**Sim9**	0	0	0	0.01728	0.36746	0.53958	0.07482	0.00086	0	0	0
**Sim10**	0	0	0.00004	0.01792	0.36408	0.54082	0.07622	0.00092	0	0	0
