# Supplementary material for: Shortest-Path Network Analysis Is a Useful Approach toward Identifying Genetic Determinants of Longevity
Source: PLoS One. 2008 Nov 25;3(11):e3802. doi: 10.1371/journal.pone.0003802 (PMC2583956; doi:10.1371/journal.pone.0003802)

Figure S2. Deletion of *YPT6*, a component of the composite shortest-path longevity network, increases replicative life span. Replicative life span is plotted for *ypt6Δ* and for experiment-matched wild type (WT) cells. Replicative life span extension was significant in both mating types ($p < 0.05$, Wilcoxon Rank-Sum Test). Pooled data from both mating types is shown.

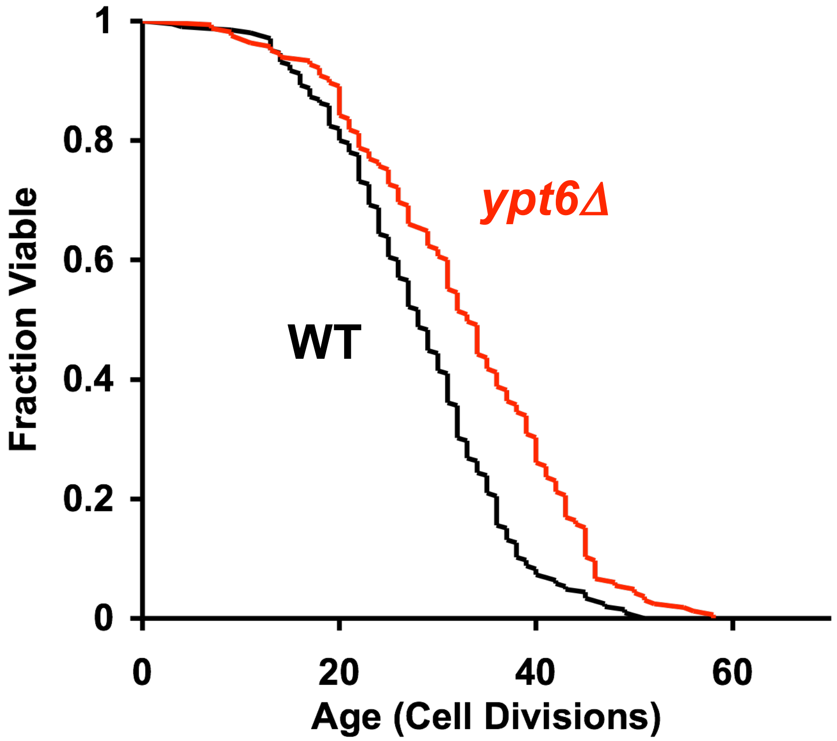

Supplement: Figure S2 — Deletion of YPT6, a component of the composite shortest-path longevity network, increases replicative life span. (0.11 MB PDF) [file pone.0003802.s002.pdf]
