# Supplementary material for: Shortest-Path Network Analysis Is a Useful Approach toward Identifying Genetic Determinants of Longevity
Source: PLoS One. 2008 Nov 25;3(11):e3802. doi: 10.1371/journal.pone.0003802 (PMC2583956; doi:10.1371/journal.pone.0003802)
Supplement: Table S1 — Set of yeast genes reported to be associated with increased replicative life span used to construct the shortest-path longevity network. (0.11 MB PDF) [file pone.0003802.s003.pdf]

Table S1. Set of yeast genes reported to be associated with increased replicative life span used to construct the shortest-path longevity network. This list was derived from the our Aging Genes/Interventions Database (formerly the SAGE KE Genes/Interventions Database [1]; http://www.kaeberleinlab.org/ageid) and from independent literature searches.

ORF	GENE	EFFECT ON RLS	REFERENCE
YNR051C	BRE5	Deletion increases RLS	[2]
YLR310C	CDC25	Point mutation increases RLS	[3]
YJL194W	CDC6	Deletion increases RLS	[4]
YJL005W	CYR1	Point mutation increases RLS	[3]
YDR110W	FOB1	Deletion increases RLS	[5]
YER020W	GPA2	Deletion increases RLS	[3]
YDL035C	GPR1	Deletion increases RLS	[3]
YKL109W	HAP4	Overexpression increases RLS	[3]
YGL253W	HXK2	Deletion increases RLS	[3]
YOR136W	IDH2	Deletion increases RLS	[2]
YHL003C	LAG1	Deletion or overexpression increases RLS	[6]
YOL025W	LAG2	Deletion increases RLS	[7]
YGL178W	MPT5	Overexpression increases RLS	[8]
YPR030W	MRG19	Deletion increases RLS	[9]
YLR285W	NNT1	Overexpression increases RLS	[10]
YOR209C	NPT1	Overexpression increases RLS	[11]
YGL037C	PNC1	Overexpression increases RLS	[10]
YOR101W	RAS1	Deletion increases RLS	[12]
YNL0986	RAS2	Overexpression increases RLS	[12]
YBR267W	REI1	Deletion increases RLS	[2]
YLR371W	ROM2	Deletion increases RLS	[2]
YNL330C	RPD3	Deletion increases RLS	[13]
YDL075W	RPL31A	Deletion increases RLS	[2]
YLR448W	RPL6B	Deletion increases RLS	[2]
YBL103C	RTG3	Deletion increases RLS	[14]
YHR205W	SCH9	Deletion increases RLS	[15]
YOR367W	SCP1	Deletion increases RLS	[16]
YDL042C	SIR2	Overexpression increases RLS	[17]
YDR227W	SIR4	Semi-dominant Sir4-42 allele increases RLS	[18]
YGL115W	SNF4	Deletion increases RLS	[19]
YDR293C	SSD1	SSD1-V allele increases RLS	[20]
YJR066W	TOR1	Deletion increases RLS	[2]
YPL203W	TPK2	Point mutation increases RLS	[3]
YNL229C	URE2	Deletion increases RLS	[2]
YKR042W	UTH1	Deletion increases RLS	[18]
YBR238C	YBR238C	Deletion increases RLS	[2]
YBR255W	YBR255W	Deletion increases RLS	[2]
YBR266C	YBR266C	Deletion increases RLS	[2]
YOR135C	YOR135C	Deletion increases RLS	[2]
YMR273C	ZDS1	Deletion increases RLS	[21]

References

1. Kaeberlein M, Jegalian B, McVey M (2002) AGEID: a database of aging genes and interventions. Mech Ageing Dev 123: 1115-1119.
2. Kaeberlein M, Powers RW, 3rd, Steffen KK, Westman EA, Hu D, et al. (2005) Regulation of yeast replicative life span by TOR and Sch9 in response to nutrients. Science 310: 1193-1196.
3. Lin SJ, Defossez PA, Guarente L (2000) Requirement of NAD and SIR2 for life-span extension by calorie restriction in Saccharomyces cerevisiae. Science 289: 2126-2128.
4. Sinclair DA, Mills K, Guarente L (1997) Accelerated aging and nucleolar fragmentation in yeast sgs1 mutants. Science 277: 1313-1316.
5. Defossez PA, Prusty R, Kaeberlein M, Lin SJ, Ferrigno P, et al. (1999) Elimination of replication block protein Fob1 extends the life span of yeast mother cells. Mol Cell 3: 447-455.
6. D'Mello N P, Childress AM, Franklin DS, Kale SP, Pinswasdi C, et al. (1994) Cloning and characterization of LAG1, a longevity-assurance gene in yeast. J Biol Chem 269: 15451-15459.
7. Childress AM, Franklin DS, Pinswasdi C, Kale S (1996) LAG2, a gene that determines yeast longevity. Microbiology 142 (Pt 8): 2289-2297.
8. Kennedy BK, Gotta M, Sinclair DA, Mills K, McNabb DS, et al. (1997) Redistribution of silencing proteins from telomeres to the nucleolus ia associated with extension of life span in *S. cerevisiae*. Cell 89: 381-391.
9. Kharade SV, Mittal N, Das SP, Sinha P, Roy N (2005) Mrg19 depletion increases S. cerevisiae lifespan by augmenting ROS defence. FEBS Lett 579: 6809-6813.
10. Anderson RM, Bitterman KJ, Wood JG, Medvedik O, Sinclair DA (2003) Nicotinamide and PNC1 govern lifespan extension by calorie restriction in Saccharomyces cerevisiae. Nature 423: 181-185.
11. Anderson RM, Bitterman KJ, Wood JG, Medvedik O, Cohen H, et al. (2002) Manipulation of a nuclear NAD+ salvage pathway delays aging without altering steady-state NAD+ levels. J Biol Chem 277: 18881-18890.
12. Sun J, Kale SP, Childress AM, Pinswasdi C, Jazwinski SM (1994) Divergent roles of RAS1 and RAS2 in yeast longevity. J Biol Chem 269: 18638-18645.
13. Kim S, Benguria A, Lai CY, Jazwinski SM (1999) Modulation of life-span by histone deacetylase genes in Saccharomyces cerevisiae. Mol Biol Cell 10: 3125-3136.
14. Kirchman PA, Kim S, Lai CY, Jazwinski SM (1999) Interorganelle signaling is a determinant of longevity in Saccharomyces cerevisiae. Genetics 152: 179-190.
15. Fabrizio P, Liou LL, Moy VN, Diaspro A, SelverstoneValentine J, et al. (2003) SOD2 functions downstream of Sch9 to extend longevity in yeast. Genetics 163: 35-46.
16. Gourlay CW, Carpp LN, Timpson P, Winder SJ, Ayscough KR (2004) A role for the actin cytoskeleton in cell death and aging in yeast. J Cell Biol 164: 803-809.
17. Kaeberlein M, McVey M, Guarente L (1999) The SIR2/3/4 complex and SIR2 alone promote longevity in Saccharomyces cerevisiae by two different mechanisms. Genes Dev 13: 2570-2580.

18. Kennedy BK, Austriaco NR, Zhang J, Guarente L (1995) Mutation in the silencing gene SIR4 can delay aging in S. cerevisiae. Cell 80: 485-496.
19. Ashrafi K, Lin SS, Manchester JK, Gordon JI (2000) Sip2p and its partner snf1p kinase affect aging in S. cerevisiae. Genes Dev 14: 1872-1885.
20. Kaeberlein M, Andalis AA, Liszt G, Fink GR, Guarente L (2004) Saccharomyces cerevisiae SSD1-V confers longevity by a Sir2p-independent mechanism. Genetics 166: 1661-1672.
21. Roy N, Runge KW (2000) Two paralogs involved in transcriptional silencing that antagonistically control yeast life span. Curr Biol 10: 111-114.
