# Supplementary material for: Shortest-Path Network Analysis Is a Useful Approach toward Identifying Genetic Determinants of Longevity
Source: PLoS One. 2008 Nov 25;3(11):e3802. doi: 10.1371/journal.pone.0003802 (PMC2583956; doi:10.1371/journal.pone.0003802)
Supplement: Table S2 — Relation table for the composite shortest path longevity network. (0.16 MB PDF) [file pone.0003802.s004.pdf]

Table S2. Relation table for the composite shortest path longevity network.

TYPE	RELATION
Binding	ARP3 ---- NOG2
Binding	ACT1 ---- CYR1
Binding	NMD3 ---- REI1
Binding	GFA1 ---- RAD51
Binding	KAP123 ---- RAD51
Binding	RAD51 ---- YHB1
Binding	ACT1 ---- RNA14
Binding	HOS2 ---- TOP1
Binding	GFA1 ---- RPN10
Binding	SLA1 ---- YSC84
Binding	BMH1 ---- SNF4
Binding	GAL83 ---- SNF4
Binding	BOI1 ---- YBR238C
Binding	GFA1 ---- YBR238C
Binding	ARX1 ---- REI1
Binding	HOS2 ---- ZDS1
Binding	CSR2 ---- WHI2
Binding	ARP2 ---- SPS1
Binding	ABP1 ---- SCP1
Binding	SIP2 ---- SNF4
Binding	IDH2 ---- SIP2
Binding	ARP2 ---- GFA1
Binding	BMH2 ---- CSR2
Binding	BMH1 ---- CSR2
Binding	NNT1 ---- TMA19
Binding	CYT2 ---- NNT1
Binding	FOB1 ---- IOC2
Binding	EFT2 ---- RET2
Binding	MOB1 ---- YBR255W
Binding	JSN1 ---- RPL31B
Binding	JSN1 ---- SMT3
Binding	JSN1 ---- NPT1
Binding	PHD1 ---- SMT3
Binding	RPL6B ---- SKG6
Binding	NUP116 ---- SMT3
Binding	LAG1 ---- NUP116
Binding	GPA2 ---- NAF1
Binding	BRE5 ---- UBP3
Binding	CSR2 ---- PDC6
Binding	FHL1 ---- SMT3
Binding	AVT4 ---- LAG1
Binding	LAG1 ---- SWP1
Binding	LAG1 ---- SPC1
Binding	CHS7 ---- LAG1
Binding	GPI8 ---- LAG1
Binding	AGP1 ---- LAG1
Binding	CSG2 ---- LAG1
Binding	BRE5 ---- SPT5
Binding	SPT5 ---- TOM1

Binding	ACT1 ---- SWR1
Binding	ACT1 ---- TAF14
Binding	ACT1 ---- SWC5
Binding	ACT1 ---- VPS71
Binding	SIR4 ---- UBP3
Binding	CDC25 ---- HSP82
Binding	CDC28 ---- MPT5
Binding	FOB1 ---- SIR2
Binding	SSN3 ---- TUP1
Binding	RAP1 ---- SIR4
Binding	HAP4 ---- SNF5
Binding	HAP4 ---- SWI1
Binding	ARP7 ---- HAP4
Binding	HAP4 ---- TAF14
Binding	CDC25 ---- RAS1
Binding	CDC6 ---- ORC2
Binding	ACT1 ---- RVS167
Binding	SIR2 ---- ZDS1
Binding	SIR4 ---- ZDS1
Binding	ABP1 ---- CLA4
Binding	RVS167 ---- YSC84
Binding	SKG6 ---- ZDS1
Binding	HAP4 ---- TRA1
Binding	TOR1 ---- URE2
Binding	ACT1 ---- HTZ1
Binding	ACT1 ---- YAF9
Binding	SMT3 ---- TUP1
Binding	ACT1 ---- SMT3
Binding	SMT3 ---- TAF14
Binding	RPC82 ---- SMT3
Binding	HSP82 ---- SMT3
Binding	SCS2 ---- SMT3
Binding	SEC27 ---- SMT3
Binding	BNR1 ---- CDC25
Binding	SMT3 ---- TOP1
Binding	SMT3 ---- SNF2
Binding	ELG1 ---- SMT3
Binding	SMT3 ---- STM1
Binding	RAP1 ---- SMT3
Binding	SMT3 ---- TRA1
Binding	SMT3 ---- SNF1
Binding	RTN1 ---- SMT3
Binding	ORM2 ---- SMT3
Binding	PMA1 ---- SMT3
Binding	CYR1 ---- SMT3
Binding	SMT3 ---- YAR010C
Binding	SMT3 ---- YBR238C
Binding	CDC25 ---- CYR1
Binding	HSP82 ---- RTG3
Binding	HSP82 ---- TOM1
Binding	NSE3 ---- REI1
Binding	NAF1 ---- RPL6B
Binding	ACT1 ---- HSP82
Binding	HSP82 ---- SIR2

Binding	HSP82 ---- RAS1
Binding	CLB2 ---- SIR4
Binding	CLB2 ---- CSR2
Binding	CDC28 ---- SIR4
Binding	CDC28 ---- CSR2
Binding	BRE5 ---- GCN2
Binding	RVS167 ---- SPS1
Binding	EFT2 ---- HCA4
Binding	CYR1 ---- YKE2
Binding	SIR4 ---- VPS71
Binding	GFA1 ---- URE2
Binding	BRE5 ---- YAR010C
Binding	BRE5 ---- RIM1
Binding	BRE5 ---- YRA1
Binding	BRE5 ---- HYP2
Binding	BRE5 ---- STM1
Binding	BRE5 ---- NIP1
Binding	BRE5 ---- NOP2
Binding	LAG2 ---- TOM1
Binding	BRE5 ---- SGV1
Binding	GFA1 ---- YHB1
Binding	BRE5 ---- IDP3
Binding	MIS1 ---- RPL6B
Binding	IDH2 ---- KAP123
Binding	REF2 ---- RPL6B
Binding	HCA4 ---- RPL6B
Binding	NOP4 ---- RPL6B
Binding	CIC1 ---- RPL31B
Binding	NOP15 ---- RPL31B
Binding	ARX1 ---- RPL31B
Binding	NOG2 ---- RPL31B
Binding	RPL31B ---- TIF4631
Binding	RPL6B ---- SDA1
Binding	APL2 ---- RPL6B
Binding	IDH2 ---- RVS167
Binding	MAK21 ---- RPL31B
Binding	LOC1 ---- RPL31B
Binding	RPL31B ---- SEC31
Binding	REI1 ---- RPL20A
Binding	RPL6B ---- SLA1
Binding	MAS1 ---- RPL6B
Binding	MAK21 ---- RPL6B
Binding	FAS1 ---- RAD51
Binding	CFT1 ---- RPL6B
Binding	RNA14 ---- RPL6B
Binding	DBP8 ---- IDH2
Binding	ACT1 ---- SMI1
Binding	GFA1 ---- IDH2
Binding	GFA1 ---- SEC27
Binding	RPL31B ---- TIF6
Binding	GFA1 ---- KAP123
Binding	EFT2 ---- KAP123
Binding	RPL20B ---- RPL31B
Binding	RPL20A ---- RPL31B

Binding	GIM5 ---- RPL31B
Binding	MAS1 ---- TMA19
Binding	IDH2 ---- SNF4
Binding	ADR1 ---- GFA1
Binding	CYS4 ---- GFA1
Binding	IDH2 ---- YHB1
Binding	IDH2 ---- SNF1
Binding	ACT1 ---- TRA1
Binding	GCN2 ---- NPT1
Binding	CYS4 ---- TMA19
Binding	HXT7 ---- TIF6
Binding	CYS4 ---- HXT7
Binding	HXT7 ---- HYP2
Binding	FKS1 ---- GFA1
Binding	CSR2 ---- NTH1
Binding	GLK1 ---- NTH1
Binding	CIC1 ---- HXT7
Binding	HXT7 ---- NOP2
Binding	PNC1 ---- YPC1
Binding	ARP2 ---- SSD1
Binding	FAS1 ---- REI1
Binding	KAP123 ---- REI1
Binding	CYS4 ---- REI1
Binding	MIS1 ---- REI1
Binding	ARP2 ---- IDH2
Binding	ARP2 ---- SAC6
Binding	CYR1 ---- MOB1
Binding	IDH2 ---- MOB1
Binding	RVS167 ---- SAC6
Binding	ACT1 ---- CFT1
Binding	ARX1 ---- EFT2
Binding	AVT4 ---- CDC25
Binding	CDC25 ---- NAP1
Binding	CDC25 ---- HYP2
Binding	CDC25 ---- SFB3
Binding	ACT1 ---- ARX1
Binding	RAD51 ---- SLA1
Binding	SEC28 ---- SSD1
Binding	CYR1 ---- MAS1
Binding	CYR1 ---- REG1
Binding	CYR1 ---- SNF2
Binding	CYR1 ---- NOP2
Binding	CYR1 ---- SEC27
Binding	CYR1 ---- CYS4
Binding	CYR1 ---- TMA19
Binding	GFA1 ---- NIP1
Binding	BOI1 ---- BOI2
Binding	ARP3 ---- NIP1
Binding	ACT1 ---- NIP1
Binding	STM1 ---- YSC84
Binding	KAP123 ---- ROM2
Binding	ROM2 ---- TIF4631
Binding	SCP1 ---- YSC84
Binding	ARP3 ---- SCP1

Binding	SCP1 ---- SPS1
Binding	HXT7 ---- SCP1
Binding	SCP1 ---- TUP1
Binding	SCS2 ---- TUP1
Binding	RET2 ---- TUP1
Binding	EFT2 ---- TUP1
Binding	EFT2 ---- HOS2
Binding	EFT2 ---- FAS1
Binding	EFT2 ---- YBR238C
Binding	EFT2 ---- YHB1
Binding	EFT2 ---- SEC27
Binding	EFT2 ---- RPN10
Binding	EFT2 ---- ROM2
Binding	GLK1 ---- PNC1
Binding	ACT1 ---- EFT2
Binding	EFT2 ---- ZDS1
Binding	HYP2 ---- SAC6
Binding	SAC6 ---- SWE1
Binding	PRP3 ---- SAC6
Binding	DBP8 ---- REI1
Binding	MAS1 ---- SSD1
Binding	CDC25 ---- MAS1
Binding	HXT7 ---- MAS1
Binding	CSR2 ---- SOK1
Binding	PMA1 ---- SNF4
Binding	ADR1 ---- ARP3
Binding	SEC27 ---- URE2
Binding	ABP1 ---- MAS1
Binding	ABP1 ---- RET2
Binding	ABP1 ---- CDC25
Binding	ABP1 ---- GAS1
Binding	ABP1 ---- TMA19
Binding	IDH2 ---- SEC27
Binding	ABP1 ---- LOC1
Binding	ABP1 ---- HYP2
Binding	ABP1 ---- SFB3
Binding	ABP1 ---- CYS4
Binding	BMH2 ---- GFA1
Binding	GFA1 ---- RPC82
Binding	GFA1 ---- PMA1
Binding	FAS1 ---- GFA1
Binding	GFA1 ---- NAP1
Binding	ARP3 ---- GFA1
Binding	BMH1 ---- GFA1
Binding	CYS4 ---- TPK2
Binding	ARP7 ---- GFA1
Binding	CDC6 ---- RPN10
Binding	ADR1 ---- IDH2
Binding	IDH2 ---- URE2
Binding	IDH2 ---- RPC82
Binding	HYP2 ---- IDH2
Binding	IDH2 ---- RPN10
Binding	RVS167 ---- URE2
Binding	GFA1 ---- RVS167

Binding	HXT7 ---- RVS167
Binding	ARP3 ---- RVS167
Binding	SSD1 ---- YHB1
Binding	CDC25 ---- GIN4
Binding	ACT1 ---- RVS161
Binding	ACT1 ---- KAP123
Binding	ACT1 ---- GAL83
Binding	SSD1 ---- TIF4631
Binding	CDC25 ---- TMA19
Binding	LOC1 ---- TMA19
Binding	SEC27 ---- TMA19
Binding	HYP2 ---- TMA19
Binding	HXT7 ---- TMA19
Binding	TIF4631 ---- TMA19
Binding	SFB3 ---- TMA19
Binding	SSD1 ---- TMA19
Binding	HYP2 ---- TPK2
Binding	IDH2 ---- YOR135C
Binding	HXT7 ---- PDC6
Binding	CYS4 ---- SSD1
Binding	CDC25 ---- CYS4
Binding	GFA1 ---- YRA1
Binding	EFT2 ---- YRA1
Binding	ARP3 ---- NOP4
Binding	CYR1 ---- NOP4
Binding	CDC25 ---- LOC1
Binding	HXT7 ---- LOC1
Binding	ADR1 ---- URE2
Binding	ADR1 ---- CSR2
Binding	CSR2 ---- HYP2
Binding	BOI2 ---- CSR2
Binding	CSR2 ---- REG1
Binding	CDC25 ---- GAS1
Binding	GAS1 ---- TMA19
Binding	BNR1 ---- CSR2
Binding	GAS1 ---- HXT7
Binding	CDC28 ---- URE2
Binding	RPC82 ---- URE2
Binding	YRA1 ---- ZDS1
Binding	ADA2 ---- EFT2
Binding	CDC25 ---- HXT7
Binding	HXT7 ---- SFB3
Binding	HXT7 ---- SCS2
Binding	HXT7 ---- SLA1
Binding	HXT7 ---- NOP15
Binding	HXT7 ---- NMD3
Binding	NMD3 ---- SSD1
Binding	GAL83 ---- IDH2
Binding	MIS1 ---- ROM2
Binding	EFT2 ---- FKS1
Binding	FKS1 ---- IDH2
Binding	IOC2 ---- RPL31B
Binding	RAS2 ---- RTG3
Binding	FOB1 ---- RAD53

Binding SIP4 ---- UME6
Binding HOS2 ---- RIM1
Binding RAD51 ---- SMT3
Binding RAP1 ---- SIR2
Binding SIP2 ---- SIP4
Binding ACT1 ---- YRA1
Binding ACT1 ---- SLA1
Binding RIM1 ---- TUP1
Binding TOM1 ---- ubiquitin
Binding ADA2 ---- HAP4
Binding BRE5 ---- ubiquitin
Binding ACT1 ---- FHL1
Binding RTG3 ---- TUP1
Binding CYC1 ---- CYT2
Binding SOK1 ---- SPS1
Binding RAD51 ---- SCP1
Binding CYC1 ---- HAP4
Binding ABP1 ---- BNR1
Binding transcription activating factor ---- TUP1
Binding HAP4 ---- transcription activating factor
Binding ACT1 ---- BNR1
Binding MAS1 ---- RAD51
Binding ELP4 ---- HAP4
Binding GCN4 ---- HAP4
Binding HAP4 ---- SWI5
Binding transcription activating factor ---- URE2
Binding RIM1 ---- URE2
Binding HAP4 ---- SNF2
Binding EFT2 ---- HYP2
DirectRegulation CDC25 --+> RAS2
DirectRegulation RAS2 --+> CYR1
DirectRegulation HOS2 --+> TUP1
DirectRegulation SIP4 ---> GAL83
DirectRegulation HOS2 ---> UME6
DirectRegulation ABP1 ---> SLA1
DirectRegulation ARP2 ---> ACT1
DirectRegulation ABP1 --+> ARP2
DirectRegulation SNF1 --+> SIP4
DirectRegulation STM1 ---| GPA2
DirectRegulation SIR4 --+> SIR2
DirectRegulation ubiquitin --+> SMT3
DirectRegulation GPR1 --+> GPA2
DirectRegulation ARP3 --+> ARP2
DirectRegulation SNF4 ---> SNF1
DirectRegulation SHR5 ---| RAS2
DirectRegulation ABP1 ---> RVS167
DirectRegulation SLA1 --+> ARP3
DirectRegulation SWI5 --+> CDC6
DirectRegulation SSN3 --+> SIP4
DirectRegulation RAS1 ---> RAS2
DirectRegulation REG1 ---> SNF4
DirectRegulation RAS2 --+> GPA2
DirectRegulation GLK1 --+> SNF1
DirectRegulation SCP1 ---| ACT1

DirectRegulation	CDC28 ---| CDC6
DirectRegulation	NPT1 --+> PNC1
DirectRegulation	TUP1 ---> MATALPHA2
DirectRegulation	CLB2 --+> CDC6
Expression	glucose ---| SIP4
Expression	glucose ---| HAP4
Expression	glucose ---| HXT7
Expression	CDC6 ---> GAL3
Expression	ZDS1 ---| SWE1
Expression	superoxide ---| UTH1
Expression	glucose ---| GPR1
Expression	TPK2 ---> NTH1
Expression	SIR2 --+> PNC1
GeneticInteraction	ACT1 ---- RNA14
GeneticInteraction	ACT1 ---- SWP1
GeneticInteraction	HOS2 ---- SET2
GeneticInteraction	CLA4 ---- URE2
GeneticInteraction	BRE5 ---- SET2
GeneticInteraction	ARP2 ---- SAC6
GeneticInteraction	ARP2 ---- UTH1
GeneticInteraction	BIM1 ---- BRE5
GeneticInteraction	RIC1 ---- SNF4
GeneticInteraction	BRE5 ---- RIC1
GeneticInteraction	SNF4 ---- YPT6
GeneticInteraction	BRE5 ---- YPT6
GeneticInteraction	CTF4 ---- RAD51
GeneticInteraction	BRE5 ---- CTF4
GeneticInteraction	YBR255W ---- YKE2
GeneticInteraction	SSD1 ---- YKE2
GeneticInteraction	HOS2 ---- YKE2
GeneticInteraction	GLK1 ---- YKE2
GeneticInteraction	PAC10 ---- YBR255W
GeneticInteraction	PAC10 ---- SSD1
GeneticInteraction	HOS2 ---- PAC10
GeneticInteraction	GLK1 ---- PAC10
GeneticInteraction	GIM5 ---- YBR255W
GeneticInteraction	GIM5 ---- SSD1
GeneticInteraction	GIM5 ---- HOS2
GeneticInteraction	GIM5 ---- GLK1
GeneticInteraction	BRE5 ---- CSM3
GeneticInteraction	POL32 ---- RAD51
GeneticInteraction	BRE5 ---- TOP1
GeneticInteraction	RAS1 ---- RAS2
GeneticInteraction	CHS3 ---- SAC6
GeneticInteraction	BRE5 ---- CHS3
GeneticInteraction	CHS7 ---- SAC6
GeneticInteraction	FKS1 ---- ROM2
GeneticInteraction	BRE5 ---- FKS1
GeneticInteraction	CSR2 ---- FKS1
GeneticInteraction	GAS1 ---- SSD1
GeneticInteraction	GAS1 ---- ROM2
GeneticInteraction	HOC1 ---- SAC6
GeneticInteraction	BRE5 ---- HOC1
GeneticInteraction	SAC6 ---- SKT5

GeneticInteraction	BRE5 ---- SKT5
GeneticInteraction	ROM2 ---- SMI1
GeneticInteraction	BRE5 ---- SMI1
GeneticInteraction	RVS161 ---- YBR255W
GeneticInteraction	RVS167 ---- YBR255W
GeneticInteraction	ARR4 ---- BRE5
GeneticInteraction	ARR4 ---- YBR255W
GeneticInteraction	BRE5 ---- SEC28
GeneticInteraction	BRE5 ---- RMD7
GeneticInteraction	BRE5 ---- SPF1
GeneticInteraction	BRE5 ---- MDM39
GeneticInteraction	BRE5 ---- RGP1
GeneticInteraction	BRE5 ---- SCS7
GeneticInteraction	BRE5 ---- RET2
GeneticInteraction	BRE5 ---- SEC27
GeneticInteraction	BRE5 ---- STE24
GeneticInteraction	BRE5 ---- ERV14
GeneticInteraction	BRE5 ---- PSD2
GeneticInteraction	DRS2 ---- YBR255W
GeneticInteraction	GAS1 ---- LAG1
GeneticInteraction	LAG1 ---- PMA1
GeneticInteraction	LAG1 ---- MDM39
GeneticInteraction	LAG1 ---- ORM2
GeneticInteraction	LAG1 ---- SEC28
GeneticInteraction	LAG1 ---- SCS7
GeneticInteraction	LAG1 ---- RGP1
GeneticInteraction	LAG1 ---- RIC1
GeneticInteraction	RTN1 ---- YBR255W
GeneticInteraction	SCS2 ---- YBR255W
GeneticInteraction	SHR5 ---- YBR255W
GeneticInteraction	SSO1 ---- YBR255W
GeneticInteraction	SUR2 ---- YBR255W
GeneticInteraction	SUR4 ---- YBR255W
GeneticInteraction	TPO1 ---- YBR255W
GeneticInteraction	CAT8 ---- SNF4
GeneticInteraction	CDC25 ---- RAS1
GeneticInteraction	CDC6 ---- CLB2
GeneticInteraction	CDC6 ---- SWI5
GeneticInteraction	CYR1 ---- RAS2
GeneticInteraction	GPA2 ---- RAS2
GeneticInteraction	HAP4 ---- MBR1
GeneticInteraction	ATP1 ---- RAS2
GeneticInteraction	CDC25 ---- RAS2
GeneticInteraction	MBR1 ---- SCH9
GeneticInteraction	CDC25 ---- SCH9
GeneticInteraction	CYR1 ---- SCH9
GeneticInteraction	SAC6 ---- SCP1
GeneticInteraction	SCS2 ---- SIR2
GeneticInteraction	CAT8 ---- SIP4
GeneticInteraction	HOS2 ---- SKT5
GeneticInteraction	SMT3 ---- TOM1
GeneticInteraction	SNF1 ---- SNF4
GeneticInteraction	SSD1 ---- YPT6
GeneticInteraction	GPR1 ---- TPK2

GeneticInteraction	RAS2 ---- TPK2
GeneticInteraction	MPT5 ---- STM1
GeneticInteraction	STM1 ---- TOM1
GeneticInteraction	FKS1 ---- ZDS1
GeneticInteraction	CDC28 ---- ZDS1
GeneticInteraction	BOI1 ---- BOI2
GeneticInteraction	CHS3 ---- GFA1
GeneticInteraction	PUT3 ---- URE2
GeneticInteraction	ASF1 ---- RAD51
GeneticInteraction	MPT5 ---- SSD1
GeneticInteraction	GPA2 ---- SCH9
GeneticInteraction	ELG1 ---- RAD51
GeneticInteraction	ACT1 ---- RVS167
GeneticInteraction	ABP1 ---- SLA1
GeneticInteraction	CYR1 ---- TOM1
GeneticInteraction	CYR1 ---- GPA2
GeneticInteraction	GPA2 ---- GPR1
GeneticInteraction	RAS1 ---- REG1
GeneticInteraction	RAS2 ---- REG1
GeneticInteraction	HOS2 ---- RAD6
GeneticInteraction	HOS2 ---- SWI1
GeneticInteraction	BMH1 ---- RTG3
GeneticInteraction	BMH2 ---- RTG3
GeneticInteraction	SCH9 ---- TOM1
GeneticInteraction	HOS2 ---- RAP1
GeneticInteraction	RAS2 ---- SHR5
GeneticInteraction	HOS2 ---- SIR2
GeneticInteraction	ACT1 ---- SUR2
GeneticInteraction	ACT1 ---- SUR4
GeneticInteraction	SWI1 ---- TUP1
GeneticInteraction	GPA2 ---- TPK2
GeneticInteraction	SNF1 ---- TUP1
GeneticInteraction	CLB2 ---- RAS2
GeneticInteraction	CSR2 ---- GAL3
GeneticInteraction	CDC6 ---- SIR2
GeneticInteraction	CDC6 ---- SIR4
GeneticInteraction	SOK1 ---- TPK2
GeneticInteraction	WHI2 ---- ZDS1
GeneticInteraction	SWE1 ---- ZDS1
GeneticInteraction	NAP1 ---- REI1
GeneticInteraction	CLA4 ---- REI1
GeneticInteraction	GIN4 ---- REI1
GeneticInteraction	GCN4 ---- URE2
GeneticInteraction	ACT1 ---- RVS161
GeneticInteraction	SSD1 ---- TOR1
GeneticInteraction	REG1 ---- SNF4
GeneticInteraction	BMH1 ---- CDC6
GeneticInteraction	NPT1 ---- SIR2
GeneticInteraction	GPA2 ---- RAS1
GeneticInteraction	RAS1 ---- TPK2
GeneticInteraction	BRE5 ---- SFB3
GeneticInteraction	BMH1 ---- CDC25
GeneticInteraction	BMH2 ---- CDC25
GeneticInteraction	LAG1 ---- YPC1

GeneticInteraction	BNA2 ---- NPT1
GeneticInteraction	CDC6 ---- ORC2
GeneticInteraction	RAS2 ---- SSN3
GeneticInteraction	FOB1 ---- GLK1
GeneticInteraction	FOB1 ---- GPA2
GeneticInteraction	FOB1 ---- SIR2
GeneticInteraction	GLK1 ---- SIR2
GeneticInteraction	GPA2 ---- SIR2
GeneticInteraction	CDC6 ---- RAD53
GeneticInteraction	FHL1 ---- SCH9
GeneticInteraction	RAS2 ---- ROM2
GeneticInteraction	ROM2 ---- TPK2
GeneticInteraction	ABP1 ---- RVS161
GeneticInteraction	IDH2 ---- IDP3
GeneticInteraction	CYR1 ---- REG1
GeneticInteraction	SAC6 ---- SWE1
GeneticInteraction	CSG2 ---- HOS2
GeneticInteraction	ASF1 ---- SIR2
GeneticInteraction	SPF1 ---- TUP1
GeneticInteraction	HYP2 ---- ZDS1
GeneticInteraction	HYP2 ---- ROM2
GeneticInteraction	HYP2 ---- SSD1
GeneticInteraction	RAD18 ---- RAD51
GeneticInteraction	ABP1 ---- ARP2
GeneticInteraction	ARP2 ---- ARP3
GeneticInteraction	ARP6 ---- BRE5
GeneticInteraction	BRE5 ---- HTZ1
GeneticInteraction	BRE5 ---- SWR1
GeneticInteraction	BRE5 ---- YAF9
GeneticInteraction	BRE5 ---- SWC5
GeneticInteraction	BRE5 ---- VPS72
GeneticInteraction	BRE5 ---- VPS71
GeneticInteraction	BRE5 ---- ELG1
GeneticInteraction	ARP6 ---- HOS2
GeneticInteraction	HOS2 ---- HTZ1
GeneticInteraction	HOS2 ---- SWR1
GeneticInteraction	HOS2 ---- YAF9
GeneticInteraction	HOS2 ---- SWC5
GeneticInteraction	HOS2 ---- VPS72
GeneticInteraction	HOS2 ---- VPS71
GeneticInteraction	BRE5 ---- ORC2
GeneticInteraction	CDC6 ---- CTF4
GeneticInteraction	ELP4 ---- HOS2
GeneticInteraction	BIM1 ---- RAD51
GeneticInteraction	BIM1 ---- HOS2
GeneticInteraction	HSP82 ---- RTG3
GeneticInteraction	HSP82 ---- SNF4
GeneticInteraction	HSP82 ---- UTH1
GeneticInteraction	HSP82 ---- ROM2
GeneticInteraction	HSP82 ---- RAS2
GeneticInteraction	BRE5 ---- HSP82
GeneticInteraction	CSR2 ---- HSP82
GeneticInteraction	CDC6 ---- CSM3
GeneticInteraction	CDC6 ---- RPL20B

GeneticInteraction	CDC6 ---- CLA4
GeneticInteraction	ASF1 ---- BRE5
GeneticInteraction	ASF1 ---- NPT1
GeneticInteraction	ASF1 ---- HOS2
GeneticInteraction	CSM3 ---- NPT1
GeneticInteraction	CSM3 ---- RAD51
GeneticInteraction	CTF4 ---- NPT1
GeneticInteraction	BRE5 ---- CCS1
GeneticInteraction	CCS1 ---- NPT1
GeneticInteraction	CCS1 ---- RAD51
GeneticInteraction	MDM39 ---- RAD51
GeneticInteraction	HOS2 ---- MDM39
GeneticInteraction	MDM39 ---- SAC6
GeneticInteraction	CLA4 ---- NPT1
GeneticInteraction	CLB2 ---- NPT1
GeneticInteraction	ELG1 ---- NPT1
GeneticInteraction	MRPL37 ---- NPT1
GeneticInteraction	NPT1 ---- POL32
GeneticInteraction	NPT1 ---- RAD18
GeneticInteraction	NPT1 ---- RAD51
GeneticInteraction	NPT1 ---- REF2
GeneticInteraction	NPT1 ---- RGP1
GeneticInteraction	NPT1 ---- RIC1
GeneticInteraction	HOS2 ---- NPT1
GeneticInteraction	NPT1 ---- SET2
GeneticInteraction	NPT1 ---- SMI1
GeneticInteraction	BRE5 ---- POL32
GeneticInteraction	BRE5 ---- RAD18
GeneticInteraction	CLA4 ---- RAD51
GeneticInteraction	RAD51 ---- RAD6
GeneticInteraction	RAD51 ---- RMD7
GeneticInteraction	RAD51 ---- RPN10
GeneticInteraction	BRE5 ---- RAD53
GeneticInteraction	BRE5 ---- RAD6
GeneticInteraction	NPT1 ---- RAD6
GeneticInteraction	RAD6 ---- RAS2
GeneticInteraction	RAD6 ---- SAC6
GeneticInteraction	NPT1 ---- RMD7
GeneticInteraction	HOS2 ---- RMD7
GeneticInteraction	RMD7 ---- SAC6
GeneticInteraction	BRE5 ---- RPN10
GeneticInteraction	HOS2 ---- RPN10
GeneticInteraction	RPN10 ---- UTH1
GeneticInteraction	BRE5 ---- SWC3
GeneticInteraction	HOS2 ---- SWC3
GeneticInteraction	BOI1 ---- BOI2
GeneticInteraction	CSG2 ---- HOS2
GeneticInteraction	ELG1 ---- RAD51
GeneticInteraction	POL32 ---- RAD51
GeneticInteraction	BRE5 ---- RIC1
GeneticInteraction	CTF4 ---- RAD51
MolSynthesis	ACT1 ---> superoxide
MolSynthesis	GLK1 ---| glucose
MolSynthesis	CDC28 ---| CDC6

MolSynthesis	TUP1 ---| glucose
MolSynthesis	CSR2 ---> nitrogen
MolSynthesis	URE2 ---> nitrogen
MolTransport	UME6 ---> HOS2
MolTransport	MPT5 ---> SIR4
MolTransport	CDC28 ---> CDC6
MolTransport	SNF4 ---> SNF1
MolTransport	CLB2 ---> ACT1
MolTransport	RIM1 ---> TUP1
MolTransport	MATALPHA2 ---> TUP1
PromoterBinding	ATP1 ---- HAP4
PromoterBinding	SIP4 ---- YBR266C
PromoterBinding	MRPL37 ---- SIP4
PromoterBinding	HAP4 ---- MATALPHA2
PromoterBinding	HAP4 ---- MATALPHA1
PromoterBinding	GPR1 ---- SWI5
PromoterBinding	FHL1 ---- RPL31B
PromoterBinding	RAP1 ---- RPL31B
PromoterBinding	EFT2 ---- FHL1
PromoterBinding	HAP4 ---- PRP3
PromoterBinding	GCN4 ---- SMT3
PromoterBinding	ERG26 ---- HAP4
PromoterBinding	ERG26 ---- RTG3
PromoterBinding	RAP1 ---- SIP4
PromoterBinding	BNA2 ---- HAP4
PromoterBinding	HAP4 ---- PUT3
PromoterBinding	HAP4 ---- PHD1
PromoterBinding	ACE2 ---- UTH1
PromoterBinding	FHL1 ---- RPL6B
PromoterBinding	RAP1 ---- RPL6B
PromoterBinding	CSR2 ---- RAP1
PromoterBinding	HAP4 ---- RPC82
ProtModification	REG1 ---> GLK1
ProtModification	SIR2 ---> SIR4
Regulation	glucose --+> GLK1
Regulation	BMH2 ---> grow
Regulation	CLA4 ---> budding
Regulation	TUP1 ---> proliferation
Regulation	ACT1 ---> endocytosis
Regulation	BNR1 ---> cytoskeleton
Regulation	GPA2 ---> cell survival
Regulation	SIR2 --+> cell survival
Regulation	FOB1 ---> senescence
Regulation	MOB1 ---> cytoskeleton
Regulation	RAS2 ---> cell survival
Regulation	URE2 ---> proliferation
Regulation	GPR1 ---> invasive growth
Regulation	SEC31 ---> budding
Regulation	REG1 ---> grow
Regulation	CDC28 ---> budding
Regulation	GLK1 ---| apoptosis
Regulation	SPS1 ---> apoptosis
Regulation	REG1 ---> GFA1
Regulation	BNR1 ---> budding

Regulation	GCN4 ---> budding
Regulation	CDC6 ---> senescence
Regulation	RAD51 ---> apoptosis
Regulation	RVS161 ---> cytoskeleton
Regulation	GCN4 ---> grow
Regulation	CLB2 ---> grow
Regulation	RAD51 ---> proliferation
Regulation	LAG1 ---> senescence
Regulation	URE2 ---> autophagy
Regulation	SMT3 ---> apoptosis
Regulation	NUP116 ---> grow
Regulation	ACT1 ---> cell survival
Regulation	EFT2 ---> synthesis
Regulation	GPA2 ---> invasive growth
Regulation	RIM1 ---> grow
Regulation	SIR2 ---> synthesis
Regulation	SPF1 ---> grow
Regulation	RIM1 ---| CDC6
Regulation	SNF1 ---> grow
Regulation	CDC6 ---> grow
Regulation	CLB2 ---> budding
Regulation	CYR1 ---> grow
Regulation	ARP3 ---> endocytosis
Regulation	RAS1 ---> proliferation
Regulation	ACT1 ---> synthesis
Regulation	RAS2 ---> budding
Regulation	ARP3 ---> cell survival
Regulation	ACT1 ---> apoptosis
Regulation	SIR2 ---| chromosomal DNA replication
Regulation	RAS2 ---> proliferation
Regulation	SPT5 ---> grow
Regulation	NAP1 ---> grow
Regulation	RAS2 ---> grow
Regulation	HSP82 ---> cytoskeleton
Regulation	ZDS1 ---> grow
Regulation	RAS1 ---> grow
Regulation	GPA2 ---| MCD4
Regulation	TUP1 ---> fermentation
Regulation	SIP4 --+> MCD4
Regulation	RVS167 ---> cytoskeleton
Regulation	TOR1 ---> budding
Regulation	MPT5 ---| senescence
Regulation	SMT3 ---> budding
Regulation	NMD3 ---> grow
Regulation	BNR1 ---> grow
Regulation	YPT6 ---> budding
Regulation	HOS2 ---> senescence
Regulation	FKS1 ---> budding
Regulation	RAD51 ---> growth rate
Regulation	CDC6 --+> chromosomal DNA replication
Regulation	MCD4 ---> grow
Regulation	GIN4 ---> grow
Regulation	SIR2 --+> senescence
Regulation	SIR2 ---> budding

Regulation	GAS1 ---> grow
Regulation	FOB1 ---> growth rate
Regulation	synthase ---> grow
Regulation	BOI2 ---> grow
Regulation	SCH9 ---> cell survival
Regulation	YHB1 ---> grow
Regulation	SWE1 ---> budding
Regulation	CDC28 ---> ACT1
Regulation	RAS2 ---> senescence
Regulation	HSP82 ---> grow
Regulation	HSP82 ---> budding
Regulation	TOP1 ---> grow
Regulation	MCD4 ---> budding
Regulation	ubiquitin ---> budding
Regulation	SIR4 ---> senescence
Regulation	RAP1 ---> grow
Regulation	DRS2 ---> budding
Regulation	HOS2 ---> synthesis
Regulation	MATALPHA1 ---> grow
Regulation	ATP1 ---> grow
Regulation	CLA4 ---> ACT1
Regulation	SIR2 ---> grow
Regulation	TRA1 ---> grow
Regulation	CDC6 ---> proliferation
Regulation	CHS3 ---> grow
Regulation	transcription activating factor ---> grow
Regulation	SAC6 ---> endocytosis
Regulation	RAD51 ---> cell survival
Regulation	CDC6 ---> budding
Regulation	ubiquitin ---> grow
Regulation	BOI1 ---> grow
Regulation	CDC28 ---> grow
Regulation	HYP2 ---> grow
Regulation	TOR1 ---> grow
Regulation	FOB1 ---> grow
Regulation	SSO1 ---> grow
Regulation	GPA2 --+> SCH9
Regulation	RAD51 ---> chromosomal DNA replication
Regulation	GCN2 ---> grow
Regulation	URE2 ---> growth rate
Regulation	PSD2 ---> grow
Regulation	STM1 ---| TOM1
Regulation	heme --+> HAP4
Regulation	BMH1 ---> budding
Regulation	SNF4 ---> senescence
Regulation	TPO1 ---> grow
Regulation	SUR4 ---> grow
Regulation	ACT1 ---> invasive growth
Regulation	PMA1 ---> grow
Regulation	CDC6 --+> RIM1
Regulation	SMT3 ---> grow
Regulation	NPT1 --+> senescence
Regulation	GLK1 ---> respiration
Regulation	NPT1 ---> SIR2

Regulation CDC28 --+> cytoskeleton
Regulation LAG1 ---> synthase
Regulation SPC1 ---> grow
Regulation SMT3 ---> cell survival
Regulation SIR2 ---> respiration
Regulation TMA19 ---| apoptosis
Regulation FHL1 ---> grow
Regulation HOS2 ---> cell survival
Regulation BMH2 ---> budding
Regulation CDC6 --+> RAP1
Regulation YPT6 ---> grow
Regulation NPT1 --+> cell survival
Regulation STM1 ---> grow
Regulation SIR2 ---| apoptosis
Regulation FKS1 ---> grow
Regulation TMA19 ---> cell survival
Regulation HXT7 --+> growth rate
Regulation GAL3 ---> grow
Regulation PNC1 ---> senescence
Regulation HAP4 ---> YHB1
Regulation NSE3 ---> grow
Regulation SUR2 ---> grow
Regulation HOS2 ---> proliferation
Regulation HAP4 ---> respiration
Regulation SGV1 ---> grow
Regulation RAD6 ---> budding
Regulation ABP1 ---> endocytosis
Regulation RAS1 ---> senescence
Regulation SIR4 ---> budding
Regulation STE24 ---> grow
Regulation UTH1 ---> autophagy
Regulation CDC25 --+> synthase
Regulation SEC28 ---> budding
Regulation MATALPHA2 ---> budding
Regulation UTH1 ---> chromosomal DNA replication
Regulation RIC1 ---> grow
Regulation SLA1 ---> cytoskeleton
Regulation SPC1 ---> budding
Regulation CLA4 ---> cytoskeleton
Regulation HAP4 ---> growth rate
Regulation CYC1 ---> grow
Regulation AGP1 ---> grow
Regulation GIN4 ---> budding
Regulation SSN3 ---> grow
Regulation HOS2 ---> growth rate
Regulation TMA19 ---> proliferation
Regulation WHI2 ---> budding
Regulation GLK1 ---> proliferation
Regulation transcription activating factor ---> budding
Regulation BIM1 ---> budding
Regulation CYR1 ---> budding
Regulation TOR1 ---| autophagy
Regulation RAS2 ---> growth rate
Regulation RVS167 ---> budding

Regulation	ARP2 ---> budding
Regulation	CTF4 ---> budding
Regulation	ACT1 --+> ARP2
Regulation	ABP1 ---> chromosomal DNA replication
Regulation	BIM1 ---> grow
Regulation	RAS2 --+> apoptosis
Regulation	RVS167 ---> grow
Regulation	MOB1 ---> budding
Regulation	HXT7 ---> fermentation
Regulation	CYC1 ---> budding
Regulation	SEC27 ---> budding
Regulation	GLK1 ---> senescence
Regulation	UME6 ---> grow
Regulation	ZDS1 ---> senescence
Regulation	synthase ---> budding
Regulation	URE2 ---> fermentation
Regulation	RAS2 ---> cytoskeleton
Regulation	SNF5 ---> grow
Regulation	SSD1 ---> grow
Regulation	SCH9 ---> growth rate
Regulation	SCH9 ---> senescence
Regulation	YSC84 ---> endocytosis
Regulation	CLA4 ---> grow
Regulation	TOR1 ---> synthesis
Regulation	TIF6 ---> grow
Regulation	ERV14 ---> budding
Regulation	ARP2 ---> grow
Regulation	HAP4 ---> fermentation
Regulation	ARP3 ---> synthesis
Regulation	ACE2 ---> grow
Regulation	GPA2 ---> senescence
Regulation	TMA19 ---> grow
Regulation	SMT3 ---> chromosomal DNA replication
Regulation	CDC6 ---> apoptosis
Regulation	HOS2 ---| SWI5
Regulation	SSD1 ---> senescence
Regulation	SWE1 ---> grow
Regulation	ACT1 ---> chromosomal DNA replication
Regulation	ORC2 ---> budding
Regulation	SPS1 ---> MCD4
Regulation	YRA1 ---> grow
Regulation	SCP1 ---> budding
Regulation	SPS1 ---> growth rate
Regulation	GPR1 ---> senescence
Regulation	ROM2 ---> grow
Regulation	SMI1 ---| budding
Regulation	ACT1 --+> proliferation
Regulation	glucose --+> TPK2
Regulation	LAG1 --+> endocytosis
Regulation	CDC6 ---> synthesis
Regulation	RAS1 --+> invasive growth
Regulation	ACE2 --+> CDC6
Regulation	SIP4 ---> invasive growth
Regulation	RAS2 --+> invasive growth

Regulation HOS2 ---| MCD4
Regulation RAS1 ---> cytoskeleton
Regulation glucose --+> RAS2
Regulation TPK2 ---> growth rate
Regulation TPK2 ---> RIM1
Regulation glucose ---> SNF4
Regulation SET2 ---> budding
Regulation APL2 ---> grow
Regulation TPK2 ---> invasive growth
Regulation CDC6 ---> CDC28
Regulation LOC1 ---> budding
Regulation RAD51 ---> senescence
Regulation ABP1 ---> growth rate
Regulation nitrogen --+> GPA2
Regulation FOB1 ---> proliferation
Regulation HAP4 ---> synthesis
Regulation URE2 ---> invasive growth
Regulation PNC1 --+> SIR2
Regulation ROM2 ---> proliferation
Regulation GCN2 ---> budding
Regulation FOB1 ---> synthesis
Regulation URE2 ---> grow
Regulation GLK1 --+> REG1
Regulation GPA2 ---> grow
Regulation HOS2 ---> grow
Regulation SCP1 ---> cytoskeleton
Regulation HAP4 ---> senescence
Regulation SNF1 ---> budding
Regulation SSD1 ---> proliferation
Regulation BOI1 ---> budding
Regulation LAG1 ---> apoptosis
Regulation ACE2 ---> budding
Regulation ORC2 ---> grow
Regulation ACT1 ---> respiration
Regulation SIR2 ---> proliferation
Regulation CDC25 ---> invasive growth
Regulation SDA1 ---> budding
Regulation GCN4 --+> URE2
Regulation YKE2 ---> budding
Regulation ROM2 ---> endocytosis
Regulation RAP1 ---> budding
Regulation BOI1 ---> proliferation
Regulation RAD6 ---> grow
Regulation REI1 ---| SWE1
Regulation LAG1 ---> proliferation
Regulation GCN4 --+> IDH2
Regulation TOR1 ---> proliferation
Regulation SDA1 ---> grow
Regulation RAS2 ---> GCN4
Regulation GPI8 ---> grow
Regulation SIR2 ---> fermentation
Regulation TPK2 ---> budding
Regulation RAS2 ---| MCD4
Regulation SCP1 ---> grow

Regulation FOB1 ---| chromosomal DNA replication
Regulation heme ---> ACT1
Regulation SNF1 --+> SNF4
Regulation SIP4 ---> fermentation
Regulation RAS1 ---> MCD4
Regulation TPK2 ---> fermentation
Regulation TPK2 --+> transcription activating factor
Regulation BMH1 ---> grow
Regulation TUP1 ---> cell survival
Regulation SSD1 ---> cell survival
Regulation HOS2 ---> SIR2
Regulation HAP4 ---> cell survival
Regulation ABP1 ---> ARP2
Regulation TOR1 ---> senescence
Regulation EFT2 ---> apoptosis
Regulation ARP2 ---> ACT1
Regulation ACT1 ---> senescence
Regulation TPK2 ---> apoptosis
Regulation EFT2 ---> cell survival
Regulation EFT2 ---> autophagy
Regulation GCN4 --+> TUP1
Regulation ACT1 ---> autophagy
Regulation SCH9 ---> ADR1
