# Supplementary material for: Shortest-Path Network Analysis Is a Useful Approach toward Identifying Genetic Determinants of Longevity
Source: PLoS One. 2008 Nov 25;3(11):e3802. doi: 10.1371/journal.pone.0003802 (PMC2583956; doi:10.1371/journal.pone.0003802)
Supplement: Table S3 — Components of the Binding shortest-path longevity network. (0.10 MB PDF) [file pone.0003802.s005.pdf]

Table S3. Components of the Binding shortest-path longevity network. The Binding shortest-path longevity network (SPLN) contains 171 genes/proteins. The Binding SPLN contains 33 of the genes/proteins previously reported to increase replicative life span that were used to build the Binding SPLN (Input), 45 genes that are required for vegetative growth in yeast (Essential), 5 non-essential genes that are not present in the *MAT*α haploid deletion collection or for which no viable cells were obtained (No Grow/No Deletion), and 88 genes for which replicative life span was assayed (RLS Determined). Seven of the genes/proteins previously reported to increase replicative life span are present in the Composite SPLN (**Figure S1**), but are not contained in the Binding SPLN: Gpr1, Hxk2, Rpd3, Rpl31a, Sch9, Uth1, and Ybr266c.

ORF	Gene	Status
YCR088W	ABP1	RLS Determined
YFL039C	ACT1	Essential
YDR448W	ADA2	RLS Determined
YDR216W	ADR1	RLS Determined
YCL025C	AGP1	RLS Determined
YKL135C	APL2	RLS Determined
YDL029W	ARP2	Essential
YJR065C	ARP3	Essential
YPR034W	ARP7	Essential
YDR101C	ARX1	RLS Determined
YNL101W	AVT4	RLS Determined
YER177W	BMH1	RLS Determined
YDR099W	BMH2	RLS Determined
YIL159W	BNR1	RLS Determined
YBL085W	BOI1	RLS Determined
YER114C	BOI2	RLS Determined
YNR051C	BRE5	Input
YLR310C	CDC25	Input
YBR160W	CDC28	Essential
YJL194W	CDC6	Input
YDR301W	CFT1	Essential
YHR142W	CHS7	RLS Determined
YHR052W	CIC1	Essential
YNL298W	CLA4	RLS Determined
YPR119W	CLB2	RLS Determined
YBR036C	CSG2	RLS Determined
YJR048W	CYC1	RLS Determined
YJL005W	CYR1	Input
YGR155W	CYS4	RLS Determined
YKL087C	CYT2	RLS Determined
YHR169W	DBP8	Essential

YDR385W	EFT2	RLS Determined
YOR144C	ELG1	RLS Determined
YPL101W	ELP4	RLS Determined
YKL182W	FAS1	Essential
YPR104C	FHL1	Essential
YLR342W	FKS1	RLS Determined
YDR110W	FOB1	Input
YER027C	GAL83	RLS Determined
YMR307W	GAS1	RLS Determined
YDR283C	GCN2	RLS Determined
YEL009C	GCN4	RLS Determined
YKL104C	GFA1	Essential
YML094W	GIM5	RLS Determined
YDR507C	GIN4	RLS Determined
YCL040W	GLK1	RLS Determined
YER020W	GPA2	Input
YDR331W	GPI8	Essential
YKL109W	HAP4	Input
YJL033W	HCA4	Essential
YGL194C	HOS2	RLS Determined
YPL240C	HSP82	RLS Determined
YOL012C	HTZ1	RLS Determined
YDR342C	HXT7	No Deletion/No Grow
YEL034W	HYP2	Essential
YOR136W	IDH2	Input
YNL009W	IDP3	RLS Determined
YLR095C	IOC2	RLS Determined
YJR091C	JSN1	RLS Determined
YER110C	KAP123	RLS Determined
YHL003C	LAG1	Input
YOL025W	LAG2	Input
YFR001W	LOC1	RLS Determined
YDR060W	MAK21	Essential
YLR163C	MAS1	Essential
YBR084W	MIS1	RLS Determined
YIL106W	MOB1	Essential
YGL178W	MPT5	Input
YPR030W	MRG19	Input
YNL124W	NAF1	Essential
YKR048C	NAP1	RLS Determined
YMR309C	NIP1	Essential
YHR170W	NMD3	Essential
YLR285W	NNT1	Input
YNR053C	NOG2	Essential
YNL110C	NOP15	Essential
YNL061W	NOP2	Essential

YPL043W	NOP4	Essential
YOR209C	NPT1	Input
YDR288W	NSE3	Essential
YDR001C	NTH1	RLS Determined
YMR047C	NUP116	Essential
YBR060C	ORC2	Essential
YLR350W	ORM2	RLS Determined
YGR087C	PDC6	RLS Determined
YKL043W	PHD1	RLS Determined
YGL008C	PMA1	Essential
YGL037C	PNC1	Input
YDR473C	PRP3	Essential
YER095W	RAD51	RLS Determined
YPL153C	RAD53	Essential
YNL216W	RAP1	Essential
YOR101W	RAS1	Input
YNL0986	RAS2	Input
YDR195W	REF2	RLS Determined
YDR028C	REG1	RLS Determined
YBR267W	REI1	Input
YFR051C	RET2	Essential
YCR028CA	RIM1	RLS Determined
YMR061W	RNA14	Essential
YLR371W	ROM2	Input
YPR190C	RPC82	Essential
YMR242C	RPL20A	RLS Determined
YOR312C	RPL20B	RLS Determined
YLR406C	RPL31B	RLS Determined
YLR448W	RPL6B	Input
YHR200W	RPN10	RLS Determined
YBL103C	RTG3	Input
YDR233C	RTN1	RLS Determined
YCR009C	RVS161	RLS Determined
YDR388W	RVS167	No Deletion/No Grow
YDR129C	SAC6	RLS Determined
YOR367W	SCP1	Input
YER120W	SCS2	RLS Determined
YGR245C	SDA1	Essential
YGL137W	SEC27	Essential
YIL076W	SEC28	RLS Determined
YDL195W	SEC31	Essential
YHR098C	SFB3	No Deletion/No Grow
YPR161C	SGV1	Essential
YGL208W	SIP2	RLS Determined
YJL089W	SIP4	RLS Determined
YDL042C	SIR2	Input

YDR227W	SIR4	Input
YHR149C	SKG6	No Deletion/No Grow
YBL007C	SLA1	RLS Determined
YGR229C	SMI1	RLS Determined
YDR510W	SMT3	Essential
YDR477W	SNF1	RLS Determined
YOR290C	SNF2	RLS Determined
YGL115W	SNF4	Input
YBR289W	SNF5	RLS Determined
YDR006C	SOK1	RLS Determined
YJR010CA	SPC1	RLS Determined
YDR523C	SPS1	RLS Determined
YML010W	SPT5	Essential
YDR293C	SSD1	Input
YPL042C	SSN3	RLS Determined
YPR163C	STM1	RLS Determined
YBR231C	SWC5	RLS Determined
YJL187C	SWE1	RLS Determined
YPL016W	SWI1	Essential
YDR146C	SWI5	RLS Determined
YMR149W	SWP1	Essential
YDR334W	SWR1	RLS Determined
YPL129W	TAF14	RLS Determined
YGR162W	TIF4631	RLS Determined
YPR016C	TIF6	Essential
YKL056C	TMA19	RLS Determined
YDR457W	TOM1	RLS Determined
YOL006C	TOP1	RLS Determined
YJR066W	TOR1	Input
YPL203W	TPK2	Input
YHR099W	TRA1	Essential
YCR084C	TUP1	RLS Determined
YER151C	UBP3	RLS Determined
YDR207C	UME6	RLS Determined
YNL229C	URE2	Input
YML041C	VPS71	RLS Determined
YOR043W	WHI2	RLS Determined
YNL107W	YAF9	RLS Determined
YAR010C	YAR010C	No Deletion/No Grow
YBR238C	YBR238C	Input
YBR255W	YBR255W	Input
YGR234W	YHB1	RLS Determined
YLR200W	YKE2	RLS Determined
YOR135C	YOR135C	Input
YBR183W	YPC1	RLS Determined
YDR381W	YRA1	Essential

YHR016C	YSC84	RLS Determined
YMR273C	ZDS1	Input
