# Supplementary material for: Shortest-Path Network Analysis Is a Useful Approach toward Identifying Genetic Determinants of Longevity
Source: PLoS One. 2008 Nov 25;3(11):e3802. doi: 10.1371/journal.pone.0003802 (PMC2583956; doi:10.1371/journal.pone.0003802)
Supplement: Table S4 — Replicative life span analysis of single-gene deletion strains corresponding to genes in the binding shortest-path longevity network. (0.14 MB PDF) [file pone.0003802.s006.pdf]

Table S4. Replicative life span analysis of single-gene deletion strains corresponding to genes in the binding shortest-path longevity network. Replicative life span was determined for each single gene deletion strain (Δ) present in the *MATα* ORF deletion collection that corresponds to a gene in the shortest-path longevity network (SPLN). Replicative life span data for each haploid deletion (Δ) and experiment matched wild type mother cells are shown as mean replicative life span with number of cells assayed in parentheses. *P*-value is calculated by a Wilcoxon Rank-Sum test. Pooled haploid data refers to pooled *MATα* and *MAT***a** deletion and wild type data.

		MATα			***MAT*a**			**Pooled haploid mating types**		
**ORF**	**GENE**	**Δ**	**BY4742**	**p-value**	**Δ**	**BY4741**	**p-value**	**Δ**	**WT Pooled**	**p-value**
YCR088W	ABP1	30.5 (40)	25.4 (45)	1.1E-02	25.5 (80)	30.4 (100)	4.0E-04	27.2 (120)	28.8 (145)	1.4E-01
YDR448W	ADA2	11.6 (5)	24.2 (5)	7.9E-03	0 (0)	0 (0)	0.0E+00	11.6 (5)	24.2 (5)	7.9E-03
YDR216W	ADR1	17.2 (5)	25.6 (5)	7.9E-03	0 (0)	0 (0)	0.0E+00	17.2 (5)	25.6 (5)	7.9E-03
YCL025C	AGP1	19.4 (5)	22.8 (5)	2.9E-01	0 (0)	0 (0)	0.0E+00	19.4 (5)	22.8 (5)	2.9E-01
YKL135C	APL2	18.8 (5)	25.6 (5)	4.0E-02	0 (0)	0 (0)	0.0E+00	18.8 (5)	25.6 (5)	4.0E-02
YDR101C	ARX1	24.4 (25)	24.5 (35)	7.6E-01	0 (0)	0 (0)	0.0E+00	24.4 (25)	24.5 (35)	7.6E-01
YNL101W	AVT4	19.2 (5)	36.4 (5)	1.0E-01	0 (0)	0 (0)	0.0E+00	19.2 (5)	36.4 (5)	1.0E-01
YER177W	BMH1	20.1 (10)	22.7 (10)	6.8E-01	0 (0)	0 (0)	0.0E+00	20.1 (10)	22.7 (10)	6.8E-01
YDR099W	BMH2	26.1 (15)	28.5 (15)	3.0E-01	0 (0)	0 (0)	0.0E+00	26.1 (15)	28.5 (15)	3.0E-01
YIL159W	BNR1	25 (5)	23.6 (5)	1.0E+00	0 (0)	0 (0)	0.0E+00	25 (5)	23.6 (5)	1.0E+00
YBL085W	BOI1	25.3 (25)	26.4 (25)	4.2E-01	0 (0)	0 (0)	0.0E+00	25.3 (25)	26.4 (25)	4.2E-01
YER114C	BOI2	35.2 (45)	25.8 (65)	1.0E-05	30.1 (80)	28.7 (100)	3.8E-01	31.9 (125)	27.6 (165)	4.5E-04
YHR142W	CHS7	18 (5)	22.4 (5)	4.6E-01	0 (0)	0 (0)	0.0E+00	18 (5)	22.4 (5)	4.6E-01
YNL298W	CLA4	10 (25)	23.8 (45)	2.6E-09	0 (0)	0 (0)	0.0E+00	10 (25)	23.8 (45)	2.6E-09
YPR119W	CLB2	28.7 (65)	24.6 (65)	2.2E-02	30.9 (40)	30 (80)	9.4E-01	29.5 (105)	27.6 (145)	3.3E-01
YBR036C	CSG2	18.8 (5)	29.4 (5)	2.4E-01	0 (0)	0 (0)	0.0E+00	18.8 (5)	29.4 (5)	2.4E-01
YJR048W	CYC1	13.2 (5)	31.4 (5)	7.9E-03	0 (0)	0 (0)	0.0E+00	13.2 (5)	31.4 (5)	7.9E-03
YGR155W	CYS4	14.4 (5)	20.8 (5)	2.9E-01	0 (0)	0 (0)	0.0E+00	14.4 (5)	20.8 (5)	2.9E-01
YKL087C	CYT2	17.9 (46)	23.9 (65)	4.0E-03	0 (0)	0 (0)	0.0E+00	17.9 (46)	23.9 (65)	4.0E-03
YDR385W	EFT2	22.3 (15)	21.8 (25)	7.2E-01	0 (0)	0 (0)	0.0E+00	22.3 (15)	21.8 (25)	7.2E-01
YOR144C	ELG1	25 (5)	32.8 (5)	3.8E-01	0 (0)	0 (0)	0.0E+00	25 (5)	32.8 (5)	3.8E-01

YPL101W	ELP4	34.5 (45)	25.6 (65)	1.2E-05	31.3 (80)	27.9 (120)	9.0E-03	32.4 (125)	27.1 (185)	2.5E-06
YLR342W	FKS1	24 (5)	20.6 (5)	5.2E-01	0 (0)	0 (0)	0.0E+00	24 (5)	20.6 (5)	5.2E-01
YER027C	GAL83	13.2 (5)	20.8 (5)	4.2E-01	0 (0)	0 (0)	0.0E+00	13.2 (5)	20.8 (5)	4.2E-01
YMR307W	GAS1	18.6 (20)	24.1 (25)	8.2E-03	0 (0)	0 (0)	0.0E+00	18.6 (20)	24.1 (25)	8.2E-03
YDR283C	GCN2	25.7 (495)	27 (565)	2.8E-02	23.3 (180)	28.8 (220)	1.1E-08	25.1 (675)	27.5 (785)	8.5E-07
YEL009C	GCN4	26.9 (1165)	25.1 (1125)	1.3E-04	26.3 (240)	26.5 (320)	7.1E-01	26.8 (1405)	25.4 (1445)	1.6E-03
YML094W	GIM5	19 (5)	29.6 (5)	1.5E-01	0 (0)	0 (0)	0.0E+00	19 (5)	29.6 (5)	1.5E-01
YDR507C	GIN4	14.9 (25)	22.6 (25)	4.1E-04	0 (0)	0 (0)	0.0E+00	14.9 (25)	22.6 (25)	4.1E-04
YCL040W	GLK1	27.9 (65)	25.9 (105)	1.8E-01	24.7 (40)	28.3 (40)	1.7E-02	26.7 (105)	26.5 (145)	8.5E-01
YGL194C	HOS2	27.8 (65)	25.8 (105)	1.5E-01	0 (0)	0 (0)	0.0E+00	27.8 (65)	25.8 (105)	1.5E-01
YPL240C	HSP82	27.7 (25)	26.3 (45)	6.5E-01	0 (0)	0 (0)	0.0E+00	27.7 (25)	26.3 (45)	6.5E-01
YOL012C	HTZ1	19.2 (5)	30 (5)	1.5E-01	0 (0)	0 (0)	0.0E+00	19.2 (5)	30 (5)	1.5E-01
YNL009W	IDP3	29.9 (65)	26.2 (65)	2.2E-02	23.5 (80)	28 (100)	3.8E-04	26.3 (145)	27.3 (165)	2.6E-01
YLR095C	IOC2	23.7 (25)	27.9 (25)	9.8E-02	0 (0)	0 (0)	0.0E+00	23.7 (25)	27.9 (25)	9.8E-02
YJR091C	JSN1	22.4 (5)	28.4 (5)	3.3E-01	0 (0)	0 (0)	0.0E+00	22.4 (5)	28.4 (5)	3.3E-01
YER110C	KAP123	22.8 (5)	20.4 (5)	5.2E-01	0 (0)	0 (0)	0.0E+00	22.8 (5)	20.4 (5)	5.2E-01
YFR001W	LOC1	36.7 (45)	25.9 (45)	9.3E-05	0 (0)	0 (0)	0.0E+00	36.7 (45)	25.9 (45)	9.3E-05
YBR084W	MIS1	25.6 (5)	28.6 (5)	8.4E-01	0 (0)	0 (0)	0.0E+00	25.6 (5)	28.6 (5)	8.4E-01
YKR048C	NAP1	24.6 (45)	27.2 (45)	6.3E-02	0 (0)	0 (0)	0.0E+00	24.6 (45)	27.2 (45)	6.3E-02
YDR001C	NTH1	29.6 (65)	26.1 (105)	5.8E-02	25.3 (60)	29.5 (80)	1.5E-02	27.6 (125)	27.5 (185)	7.7E-01
YLR350W	ORM2	22.6 (10)	22.7 (10)	7.0E-01	0 (0)	0 (0)	0.0E+00	22.6 (10)	22.7 (10)	7.0E-01
YGR087C	PDC6	24.5 (25)	24.2 (45)	7.6E-01	0 (0)	0 (0)	0.0E+00	24.5 (25)	24.2 (45)	7.6E-01
YKL043W	PHD1	29 (25)	23.8 (25)	1.6E-02	27.5 (40)	26.2 (80)	4.8E-01	28 (65)	25.6 (105)	7.4E-02
YER095W	RAD51	8.2 (5)	22.2 (5)	7.9E-02	0 (0)	0 (0)	0.0E+00	8.2 (5)	22.2 (5)	7.9E-02
YDR195W	REF2	18.8 (5)	25.6 (5)	1.5E-01	0 (0)	0 (0)	0.0E+00	18.8 (5)	25.6 (5)	1.5E-01
YDR028C	REG1	19.2 (5)	26.4 (5)	9.5E-02	0 (0)	0 (0)	0.0E+00	19.2 (5)	26.4 (5)	9.5E-02
YCR028CA	RIM1	29 (65)	25.5 (85)	2.4E-02	33.9 (100)	28.6 (160)	3.1E-04	32 (165)	27.5 (245)	4.9E-05
YMR242C	RPL20A	15.7 (55)	26.2 (65)	3.0E-09	21.6 (20)	21.3 (20)	8.5E-01	17.3 (75)	25 (85)	6.4E-08
YOR312C	RPL20B	36 (345)	26.4 (345)	4.8E-30	37.2 (100)	25.3 (80)	2.1E-10	36.3 (445)	26.2 (425)	4.6E-39
YLR406C	RPL31B	22.3 (45)	24.2 (45)	3.5E-01	0 (0)	0 (0)	0.0E+00	22.3 (45)	24.2 (45)	3.5E-01
YHR200W	RPN10	18.8 (5)	22.8 (5)	5.4E-01	0 (0)	0 (0)	0.0E+00	18.8 (5)	22.8 (5)	5.4E-01

YDR233C	RTN1	29.8 (65)	25.8 (85)	9.2E-03	27.8 (80)	30.1 (120)	1.9E-01	28.7 (145)	28.3 (205)	5.8E-01
YCR009C	RVS161	29.4 (45)	25.9 (65)	2.5E-02	13.2 (40)	29.7 (80)	4.8E-12	21.8 (85)	28 (145)	1.2E-04
YDR129C	SAC6	14.7 (30)	25.8 (60)	2.4E-05	0 (0)	0 (0)	0.0E+00	14.7 (30)	25.8 (60)	2.4E-05
YER120W	SCS2	16.6 (5)	20.4 (5)	6.3E-01	0 (0)	0 (0)	0.0E+00	16.6 (5)	20.4 (5)	6.3E-01
YIL076W	SEC28	31.2 (35)	25.6 (65)	5.7E-03	29.7 (60)	31 (80)	5.2E-01	30.3 (95)	28.6 (145)	2.3E-01
YGL208W	SIP2	31.1 (125)	26.4 (145)	2.6E-04	30.8 (40)	28.3 (40)	2.1E-01	31 (165)	26.8 (185)	2.1E-04
YJL089W	SIP4	17.4 (5)	18.8 (5)	8.4E-01	0 (0)	0 (0)	0.0E+00	17.4 (5)	18.8 (5)	8.4E-01
YBL007C	SLA1	8.6 (25)	21.9 (25)	3.2E-08	0 (0)	0 (0)	0.0E+00	8.6 (25)	21.9 (25)	3.2E-08
YGR229C	SMI1	22.8 (5)	21.8 (5)	5.2E-01	0 (0)	0 (0)	0.0E+00	22.8 (5)	21.8 (5)	5.2E-01
YDR477W	SNF1	38.8 (40)	25.5 (40)	2.4E-06	26.1 (100)	27 (120)	3.5E-01	29.8 (140)	26.6 (160)	4.9E-02
YOR290C	SNF2	24.8 (5)	28.6 (5)	5.7E-01	0 (0)	0 (0)	0.0E+00	24.8 (5)	28.6 (5)	5.7E-01
YBR289W	SNF5	27.8 (25)	25.4 (45)	2.3E-01	0 (0)	0 (0)	0.0E+00	27.8 (25)	25.4 (45)	2.3E-01
YDR006C	SOK1	39.8 (85)	25.9 (105)	3.4E-14	35 (100)	28.8 (120)	1.8E-05	37.2 (185)	27.4 (225)	1.4E-17
YJR010CA	SPC1	27.8 (25)	27.6 (25)	1.0E+00	0 (0)	0 (0)	0.0E+00	27.8 (25)	27.6 (25)	1.0E+00
YDR523C	SPS1	31.5 (45)	25.4 (45)	9.2E-03	33.9 (80)	29.8 (80)	3.8E-02	33 (125)	28.2 (125)	2.2E-03
YPL042C	SSN3	21.2 (55)	26.6 (105)	1.1E-04	0 (0)	0 (0)	0.0E+00	21.2 (55)	26.6 (105)	1.1E-04
YPR163C	STM1	17.9 (25)	22.8 (25)	1.6E-01	0 (0)	0 (0)	0.0E+00	17.9 (25)	22.8 (25)	1.6E-01
YBR231C	SWC5	15.9 (10)	29.6 (10)	1.7E-03	0 (0)	0 (0)	0.0E+00	15.9 (10)	29.6 (10)	1.7E-03
YJL187C	SWE1	15.4 (5)	22 (5)	2.0E-01	0 (0)	0 (0)	0.0E+00	15.4 (5)	22 (5)	2.0E-01
YDR146C	SWI5	31 (45)	25.7 (85)	1.2E-02	0 (0)	0 (0)	0.0E+00	31 (45)	25.7 (85)	1.2E-02
YDR334W	SWR1	24.1 (30)	26.9 (30)	1.1E-01	0 (0)	0 (0)	0.0E+00	24.1 (30)	26.9 (30)	1.1E-01
YPL129W	TAF14	29.6 (65)	25.7 (105)	1.2E-02	14.9 (60)	31 (80)	2.7E-12	22.6 (125)	28 (185)	1.3E-04
YGR162W	TIF4631	27.9 (95)	23.7 (145)	6.6E-03	33.1 (100)	27 (100)	2.8E-04	30.6 (195)	25 (245)	4.3E-07
YKL056C	TMA19	33.4 (45)	25.3 (65)	2.2E-04	37.4 (60)	30 (80)	1.6E-03	35.7 (105)	27.9 (145)	2.0E-06
YDR457W	TOM1	30.1 (45)	25.4 (65)	2.1E-02	31.4 (100)	28.6 (160)	8.8E-02	31 (145)	27.7 (225)	8.7E-03
YOL006C	TOP1	12.4 (5)	30 (5)	7.9E-03	0 (0)	0 (0)	0.0E+00	12.4 (5)	30 (5)	7.9E-03
YCR084C	TUP1	23.8 (20)	24.2 (25)	8.4E-01	0 (0)	0 (0)	0.0E+00	23.8 (20)	24.2 (25)	8.4E-01
YER151C	UBP3	27.7 (65)	25.1 (105)	2.2E-01	0 (0)	0 (0)	0.0E+00	27.7 (65)	25.1 (105)	2.2E-01
YDR207C	UME6	14.6 (5)	25.6 (5)	7.9E-03	0 (0)	0 (0)	0.0E+00	14.6 (5)	25.6 (5)	7.9E-03
YML041C	VPS71	12.6 (5)	20.8 (5)	1.5E-01	0 (0)	0 (0)	0.0E+00	12.6 (5)	20.8 (5)	1.5E-01
YOR043W	WHI2	13.6 (5)	24 (5)	7.9E-03	0 (0)	0 (0)	0.0E+00	13.6 (5)	24 (5)	7.9E-03

YNL107W	YAF9	27.8 (25)	26 (25)	7.4E-01	0 (0)	0 (0)	0.0E+00	27.8 (25)	26 (25)	7.4E-01
YGR234W	YHB1	21.8 (5)	21.8 (5)	7.6E-01	0 (0)	0 (0)	0.0E+00	21.8 (5)	21.8 (5)	7.6E-01
YLR200W	YKE2	24.5 (25)	25.4 (25)	7.0E-01	0 (0)	0 (0)	0.0E+00	24.5 (25)	25.4 (25)	7.0E-01
YBR183W	YPC1	29 (45)	26 (85)	1.4E-01	24.1 (40)	27.9 (40)	4.0E-02	26.7 (85)	26.6 (125)	8.8E-01
YHR016C	YSC84	28.7 (40)	24.1 (65)	1.9E-02	27.9 (100)	28.9 (120)	3.3E-01	28.1 (140)	27.2 (185)	5.1E-01
