# Supplementary material for: Shortest-Path Network Analysis Is a Useful Approach toward Identifying Genetic Determinants of Longevity
Source: PLoS One. 2008 Nov 25;3(11):e3802. doi: 10.1371/journal.pone.0003802 (PMC2583956; doi:10.1371/journal.pone.0003802)
Supplement: Table S5 — Replicative life span analysis of 564 randomly selected single-gene deletion strains. (0.36 MB PDF) [file pone.0003802.s007.pdf]

**Table S5. Replicative life span analysis of 564 randomly selected single-gene deletion strains**. Preliminary replicative life span (RLS) analysis of these 564 single-gene deletion strains was previously described [1]. The data presented here include the previously reported RLS data for these strains and additional RLS data for these strains obtained since publication of the prior data set as part of an ongoing genome-wide study of RLS across the entire yeast ORF deletion collection [2]. RLS for each haploid deletion (Δ) and experiment matched wild type mother cells are shown as mean replicative life span with number of cells assayed in parentheses. *P*-value is calculated by a Wilcoxon Rank-Sum test. Pooled haploid data refers to pooled *MATα* and *MAT***a** deletion and wild type data.

| | | *MATα* | | | *MAT*a | | | Pooled haploid mating types | | |
|---|---|---|---|---|---|---|---|---|---|---|
| ORF | GENE | Δ | BY4742 | p-value | Δ | BY4741 | p-value | Δ | WT Pooled | p-value |
| YMR056C | AAC1 | 25.3 (105) | 23.9 (135) | 2.2E-01 | 28.7 (100) | 26.8 (120) | 1.3E-01 | 26.9 (205) | 25.3 (255) | 2.7E-02 |
| YCR107W | AAD3 | 27.4 (20) | 23.8 (20) | 3.7E-01 | 0 (0) | 0 (0) | 0.0E+00 | 27.4 (20) | 23.8 (20) | 3.7E-01 |
| YHR047C | AAP1 | 15.8 (5) | 22.4 (5) | 1.9E-01 | 0 (0) | 0 (0) | 0.0E+00 | 15.8 (5) | 22.4 (5) | 1.9E-01 |
| YAL054C | ACS1 | 24.5 (10) | 26.1 (10) | 5.7E-01 | 0 (0) | 0 (0) | 0.0E+00 | 24.5 (10) | 26.1 (10) | 5.7E-01 |
| YGR061C | ADE6 | 25.7 (25) | 28.4 (25) | 4.0E-01 | 0 (0) | 0 (0) | 0.0E+00 | 25.7 (25) | 28.4 (25) | 4.0E-01 |
| YER170W | ADK2 | 23.4 (10) | 22.7 (10) | 6.5E-01 | 0 (0) | 0 (0) | 0.0E+00 | 23.4 (10) | 22.7 (10) | 6.5E-01 |
| YDR085C | AFR1 | 23.4 (10) | 29.6 (10) | 8.1E-02 | 0 (0) | 0 (0) | 0.0E+00 | 23.4 (10) | 29.6 (10) | 8.1E-02 |
| YHR093W | AHT1 | 28.4 (15) | 25 (25) | 5.4E-01 | 0 (0) | 0 (0) | 0.0E+00 | 28.4 (15) | 25 (25) | 5.4E-01 |
| YDR264C | AKR1 | 14.7 (25) | 25.8 (25) | 9.5E-06 | 0 (0) | 0 (0) | 0.0E+00 | 14.7 (25) | 25.8 (25) | 9.5E-06 |
| YMR169C | ALD3 | 29.4 (10) | 30 (10) | 9.1E-01 | 0 (0) | 0 (0) | 0.0E+00 | 29.4 (10) | 30 (10) | 9.1E-01 |
| YDR111C | ALT2 | 19.7 (10) | 29.6 (10) | 8.2E-02 | 0 (0) | 0 (0) | 0.0E+00 | 19.7 (10) | 29.6 (10) | 8.2E-02 |
| YDR242W | AMD2 | 23.8 (30) | 25.4 (30) | 2.8E-01 | 0 (0) | 0 (0) | 0.0E+00 | 23.8 (30) | 25.4 (30) | 2.8E-01 |
| YBR231C | AOR1 | 15.9 (10) | 29.6 (10) | 1.7E-03 | 0 (0) | 0 (0) | 0.0E+00 | 15.9 (10) | 29.6 (10) | 1.7E-03 |
| YHR018C | ARG4 | 29 (25) | 26.3 (25) | 5.4E-01 | 0 (0) | 0 (0) | 0.0E+00 | 29 (25) | 26.3 (25) | 5.4E-01 |
| YBR249C | ARO4 | 26.5 (15) | 28.5 (15) | 9.7E-01 | 0 (0) | 0 (0) | 0.0E+00 | 26.5 (15) | 28.5 (15) | 9.7E-01 |
| YHR137W | ARO9 | 20 (5) | 22.4 (5) | 8.4E-01 | 0 (0) | 0 (0) | 0.0E+00 | 20 (5) | 22.4 (5) | 8.4E-01 |
| YHR129C | ARP1 | 15.7 (20) | 24.5 (25) | 3.3E-03 | 0 (0) | 0 (0) | 0.0E+00 | 15.7 (20) | 24.5 (25) | 3.3E-03 |
| YDR101C | ARX1 | 24.4 (25) | 24.5 (35) | 7.6E-01 | 0 (0) | 0 (0) | 0.0E+00 | 24.4 (25) | 24.5 (35) | 7.6E-01 |
| YGR097W | ASK10 | 23.7 (10) | 29.3 (10) | 3.1E-01 | 0 (0) | 0 (0) | 0.0E+00 | 23.7 (10) | 29.3 (10) | 3.1E-01 |

| | | | | | | | | | | |
|---|---|---|---|---|---|---|---|---|---|---|
| YDR321W | ASP1 | 31.5 (20) | 26.9 (35) | 3.5E-01 | 0 (0) | 0 (0) | 0.0E+00 | 31.5 (20) | 26.9 (35) | 3.5E-01 |
| YLR423C | ATG17 | 18.4 (5) | 28.8 (5) | 2.9E-01 | 0 (0) | 0 (0) | 0.0E+00 | 18.4 (5) | 28.8 (5) | 2.9E-01 |
| YCL038C | ATG22 | 25.7 (10) | 28.5 (10) | 6.5E-01 | 0 (0) | 0 (0) | 0.0E+00 | 25.7 (10) | 28.5 (10) | 6.5E-01 |
| YLR431C | ATG23 | 25.4 (20) | 26.9 (30) | 3.4E-01 | 0 (0) | 0 (0) | 0.0E+00 | 25.4 (20) | 26.9 (30) | 3.4E-01 |
| YML081CA | ATP18 | 15.5 (14) | 24.5 (45) | 1.1E-03 | 0 (0) | 0 (0) | 0.0E+00 | 15.5 (14) | 24.5 (45) | 1.1E-03 |
| YDR298C | ATP5 | 23.4 (25) | 25.8 (25) | 2.6E-01 | 0 (0) | 0 (0) | 0.0E+00 | 23.4 (25) | 25.8 (25) | 2.6E-01 |
| YOR113W | AZF1 | 28 (45) | 26 (45) | 3.3E-01 | 0 (0) | 0 (0) | 0.0E+00 | 28 (45) | 26 (45) | 3.3E-01 |
| YOR134W | BAG7 | 30.3 (20) | 25.4 (65) | 1.6E-01 | 22.9 (20) | 22.1 (20) | 6.4E-01 | 26.6 (40) | 24.6 (85) | 5.7E-01 |
| YKR099W | BAS1 | 35.6 (30) | 26.9 (30) | 1.7E-03 | 23.1 (40) | 25.3 (60) | 1.8E-01 | 28.5 (70) | 25.8 (90) | 1.9E-01 |
| YNR058W | BIO3 | 29.9 (20) | 24.8 (50) | 1.3E-01 | 0 (0) | 0 (0) | 0.0E+00 | 29.9 (20) | 24.8 (50) | 1.3E-01 |
| YNR057C | BIO4 | 21.8 (5) | 28.8 (5) | 4.5E-01 | 0 (0) | 0 (0) | 0.0E+00 | 21.8 (5) | 28.8 (5) | 4.5E-01 |
| YNR056C | BIO5 | 20.8 (5) | 28.8 (5) | 3.8E-01 | 0 (0) | 0 (0) | 0.0E+00 | 20.8 (5) | 28.8 (5) | 3.8E-01 |
| YER177W | BMH1 | 20.1 (10) | 22.7 (10) | 6.8E-01 | 0 (0) | 0 (0) | 0.0E+00 | 20.1 (10) | 22.7 (10) | 6.8E-01 |
| YDR099W | BMH2 | 26.1 (15) | 28.5 (15) | 3.0E-01 | 0 (0) | 0 (0) | 0.0E+00 | 26.1 (15) | 28.5 (15) | 3.0E-01 |
| YNR051C | BRE5 | 25.8 (80) | 26.6 (130) | 4.8E-01 | 32.8 (40) | 25.3 (60) | 6.6E-03 | 28.2 (120) | 26.1 (190) | 2.8E-01 |
| YDR275W | BSC2 | 34.2 (25) | 27.6 (25) | 3.4E-02 | 28.2 (20) | 29.7 (20) | 9.0E-01 | 31.6 (45) | 28.5 (45) | 2.0E-01 |
| YDR252W | BTT1 | 24.8 (5) | 29 (5) | 7.3E-01 | 0 (0) | 0 (0) | 0.0E+00 | 24.8 (5) | 29 (5) | 7.3E-01 |
| YMR055C | BUB2 | 22.6 (15) | 23.7 (15) | 4.8E-01 | 0 (0) | 0 (0) | 0.0E+00 | 22.6 (15) | 23.7 (15) | 4.8E-01 |
| YLR353W | BUD8 | 31.3 (25) | 24 (55) | 5.2E-02 | 0 (0) | 0 (0) | 0.0E+00 | 31.3 (25) | 24 (55) | 5.2E-02 |
| YHR114W | BZZ1 | 20.8 (5) | 22.4 (5) | 8.0E-01 | 0 (0) | 0 (0) | 0.0E+00 | 20.8 (5) | 22.4 (5) | 8.0E-01 |
| YLR438W | CAR2 | 16.4 (5) | 28.8 (5) | 5.6E-02 | 0 (0) | 0 (0) | 0.0E+00 | 16.4 (5) | 28.8 (5) | 5.6E-02 |
| YDR270W | CCC2 | 17.8 (5) | 29 (5) | 2.2E-01 | 0 (0) | 0 (0) | 0.0E+00 | 17.8 (5) | 29 (5) | 2.2E-01 |
| YDR254W | CHL4 | 22.6 (10) | 27.4 (10) | 2.7E-01 | 0 (0) | 0 (0) | 0.0E+00 | 22.6 (10) | 27.4 (10) | 2.7E-01 |
| YHR142W | CHS7 | 18 (5) | 22.4 (5) | 4.6E-01 | 0 (0) | 0 (0) | 0.0E+00 | 18 (5) | 22.4 (5) | 4.6E-01 |
| YGR108W | CLB1 | 24 (25) | 28.4 (25) | 1.5E-02 | 0 (0) | 0 (0) | 0.0E+00 | 24 (25) | 28.4 (25) | 1.5E-02 |
| YGR109C | CLB6 | 24.1 (25) | 28.4 (25) | 2.0E-01 | 0 (0) | 0 (0) | 0.0E+00 | 24.1 (25) | 28.4 (25) | 2.0E-01 |
| YLR433C | CNA1 | 23.8 (20) | 26.9 (30) | 1.9E-01 | 0 (0) | 0 (0) | 0.0E+00 | 23.8 (20) | 26.9 (30) | 1.9E-01 |
| YLL018CA | COX19 | 17.4 (5) | 28.8 (5) | 1.0E-01 | 0 (0) | 0 (0) | 0.0E+00 | 17.4 (5) | 28.8 (5) | 1.0E-01 |
| YIL111W | COX5B | 27.8 (20) | 24.4 (20) | 2.6E-01 | 0 (0) | 0 (0) | 0.0E+00 | 27.8 (20) | 24.4 (20) | 2.6E-01 |
| YHR057C | CPR2 | 24 (5) | 22.4 (5) | 4.4E-01 | 0 (0) | 0 (0) | 0.0E+00 | 24 (5) | 22.4 (5) | 4.4E-01 |
| YDR304C | CPR5 | 25.6 (5) | 29 (5) | 7.9E-01 | 0 (0) | 0 (0) | 0.0E+00 | 25.6 (5) | 29 (5) | 7.9E-01 |

| | | | | | | | | | | |
|---|---|---|---|---|---|---|---|---|---|---|
| YOR100C | CRC1 | 31.6 (25) | 25.3 (25) | 1.7E-02 | 25.9 (40) | 25.3 (60) | 6.7E-01 | 28.1 (65) | 25.3 (85) | 2.5E-01 |
| YLR429W | CRN1 | 30.2 (25) | 27.6 (25) | 5.6E-01 | 0 (0) | 0 (0) | 0.0E+00 | 30.2 (25) | 27.6 (25) | 5.6E-01 |
| YLR380W | CSR1 | 22.3 (15) | 23.7 (15) | 5.5E-01 | 0 (0) | 0 (0) | 0.0E+00 | 22.3 (15) | 23.7 (15) | 5.5E-01 |
| YDR256C | CTA1 | 29.9 (20) | 26.9 (35) | 4.3E-01 | 0 (0) | 0 (0) | 0.0E+00 | 29.9 (20) | 26.9 (35) | 4.3E-01 |
| YLR381W | CTF3 | 24.3 (10) | 22.7 (10) | 4.3E-01 | 0 (0) | 0 (0) | 0.0E+00 | 24.3 (10) | 22.7 (10) | 4.3E-01 |
| YHR109W | CTM1 | 22 (5) | 22.4 (5) | 1.0E+00 | 0 (0) | 0 (0) | 0.0E+00 | 22 (5) | 22.4 (5) | 1.0E+00 |
| YGR088W | CTT1 | 24.8 (25) | 28.4 (25) | 2.2E-01 | 0 (0) | 0 (0) | 0.0E+00 | 24.8 (25) | 28.4 (25) | 2.2E-01 |
| YAL012W | CYS3 | 30.2 (25) | 24.5 (25) | 1.9E-01 | 0 (0) | 0 (0) | 0.0E+00 | 30.2 (25) | 24.5 (25) | 1.9E-01 |
| YHR028C | DAP2 | 22 (5) | 22.4 (5) | 8.8E-01 | 0 (0) | 0 (0) | 0.0E+00 | 22 (5) | 22.4 (5) | 8.8E-01 |
| YOR180C | DCI1 | 29.8 (25) | 28.4 (25) | 8.3E-01 | 0 (0) | 0 (0) | 0.0E+00 | 29.8 (25) | 28.4 (25) | 8.3E-01 |
| YOR173W | DCS2 | 22.8 (10) | 29.3 (10) | 2.1E-01 | 0 (0) | 0 (0) | 0.0E+00 | 22.8 (10) | 29.3 (10) | 2.1E-01 |
| YOR163W | DDP1 | 27.8 (25) | 25.3 (25) | 2.2E-01 | 0 (0) | 0 (0) | 0.0E+00 | 27.8 (25) | 25.3 (25) | 2.2E-01 |
| YFL001W | DEG1 | 27.8 (35) | 24.5 (35) | 1.9E-01 | 0 (0) | 0 (0) | 0.0E+00 | 27.8 (35) | 24.5 (35) | 1.9E-01 |
| YLR348C | DIC1 | 23.3 (15) | 23.7 (15) | 7.7E-01 | 0 (0) | 0 (0) | 0.0E+00 | 23.3 (15) | 23.7 (15) | 7.7E-01 |
| YDR263C | DIN7 | 22.6 (5) | 29 (5) | 5.8E-01 | 0 (0) | 0 (0) | 0.0E+00 | 22.6 (5) | 29 (5) | 5.8E-01 |
| YIR004W | DJP1 | 28.6 (25) | 24 (55) | 1.4E-01 | 0 (0) | 0 (0) | 0.0E+00 | 28.6 (25) | 24 (55) | 1.4E-01 |
| YER179W | DMC1 | 22.3 (15) | 23.7 (15) | 7.7E-01 | 0 (0) | 0 (0) | 0.0E+00 | 22.3 (15) | 23.7 (15) | 7.7E-01 |
| YDR093W | DNF2 | 22.9 (15) | 25.2 (20) | 4.3E-01 | 0 (0) | 0 (0) | 0.0E+00 | 22.9 (15) | 25.2 (20) | 4.3E-01 |
| YDR069C | DOA4 | 15.8 (10) | 29.6 (10) | 1.3E-03 | 0 (0) | 0 (0) | 0.0E+00 | 15.8 (10) | 29.6 (10) | 1.3E-03 |
| YHR044C | DOG1 | 25.8 (15) | 25 (25) | 9.1E-01 | 0 (0) | 0 (0) | 0.0E+00 | 25.8 (15) | 25 (25) | 9.1E-01 |
| YHR043C | DOG2 | 19.8 (5) | 22.4 (5) | 6.9E-01 | 0 (0) | 0 (0) | 0.0E+00 | 19.8 (5) | 22.4 (5) | 6.9E-01 |
| YDR273W | DON1 | 19.8 (5) | 29 (5) | 4.2E-01 | 0 (0) | 0 (0) | 0.0E+00 | 19.8 (5) | 29 (5) | 4.2E-01 |
| YDR068W | DOS2 | 27.4 (20) | 26.9 (30) | 8.0E-01 | 0 (0) | 0 (0) | 0.0E+00 | 27.4 (20) | 26.9 (30) | 8.0E-01 |
| YDR121W | DPB4 | 25.9 (10) | 29.6 (10) | 3.3E-01 | 0 (0) | 0 (0) | 0.0E+00 | 25.9 (10) | 29.6 (10) | 3.3E-01 |
| YDR294C | DPL1 | 23.4 (25) | 27.6 (25) | 2.0E-01 | 0 (0) | 0 (0) | 0.0E+00 | 23.4 (25) | 27.6 (25) | 2.0E-01 |
| YDR284C | DPP1 | 31.7 (20) | 25.9 (55) | 2.4E-01 | 0 (0) | 0 (0) | 0.0E+00 | 31.7 (20) | 25.9 (55) | 2.4E-01 |
| YHR143W | DSE2 | 15.4 (5) | 22.4 (5) | 9.5E-02 | 0 (0) | 0 (0) | 0.0E+00 | 15.4 (5) | 22.4 (5) | 9.5E-02 |
| YNR067C | DSE4 | 27.1 (20) | 26.9 (30) | 9.4E-01 | 0 (0) | 0 (0) | 0.0E+00 | 27.1 (20) | 26.9 (30) | 9.4E-01 |
| YKR054C | DYN1 | 20.1 (30) | 23.5 (50) | 1.2E-01 | 0 (0) | 0 (0) | 0.0E+00 | 20.1 (30) | 23.5 (50) | 1.2E-01 |
| YHR132C | ECM14 | 22.2 (5) | 22.4 (5) | 9.8E-01 | 0 (0) | 0 (0) | 0.0E+00 | 22.2 (5) | 22.4 (5) | 9.8E-01 |
| YDR125C | ECM18 | 26.2 (20) | 29.6 (20) | 4.9E-01 | 0 (0) | 0 (0) | 0.0E+00 | 26.2 (20) | 29.6 (20) | 4.9E-01 |

| | | | | | | | | | | |
|---|---|---|---|---|---|---|---|---|---|---|
| YLR390W | ECM19 | 23.2 (20) | 24.4 (20) | 6.5E-01 | 0 (0) | 0 (0) | 0.0E+00 | 23.2 (20) | 24.4 (20) | 6.5E-01 |
| YLR436C | ECM30 | 28.1 (20) | 26.9 (30) | 7.2E-01 | 0 (0) | 0 (0) | 0.0E+00 | 28.1 (20) | 26.9 (30) | 7.2E-01 |
| YER176W | ECM32 | 28.4 (25) | 25.4 (35) | 4.8E-01 | 0 (0) | 0 (0) | 0.0E+00 | 28.4 (25) | 25.4 (35) | 4.8E-01 |
| YLR443W | ECM7 | 13 (5) | 28.8 (5) | 9.5E-02 | 0 (0) | 0 (0) | 0.0E+00 | 13 (5) | 28.8 (5) | 9.5E-02 |
| YOR133W | EFT1 | 17.8 (15) | 23.8 (25) | 1.5E-02 | 0 (0) | 0 (0) | 0.0E+00 | 17.8 (15) | 23.8 (25) | 1.5E-02 |
| YOR144C | ELG1 | 25 (5) | 32.8 (5) | 3.8E-01 | 0 (0) | 0 (0) | 0.0E+00 | 25 (5) | 32.8 (5) | 3.8E-01 |
| YDR512C | EMI1 | 23.7 (30) | 25.3 (40) | 3.4E-01 | 0 (0) | 0 (0) | 0.0E+00 | 23.7 (30) | 25.3 (40) | 3.4E-01 |
| YIL005W | EPS1 | 24.4 (5) | 29 (5) | 6.9E-01 | 0 (0) | 0 (0) | 0.0E+00 | 24.4 (5) | 29 (5) | 6.9E-01 |
| YHR123W | EPT1 | 24.4 (15) | 25 (25) | 6.3E-01 | 0 (0) | 0 (0) | 0.0E+00 | 24.4 (15) | 25 (25) | 6.3E-01 |
| YHR110W | ERP5 | 15.6 (5) | 22.4 (5) | 2.5E-01 | 0 (0) | 0 (0) | 0.0E+00 | 15.6 (5) | 22.4 (5) | 2.5E-01 |
| YDR261C | EXG2 | 19.8 (5) | 29 (5) | 2.2E-01 | 0 (0) | 0 (0) | 0.0E+00 | 19.8 (5) | 29 (5) | 2.2E-01 |
| YMR052W | FAR3 | 26.8 (15) | 23.7 (15) | 6.3E-01 | 0 (0) | 0 (0) | 0.0E+00 | 26.8 (15) | 23.7 (15) | 6.3E-01 |
| YER183C | FAU1 | 24.8 (20) | 24.4 (20) | 8.1E-01 | 0 (0) | 0 (0) | 0.0E+00 | 24.8 (20) | 24.4 (20) | 8.1E-01 |
| YLR377C | FBP1 | 26.4 (45) | 24.5 (95) | 3.9E-01 | 0 (0) | 0 (0) | 0.0E+00 | 26.4 (45) | 24.5 (95) | 3.9E-01 |
| YMR058W | FET3 | 22 (10) | 22.7 (10) | 1.0E+00 | 0 (0) | 0 (0) | 0.0E+00 | 22 (10) | 22.7 (10) | 1.0E+00 |
| YDR130C | FIN1 | 19.9 (10) | 29.6 (10) | 3.7E-02 | 0 (0) | 0 (0) | 0.0E+00 | 19.9 (10) | 29.6 (10) | 3.7E-02 |
| YIL131C | FKH1 | 29.9 (20) | 24.8 (25) | 1.1E-01 | 0 (0) | 0 (0) | 0.0E+00 | 29.9 (20) | 24.8 (25) | 1.1E-01 |
| YAR050W | FLO1 | 21 (5) | 28.8 (5) | 4.1E-01 | 0 (0) | 0 (0) | 0.0E+00 | 21 (5) | 28.8 (5) | 4.1E-01 |
| YER182W | FMP10 | 23.2 (20) | 24.4 (20) | 3.8E-01 | 0 (0) | 0 (0) | 0.0E+00 | 23.2 (20) | 24.4 (20) | 3.8E-01 |
| YDR070C | FMP16 | 18.6 (10) | 29.6 (10) | 7.2E-03 | 0 (0) | 0 (0) | 0.0E+00 | 18.6 (10) | 29.6 (10) | 7.2E-03 |
| YKR065C | FMP18 | 18.8 (5) | 29 (5) | 3.1E-01 | 0 (0) | 0 (0) | 0.0E+00 | 18.8 (5) | 29 (5) | 3.1E-01 |
| YLR454W | FMP27 | 22.7 (20) | 26.9 (30) | 1.1E-01 | 0 (0) | 0 (0) | 0.0E+00 | 22.7 (20) | 26.9 (30) | 1.1E-01 |
| YDR493W | FMP36 | 22 (25) | 25.4 (35) | 7.6E-02 | 0 (0) | 0 (0) | 0.0E+00 | 22 (25) | 25.4 (35) | 7.6E-02 |
| YKR049C | FMP46 | 23.1 (20) | 28.4 (35) | 1.5E-02 | 0 (0) | 0 (0) | 0.0E+00 | 23.1 (20) | 28.4 (35) | 1.5E-02 |
| YBR262C | FMP51 | 17.3 (10) | 29.6 (10) | 1.7E-03 | 0 (0) | 0 (0) | 0.0E+00 | 17.3 (10) | 29.6 (10) | 1.7E-03 |
| YDR110W | FOB1 | 32 (1810) | 25.8 (1790) | 2.4E-56 | 31.8 (140) | 28.1 (100) | 2.1E-02 | 32 (1950) | 25.9 (1890) | 2.6E-57 |
| YLR449W | FPR4 | 28.6 (25) | 24.2 (25) | 1.4E-01 | 0 (0) | 0 (0) | 0.0E+00 | 28.6 (25) | 24.2 (25) | 1.4E-01 |
| YNR060W | FRE4 | 22.2 (5) | 28.8 (5) | 6.9E-01 | 0 (0) | 0 (0) | 0.0E+00 | 22.2 (5) | 28.8 (5) | 6.9E-01 |
| YCL058C | FYV5 | 21 (5) | 28.8 (5) | 6.9E-01 | 0 (0) | 0 (0) | 0.0E+00 | 21 (5) | 28.8 (5) | 6.9E-01 |
| YOR178C | GAC1 | 23.3 (10) | 29.3 (10) | 1.5E-01 | 0 (0) | 0 (0) | 0.0E+00 | 23.3 (10) | 29.3 (10) | 1.5E-01 |

| | | | | | | | | | | |
|---|---|---|---|---|---|---|---|---|---|---|
| YFL021W | GAT1 | 25.4 (25) | 23.9 (45) | 7.3E-01 | 0 (0) | 0 (0) | 0.0E+00 | 25.4 (25) | 23.9 (45) | 7.3E-01 |
| YDR283C | GCN2 | 25.7 (495) | 27 (565) | 2.8E-02 | 23.3 (180) | 28.8 (220) | 1.1E-08 | 25.1 (675) | 27.5 (785) | 8.5E-07 |
| YOR120W | GCY1 | 26.5 (15) | 25.1 (20) | 3.3E-01 | 0 (0) | 0 (0) | 0.0E+00 | 26.5 (15) | 25.1 (20) | 3.3E-01 |
| YHR108W | GGA2 | 26.6 (15) | 25 (25) | 4.9E-01 | 0 (0) | 0 (0) | 0.0E+00 | 26.6 (15) | 25 (25) | 4.9E-01 |
| YHR061C | GIC1 | 24.4 (5) | 22.4 (5) | 7.3E-01 | 0 (0) | 0 (0) | 0.0E+00 | 24.4 (5) | 22.4 (5) | 7.3E-01 |
| YDR309C | GIC2 | 24 (5) | 29 (5) | 5.5E-01 | 0 (0) | 0 (0) | 0.0E+00 | 24 (5) | 29 (5) | 5.5E-01 |
| YDR096W | GIS1 | 31.2 (90) | 25.2 (90) | 2.5E-04 | 0 (0) | 0 (0) | 0.0E+00 | 31.2 (90) | 25.2 (90) | 2.5E-04 |
| YKR058W | GLG1 | 25.8 (5) | 29 (5) | 8.3E-01 | 0 (0) | 0 (0) | 0.0E+00 | 25.8 (5) | 29 (5) | 8.3E-01 |
| YER040W | GLN3 | 20.6 (45) | 24.9 (45) | 1.5E-02 | 0 (0) | 0 (0) | 0.0E+00 | 20.6 (45) | 24.9 (45) | 1.5E-02 |
| YDR272W | GLO2 | 21.4 (5) | 29 (5) | 3.4E-01 | 0 (0) | 0 (0) | 0.0E+00 | 21.4 (5) | 29 (5) | 3.4E-01 |
| YHR183W | GND1 | 24.2 (5) | 22.4 (5) | 8.4E-01 | 0 (0) | 0 (0) | 0.0E+00 | 24.2 (5) | 22.4 (5) | 8.4E-01 |
| YBR244W | GPX2 | 19.5 (10) | 29.6 (10) | 2.3E-02 | 0 (0) | 0 (0) | 0.0E+00 | 19.5 (10) | 29.6 (10) | 2.3E-02 |
| YHR104W | GRE3 | 18.6 (5) | 22.4 (5) | 6.0E-01 | 0 (0) | 0 (0) | 0.0E+00 | 18.6 (5) | 22.4 (5) | 6.0E-01 |
| YDR098C | GRX3 | 27.1 (15) | 28.5 (15) | 1.5E-01 | 0 (0) | 0 (0) | 0.0E+00 | 27.1 (15) | 28.5 (15) | 1.5E-01 |
| YER174C | GRX4 | 26.6 (10) | 22.7 (10) | 2.0E-01 | 0 (0) | 0 (0) | 0.0E+00 | 26.6 (10) | 22.7 (10) | 2.0E-01 |
| YDR108W | GSG1 | 24.7 (15) | 28.5 (15) | 8.1E-02 | 0 (0) | 0 (0) | 0.0E+00 | 24.7 (15) | 28.5 (15) | 8.1E-02 |
| YDR295C | HDA2 | 31.9 (20) | 26.9 (35) | 1.7E-01 | 0 (0) | 0 (0) | 0.0E+00 | 31.9 (20) | 26.9 (35) | 1.7E-01 |
| YBR248C | HIS7 | 24.9 (10) | 29.6 (10) | 2.9E-01 | 0 (0) | 0 (0) | 0.0E+00 | 24.9 (10) | 29.6 (10) | 2.9E-01 |
| YLR450W | HMG2 | 30 (25) | 24.6 (25) | 8.4E-02 | 0 (0) | 0 (0) | 0.0E+00 | 30 (25) | 24.6 (25) | 8.4E-02 |
| YDR305C | HNT2 | 23 (5) | 29 (5) | 5.9E-01 | 0 (0) | 0 (0) | 0.0E+00 | 23 (5) | 29 (5) | 5.9E-01 |
| YDR258C | HSP78 | 25.2 (10) | 27.4 (10) | 6.2E-01 | 0 (0) | 0 (0) | 0.0E+00 | 25.2 (10) | 27.4 (10) | 6.2E-01 |
| YNR032CA | HUB1 | 23.6 (5) | 28.8 (5) | 6.0E-01 | 0 (0) | 0 (0) | 0.0E+00 | 23.6 (5) | 28.8 (5) | 6.0E-01 |
| YHR094C | HXT1 | 17.4 (5) | 22.4 (5) | 2.9E-01 | 0 (0) | 0 (0) | 0.0E+00 | 17.4 (5) | 22.4 (5) | 2.9E-01 |
| YHR092C | HXT4 | 21.4 (5) | 22.4 (5) | 8.8E-01 | 0 (0) | 0 (0) | 0.0E+00 | 21.4 (5) | 22.4 (5) | 8.8E-01 |
| YOR126C | IAH1 | 24.2 (5) | 32.8 (5) | 5.4E-01 | 0 (0) | 0 (0) | 0.0E+00 | 24.2 (5) | 32.8 (5) | 5.4E-01 |
| YOR136W | IDH2 | 31.8 (200) | 26.1 (235) | 2.7E-09 | 30 (80) | 25.7 (100) | 5.2E-03 | 31.3 (280) | 26 (335) | 6.1E-11 |
| YLR384C | IKI3 | 27.5 (10) | 22.7 (10) | 3.4E-01 | 0 (0) | 0 (0) | 0.0E+00 | 27.5 (10) | 22.7 (10) | 3.4E-01 |
| YLR432W | IMD3 | 24.8 (20) | 26.9 (30) | 4.3E-01 | 0 (0) | 0 (0) | 0.0E+00 | 24.8 (20) | 26.9 (30) | 4.3E-01 |
| YHR046C | INM1 | 36.1 (20) | 23.2 (25) | 8.1E-04 | 22.7 (40) | 25.3 (60) | 3.2E-02 | 27.2 (60) | 24.6 (85) | 8.4E-01 |
| YDR123C | INO2 | 29.3 (50) | 26.8 (50) | 3.1E-01 | 0 (0) | 0 (0) | 0.0E+00 | 29.3 (50) | 26.8 (50) | 3.1E-01 |

| | | | | | | | | | | |
|---|---|---|---|---|---|---|---|---|---|---|
| YIL002C | INP51 | 30.1 (150) | 26.6 (150) | 4.1E-03 | 33.6 (140) | 30.4 (140) | 2.6E-02 | 31.8 (290) | 28.4 (290) | 1.4E-04 |
| YOR109W | INP53 | 32 (45) | 24.5 (45) | 1.1E-03 | 32.8 (140) | 29.8 (180) | 5.1E-02 | 32.6 (185) | 28.7 (225) | 2.5E-03 |
| YDR315C | IPK1 | 36.6 (20) | 25.9 (55) | 1.4E-03 | 25.6 (40) | 25.3 (60) | 8.3E-01 | 29.3 (60) | 25.6 (115) | 9.9E-02 |
| YDR072C | IPT1 | 20.6 (10) | 29.6 (10) | 2.5E-02 | 0 (0) | 0 (0) | 0.0E+00 | 20.6 (10) | 29.6 (10) | 2.5E-02 |
| YHR079C | IRE1 | 27 (145) | 27.8 (145) | 6.2E-01 | 0 (0) | 0 (0) | 0.0E+00 | 27 (145) | 27.8 (145) | 6.2E-01 |
| YER180C | ISC10 | 22.9 (15) | 23.7 (15) | 7.2E-01 | 0 (0) | 0 (0) | 0.0E+00 | 22.9 (15) | 23.7 (15) | 7.2E-01 |
| YBR245C | ISW1 | 18.8 (10) | 29.6 (10) | 2.1E-02 | 0 (0) | 0 (0) | 0.0E+00 | 18.8 (10) | 29.6 (10) | 2.1E-02 |
| YHR158C | KEL1 | 27.7 (30) | 23.8 (65) | 1.4E-01 | 23 (20) | 23.7 (20) | 6.5E-01 | 25.8 (50) | 23.7 (85) | 5.0E-01 |
| YDR122W | KIN1 | 24.4 (40) | 25.9 (40) | 7.0E-01 | 0 (0) | 0 (0) | 0.0E+00 | 24.4 (40) | 25.9 (40) | 7.0E-01 |
| YHR082C | KSP1 | 27.7 (15) | 25 (25) | 4.2E-01 | 0 (0) | 0 (0) | 0.0E+00 | 27.7 (15) | 25 (25) | 4.2E-01 |
| YOR099W | KTR1 | 23.5 (10) | 29.3 (10) | 2.7E-01 | 0 (0) | 0 (0) | 0.0E+00 | 23.5 (10) | 29.3 (10) | 2.7E-01 |
| YKR061W | KTR2 | 15.4 (5) | 29 (5) | 1.5E-01 | 0 (0) | 0 (0) | 0.0E+00 | 15.4 (5) | 29 (5) | 1.5E-01 |
| YIL085C | KTR7 | 26.6 (20) | 24.4 (20) | 5.0E-01 | 0 (0) | 0 (0) | 0.0E+00 | 26.6 (20) | 24.4 (20) | 5.0E-01 |
| YOR171C | LCB4 | 29.9 (20) | 25.4 (65) | 1.6E-01 | 0 (0) | 0 (0) | 0.0E+00 | 29.9 (20) | 25.4 (65) | 1.6E-01 |
| YOR123C | LEO1 | 26.7 (15) | 27 (25) | 6.3E-01 | 0 (0) | 0 (0) | 0.0E+00 | 26.7 (15) | 27 (25) | 6.3E-01 |
| YOR108W | LEU9 | 27.3 (15) | 27 (25) | 1.0E+00 | 0 (0) | 0 (0) | 0.0E+00 | 27.3 (15) | 27 (25) | 1.0E+00 |
| YHR156C | LIN1 | 34.5 (15) | 25 (25) | 2.4E-02 | 27.7 (40) | 25.3 (60) | 3.5E-01 | 29.6 (55) | 25.2 (85) | 2.6E-02 |
| YHR081W | LRP1 | 24.4 (5) | 22.4 (5) | 6.0E-01 | 0 (0) | 0 (0) | 0.0E+00 | 24.4 (5) | 22.4 (5) | 6.0E-01 |
| YOR142W | LSC1 | 23.9 (10) | 29.3 (10) | 2.9E-01 | 0 (0) | 0 (0) | 0.0E+00 | 23.9 (10) | 29.3 (10) | 2.9E-01 |
| YGR057C | LST7 | 18.4 (5) | 32.8 (5) | 8.7E-02 | 0 (0) | 0 (0) | 0.0E+00 | 18.4 (5) | 32.8 (5) | 8.7E-02 |
| YAL024C | LTE1 | 27.9 (15) | 23.7 (15) | 3.3E-01 | 0 (0) | 0 (0) | 0.0E+00 | 27.9 (15) | 23.7 (15) | 3.3E-01 |
| YIL094C | LYS12 | 29.5 (45) | 25.1 (80) | 1.7E-01 | 27.1 (80) | 27.7 (120) | 4.9E-01 | 28 (125) | 26.6 (200) | 4.7E-01 |
| YNR050C | LYS9 | 31.2 (20) | 24.8 (50) | 2.4E-02 | 27.3 (40) | 25.3 (60) | 8.4E-01 | 28.6 (60) | 25 (110) | 8.1E-02 |
| YLR427W | MAG2 | 23.8 (5) | 28.8 (5) | 8.6E-01 | 0 (0) | 0 (0) | 0.0E+00 | 23.8 (5) | 28.8 (5) | 8.6E-01 |
| YBR299W | MAL32 | 23.8 (10) | 22.7 (10) | 1.0E+00 | 0 (0) | 0 (0) | 0.0E+00 | 23.8 (10) | 22.7 (10) | 1.0E+00 |
| YDR318W | MCM21 | 28.8 (20) | 26.9 (35) | 6.9E-01 | 0 (0) | 0 (0) | 0.0E+00 | 28.8 (20) | 26.9 (35) | 6.9E-01 |
| YBR227C | MCX1 | 20 (10) | 29.6 (10) | 5.3E-02 | 0 (0) | 0 (0) | 0.0E+00 | 20 (10) | 29.6 (10) | 5.3E-02 |
| YLR368W | MDM30 | 28.7 (20) | 23.8 (20) | 2.0E-01 | 0 (0) | 0 (0) | 0.0E+00 | 28.7 (20) | 23.8 (20) | 2.0E-01 |
| YGR100W | MDR1 | 23.8 (5) | 32.8 (5) | 3.1E-01 | 0 (0) | 0 (0) | 0.0E+00 | 23.8 (5) | 32.8 (5) | 3.1E-01 |

| | | | | | | | | | | |
|---|---|---|---|---|---|---|---|---|---|---|
| YGR121C | MEP1 | 24.3 (10) | 29.3 (10) | 2.1E-01 | 0 (0) | 0 (0) | 0.0E+00 | 24.3 (10) | 29.3 (10) | 2.1E-01 |
| YDR253C | MET32 | 28.7 (15) | 29.1 (20) | 5.1E-01 | 0 (0) | 0 (0) | 0.0E+00 | 28.7 (15) | 29.1 (20) | 5.1E-01 |
| YDR461W | MFA1 | 23.3 (20) | 24.4 (20) | 6.5E-01 | 0 (0) | 0 (0) | 0.0E+00 | 23.3 (20) | 24.4 (20) | 6.5E-01 |
| YDR296W | MHR1 | 23 (5) | 29 (5) | 4.6E-01 | 0 (0) | 0 (0) | 0.0E+00 | 23 (5) | 29 (5) | 4.6E-01 |
| YHR015W | MIP6 | 20.8 (5) | 22.4 (5) | 8.4E-01 | 0 (0) | 0 (0) | 0.0E+00 | 20.8 (5) | 22.4 (5) | 8.4E-01 |
| YDR245W | MNN10 | 32.5 (20) | 25.9 (55) | 1.1E-01 | 31.3 (20) | 27.7 (40) | 1.3E-01 | 31.9 (40) | 26.7 (95) | 2.2E-02 |
| YNR059W | MNT4 | 23.8 (5) | 28.8 (5) | 8.0E-01 | 0 (0) | 0 (0) | 0.0E+00 | 23.8 (5) | 28.8 (5) | 8.0E-01 |
| YOR177C | MPC54 | 32.7 (25) | 28.4 (25) | 1.6E-01 | 0 (0) | 0 (0) | 0.0E+00 | 32.7 (25) | 28.4 (25) | 1.6E-01 |
| YGR084C | MRP13 | 24.6 (5) | 32.8 (5) | 4.0E-01 | 0 (0) | 0 (0) | 0.0E+00 | 24.6 (5) | 32.8 (5) | 4.0E-01 |
| YDR116C | MRPL1 | 23.5 (10) | 29.6 (10) | 6.9E-02 | 0 (0) | 0 (0) | 0.0E+00 | 23.5 (10) | 29.6 (10) | 6.9E-02 |
| YDR322W | MRPL35 | 22.4 (5) | 29 (5) | 4.1E-01 | 0 (0) | 0 (0) | 0.0E+00 | 22.4 (5) | 29 (5) | 4.1E-01 |
| YML009C | MRPL39 | 21.4 (20) | 26.9 (30) | 3.2E-02 | 0 (0) | 0 (0) | 0.0E+00 | 21.4 (20) | 26.9 (30) | 3.2E-02 |
| YDR337W | MRPS28 | 34.3 (15) | 29.4 (15) | 3.3E-01 | 0 (0) | 0 (0) | 0.0E+00 | 34.3 (15) | 29.4 (15) | 3.3E-01 |
| YKR052C | MRS4 | 19.1 (20) | 27.2 (25) | 3.3E-03 | 0 (0) | 0 (0) | 0.0E+00 | 19.1 (20) | 27.2 (25) | 3.3E-03 |
| YHR039C | MSC7 | 18.6 (5) | 22.4 (5) | 4.5E-01 | 0 (0) | 0 (0) | 0.0E+00 | 18.6 (5) | 22.4 (5) | 4.5E-01 |
| YDR097C | MSH6 | 30.7 (20) | 26.2 (30) | 1.2E-01 | 0 (0) | 0 (0) | 0.0E+00 | 30.7 (20) | 26.2 (30) | 1.2E-01 |
| YDR335W | MSN5 | 29.1 (15) | 29.4 (15) | 8.2E-01 | 0 (0) | 0 (0) | 0.0E+00 | 29.1 (15) | 29.4 (15) | 8.2E-01 |
| YDR268W | MSW1 | 35 (35) | 26.9 (35) | 2.8E-03 | 30.2 (20) | 21.3 (20) | 1.2E-02 | 33.2 (55) | 24.8 (55) | 1.1E-04 |
| YDR277C | MTH1 | 28.9 (30) | 28.4 (35) | 8.9E-01 | 0 (0) | 0 (0) | 0.0E+00 | 28.9 (30) | 28.4 (35) | 8.9E-01 |
| YGR055W | MUP1 | 24.3 (15) | 27 (25) | 3.6E-01 | 0 (0) | 0 (0) | 0.0E+00 | 24.3 (15) | 27 (25) | 3.6E-01 |
| YHR086W | NAM8 | 24.9 (25) | 22.5 (45) | 3.4E-01 | 0 (0) | 0 (0) | 0.0E+00 | 24.9 (25) | 22.5 (45) | 3.4E-01 |
| YKR048C | NAP1 | 24.6 (45) | 27.2 (45) | 6.3E-02 | 0 (0) | 0 (0) | 0.0E+00 | 24.6 (45) | 27.2 (45) | 6.3E-02 |
| YHR124W | NDT80 | 24.2 (5) | 22.4 (5) | 6.6E-01 | 0 (0) | 0 (0) | 0.0E+00 | 24.2 (5) | 22.4 (5) | 6.6E-01 |
| YOR156C | NFI1 | 23.1 (10) | 29.3 (10) | 2.6E-01 | 0 (0) | 0 (0) | 0.0E+00 | 23.1 (10) | 29.3 (10) | 2.6E-01 |
| YKR103W | NFT1 | 26.1 (20) | 26.1 (25) | 9.5E-01 | 0 (0) | 0 (0) | 0.0E+00 | 26.1 (20) | 26.1 (25) | 9.5E-01 |
| YKR104W | NFT1 | 22.4 (5) | 28.8 (5) | 6.0E-01 | 0 (0) | 0 (0) | 0.0E+00 | 22.4 (5) | 28.8 (5) | 6.0E-01 |
| YLR351C | NIT3 | 27.8 (20) | 24.4 (20) | 2.5E-01 | 0 (0) | 0 (0) | 0.0E+00 | 27.8 (20) | 24.4 (20) | 2.5E-01 |
| YHR077C | NMD2 | 24 (5) | 22.4 (5) | 1.0E+00 | 0 (0) | 0 (0) | 0.0E+00 | 24 (5) | 22.4 (5) | 1.0E+00 |
| YLR363C | NMD4 | 29.5 (25) | 24 (55) | 3.3E-02 | 16.9 (40) | 21.1 (20) | 2.5E-02 | 21.8 (65) | 23.2 (75) | 1.1E-01 |
| YHR133C | NSG1 | 24.6 (5) | 22.4 (5) | 7.5E-01 | 0 (0) | 0 (0) | 0.0E+00 | 24.6 (5) | 22.4 (5) | 7.5E-01 |
| YIL136W | OM45 | 31.2 (35) | 25.4 (35) | 1.3E-01 | 19.2 (40) | 21.1 (20) | 3.7E-01 | 24.8 (75) | 23.8 (55) | 9.1E-01 |

| | | | | | | | | | | |
|---|---|---|---|---|---|---|---|---|---|---|
| YLR350W | ORM2 | 22.6 (10) | 22.7 (10) | 7.0E-01 | 0 (0) | 0 (0) | 0.0E+00 | 22.6 (10) | 22.7 (10) | 7.0E-01 |
| YOR130C | ORT1 | 27.8 (25) | 28.4 (25) | 3.8E-01 | 0 (0) | 0 (0) | 0.0E+00 | 27.8 (25) | 28.4 (25) | 3.8E-01 |
| YHR073W | OSH3 | 24.3 (15) | 25 (25) | 4.3E-01 | 0 (0) | 0 (0) | 0.0E+00 | 24.3 (15) | 25 (25) | 4.3E-01 |
| YHR179W | OYE2 | 18.2 (5) | 22.4 (5) | 4.5E-01 | 0 (0) | 0 (0) | 0.0E+00 | 18.2 (5) | 22.4 (5) | 4.5E-01 |
| YGR078C | PAC10 | 21.3 (35) | 26.2 (45) | 3.8E-02 | 0 (0) | 0 (0) | 0.0E+00 | 21.3 (35) | 26.2 (45) | 3.8E-02 |
| YMR174C | PAI3 | 28.3 (25) | 27.5 (35) | 9.9E-01 | 0 (0) | 0 (0) | 0.0E+00 | 28.3 (25) | 27.5 (35) | 9.9E-01 |
| YDR251W | PAM1 | 29.9 (20) | 26.9 (35) | 2.2E-01 | 0 (0) | 0 (0) | 0.0E+00 | 29.9 (20) | 26.9 (35) | 2.2E-01 |
| YLR461W | PAU4 | 32.1 (20) | 26.9 (30) | 8.3E-02 | 0 (0) | 0 (0) | 0.0E+00 | 32.1 (20) | 26.9 (30) | 8.3E-02 |
| YBR233W | PBP2 | 24 (10) | 29.6 (10) | 1.2E-01 | 0 (0) | 0 (0) | 0.0E+00 | 24 (10) | 29.6 (10) | 1.2E-01 |
| YKR097W | PCK1 | 20.2 (5) | 28.8 (5) | 2.6E-01 | 0 (0) | 0 (0) | 0.0E+00 | 20.2 (5) | 28.8 (5) | 2.6E-01 |
| YGR101W | PCP1 | 16.8 (5) | 32.8 (5) | 9.5E-02 | 0 (0) | 0 (0) | 0.0E+00 | 16.8 (5) | 32.8 (5) | 9.5E-02 |
| YER178W | PDA1 | 18.7 (40) | 23.2 (70) | 5.3E-03 | 0 (0) | 0 (0) | 0.0E+00 | 18.7 (40) | 23.2 (70) | 5.3E-03 |
| YGR087C | PDC6 | 24.5 (25) | 24.2 (45) | 7.6E-01 | 0 (0) | 0 (0) | 0.0E+00 | 24.5 (25) | 24.2 (45) | 7.6E-01 |
| YOR153W | PDR5 | 23.1 (10) | 29.3 (10) | 2.1E-01 | 0 (0) | 0 (0) | 0.0E+00 | 23.1 (10) | 29.3 (10) | 2.1E-01 |
| YDR323C | PEP7 | 24.5 (10) | 27.4 (10) | 6.2E-01 | 0 (0) | 0 (0) | 0.0E+00 | 24.5 (10) | 27.4 (10) | 6.2E-01 |
| YDR079W | PET100 | 25.2 (10) | 29.6 (10) | 3.1E-01 | 0 (0) | 0 (0) | 0.0E+00 | 25.2 (10) | 29.6 (10) | 3.1E-01 |
| YJR034W | PET191 | 23 (15) | 23.7 (15) | 5.5E-01 | 0 (0) | 0 (0) | 0.0E+00 | 23 (15) | 23.7 (15) | 5.5E-01 |
| YDR265W | PEX10 | 26.8 (25) | 28.1 (25) | 7.2E-01 | 0 (0) | 0 (0) | 0.0E+00 | 26.8 (25) | 28.1 (25) | 7.2E-01 |
| YHR160C | PEX18 | 22.6 (5) | 22.4 (5) | 7.9E-01 | 0 (0) | 0 (0) | 0.0E+00 | 22.6 (5) | 22.4 (5) | 7.9E-01 |
| YDR329C | PEX3 | 22.8 (5) | 29 (5) | 3.4E-01 | 0 (0) | 0 (0) | 0.0E+00 | 22.8 (5) | 29 (5) | 3.4E-01 |
| YDR244W | PEX5 | 27.3 (20) | 26.9 (35) | 8.7E-01 | 25.7 (20) | 28.7 (20) | 4.0E-01 | 26.5 (40) | 27.5 (55) | 6.5E-01 |
| YGR077C | PEX8 | 19.6 (5) | 32.8 (5) | 1.7E-01 | 0 (0) | 0 (0) | 0.0E+00 | 19.6 (5) | 32.8 (5) | 1.7E-01 |
| YDR281C | PHM6 | 29.7 (20) | 26.9 (35) | 3.0E-01 | 0 (0) | 0 (0) | 0.0E+00 | 29.7 (20) | 26.9 (35) | 3.0E-01 |
| YDR313C | PIB1 | 34.9 (55) | 27.9 (55) | 5.3E-04 | 28.4 (100) | 26.8 (120) | 3.4E-01 | 30.7 (155) | 27.2 (175) | 3.0E-03 |
| YHR034W | PIH1 | 19.2 (5) | 22.4 (5) | 4.0E-01 | 0 (0) | 0 (0) | 0.0E+00 | 19.2 (5) | 22.4 (5) | 4.0E-01 |
| YOR104W | PIN2 | 23.6 (5) | 32.8 (5) | 4.2E-01 | 0 (0) | 0 (0) | 0.0E+00 | 23.6 (5) | 32.8 (5) | 4.2E-01 |
| YDR276C | PMP3 | 28.6 (15) | 29.1 (20) | 9.9E-01 | 0 (0) | 0 (0) | 0.0E+00 | 28.6 (15) | 29.1 (20) | 9.9E-01 |
| YIL122W | POG1 | 21.1 (15) | 23.7 (15) | 2.7E-01 | 0 (0) | 0 (0) | 0.0E+00 | 21.1 (15) | 23.7 (15) | 2.7E-01 |
| YHR075C | PPE1 | 25.7 (15) | 25 (25) | 6.8E-01 | 0 (0) | 0 (0) | 0.0E+00 | 25.7 (15) | 25 (25) | 6.8E-01 |
| YDR075W | PPH3 | 23.8 (10) | 29.6 (10) | 4.4E-02 | 0 (0) | 0 (0) | 0.0E+00 | 23.8 (10) | 29.6 (10) | 4.4E-02 |

| | | | | | | | | | | |
|---|---|---|---|---|---|---|---|---|---|---|
| YDR300C | PRO1 | 28.8 (20) | 26.9 (35) | 9.5E-01 | 0 (0) | 0 (0) | 0.0E+00 | 28.8 (20) | 26.9 (35) | 9.5E-01 |
| YDR055W | PST1 | 25.3 (15) | 28.5 (15) | 2.0E-01 | 0 (0) | 0 (0) | 0.0E+00 | 25.3 (15) | 28.5 (15) | 2.0E-01 |
| YLR376C | PSY3 | 23.6 (15) | 23.7 (15) | 9.5E-01 | 0 (0) | 0 (0) | 0.0E+00 | 23.6 (15) | 23.7 (15) | 9.5E-01 |
| YER089C | PTC2 | 21.8 (15) | 23.7 (15) | 3.2E-01 | 0 (0) | 0 (0) | 0.0E+00 | 21.8 (15) | 23.7 (15) | 3.2E-01 |
| YHR076W | PTC7 | 24 (5) | 22.4 (5) | 9.4E-01 | 0 (0) | 0 (0) | 0.0E+00 | 24 (5) | 22.4 (5) | 9.4E-01 |
| YKR093W | PTR2 | 26.8 (20) | 26.1 (25) | 5.8E-01 | 26.2 (20) | 30.9 (20) | 9.3E-02 | 26.5 (40) | 28.2 (45) | 4.3E-01 |
| YHR037W | PUT2 | 22 (5) | 22.4 (5) | 1.0E+00 | 0 (0) | 0 (0) | 0.0E+00 | 22 (5) | 22.4 (5) | 1.0E+00 |
| YKR090W | PXL1 | 23.2 (5) | 28.8 (5) | 1.0E+00 | 0 (0) | 0 (0) | 0.0E+00 | 23.2 (5) | 28.8 (5) | 1.0E+00 |
| YER173W | RAD24 | 20 (10) | 22.7 (10) | 4.3E-01 | 0 (0) | 0 (0) | 0.0E+00 | 20 (10) | 22.7 (10) | 4.3E-01 |
| YDR076W | RAD55 | 12 (10) | 29.6 (10) | 6.6E-04 | 0 (0) | 0 (0) | 0.0E+00 | 12 (10) | 29.6 (10) | 6.6E-04 |
| YOR101W | RAS1 | 30.6 (60) | 26.7 (75) | 3.8E-02 | 21.5 (40) | 23.8 (40) | 4.2E-01 | 26.9 (100) | 25.7 (115) | 4.3E-01 |
| YHR157W | REC104 | 26.9 (15) | 25 (25) | 7.6E-01 | 0 (0) | 0 (0) | 0.0E+00 | 26.9 (15) | 25 (25) | 7.6E-01 |
| YBR260C | RGD1 | 24.6 (10) | 29.6 (10) | 1.6E-01 | 0 (0) | 0 (0) | 0.0E+00 | 24.6 (10) | 29.6 (10) | 1.6E-01 |
| YOR107W | RGS2 | 34.2 (25) | 25.3 (25) | 2.3E-03 | 23.1 (40) | 25.3 (60) | 1.3E-01 | 27.3 (65) | 25.3 (85) | 5.1E-01 |
| YLR453C | RIF2 | 23.7 (20) | 26.9 (30) | 2.7E-01 | 0 (0) | 0 (0) | 0.0E+00 | 23.7 (20) | 26.9 (30) | 2.7E-01 |
| YDR255C | RMD5 | 27.5 (15) | 27.1 (15) | 7.7E-01 | 0 (0) | 0 (0) | 0.0E+00 | 27.5 (15) | 27.1 (15) | 7.7E-01 |
| YDR257C | RMS1 | 27.5 (45) | 26.4 (45) | 8.3E-01 | 0 (0) | 0 (0) | 0.0E+00 | 27.5 (45) | 26.4 (45) | 8.3E-01 |
| YDR279W | RNH202 | 25.1 (15) | 29.1 (20) | 9.5E-02 | 0 (0) | 0 (0) | 0.0E+00 | 25.1 (15) | 29.1 (20) | 9.5E-02 |
| YGR070W | ROM1 | 24.9 (20) | 25.9 (65) | 4.8E-01 | 0 (0) | 0 (0) | 0.0E+00 | 24.9 (20) | 25.9 (65) | 4.8E-01 |
| YLR371W | ROM2 | 38 (50) | 25.5 (50) | 1.8E-08 | 29.3 (40) | 25.3 (60) | 4.7E-02 | 34.2 (90) | 25.4 (110) | 1.0E-08 |
| YBR229C | ROT2 | 23.1 (10) | 29.6 (10) | 1.4E-01 | 0 (0) | 0 (0) | 0.0E+00 | 23.1 (10) | 29.6 (10) | 1.4E-01 |
| YGR085C | RPL11B | 27.6 (25) | 25 (45) | 6.1E-01 | 0 (0) | 0 (0) | 0.0E+00 | 27.6 (25) | 25 (45) | 6.1E-01 |
| YLR344W | RPL26A | 26.8 (40) | 24.3 (65) | 2.8E-01 | 0 (0) | 0 (0) | 0.0E+00 | 26.8 (40) | 24.3 (65) | 2.8E-01 |
| YDL075W | RPL31A | 34.8 (455) | 26 (375) | 6.0E-21 | 37.6 (120) | 25.7 (180) | 5.0E-17 | 35.4 (575) | 25.9 (555) | 1.8E-34 |
| YDR500C | RPL37B | 31.5 (40) | 23.3 (55) | 1.2E-04 | 29.7 (80) | 24.4 (80) | 3.8E-03 | 30.3 (120) | 23.9 (135) | 6.9E-06 |
| YLR448W | RPL6B | 29.6 (130) | 26 (170) | 2.9E-03 | 33.5 (40) | 25.3 (60) | 1.1E-04 | 30.5 (170) | 25.8 (230) | 9.8E-06 |
| YLR441C | RPS1A | 29.9 (50) | 27 (70) | 3.7E-01 | 26 (40) | 25.3 (60) | 6.0E-01 | 28.1 (90) | 26.2 (130) | 7.1E-01 |
| YKR057W | RPS21A | 28.5 (40) | 26.4 (95) | 5.9E-01 | 27.4 (60) | 26.4 (80) | 9.9E-01 | 27.9 (100) | 26.4 (175) | 7.6E-01 |
| YLR367W | RPS22B | 26.4 (35) | 25 (75) | 8.3E-01 | 23.3 (40) | 25.3 (60) | 9.0E-02 | 24.7 (75) | 25.1 (135) | 2.9E-01 |
| YGR118W | RPS23A | 26.8 (45) | 26.4 (65) | 3.4E-01 | 0 (0) | 0 (0) | 0.0E+00 | 26.8 (45) | 26.4 (65) | 3.4E-01 |

| | | | | | | | | | | |
|---|---|---|---|---|---|---|---|---|---|---|
| YHR021C | RPS27B | 21.2 (35) | 27 (45) | 4.2E-03 | 0 (0) | 0 (0) | 0.0E+00 | 21.2 (35) | 27 (45) | 4.2E-03 |
| YOR167C | RPS28A | 26.7 (35) | 26.4 (45) | 6.8E-01 | 0 (0) | 0 (0) | 0.0E+00 | 26.7 (35) | 26.4 (45) | 6.8E-01 |
| YLR388W | RPS29A | 24.1 (40) | 24.9 (70) | 8.4E-01 | 19.7 (20) | 24.2 (20) | 9.0E-02 | 22.6 (60) | 24.7 (90) | 1.7E-01 |
| YOR182C | RPS30B | 24.3 (15) | 28.8 (25) | 1.7E-01 | 0 (0) | 0 (0) | 0.0E+00 | 24.3 (15) | 28.8 (25) | 1.7E-01 |
| YHR038W | RRF1 | 23.8 (5) | 22.4 (5) | 6.4E-01 | 0 (0) | 0 (0) | 0.0E+00 | 23.8 (5) | 22.4 (5) | 6.4E-01 |
| YHR031C | RRM3 | 18.2 (25) | 25.7 (45) | 3.9E-04 | 22.8 (20) | 30.7 (40) | 4.7E-02 | 20.2 (45) | 28 (85) | 1.6E-04 |
| YDR083W | RRP8 | 29.5 (40) | 27.4 (50) | 5.8E-01 | 22.6 (40) | 21.1 (20) | 5.9E-01 | 26 (80) | 25.6 (70) | 9.6E-01 |
| YGR056W | RSC1 | 28 (25) | 25.3 (25) | 4.5E-01 | 0 (0) | 0 (0) | 0.0E+00 | 28 (25) | 25.3 (25) | 4.5E-01 |
| YLR357W | RSC2 | 20.4 (10) | 22.7 (10) | 5.4E-01 | 0 (0) | 0 (0) | 0.0E+00 | 20.4 (10) | 22.7 (10) | 5.4E-01 |
| YDR289C | RTT103 | 24.3 (15) | 29.1 (20) | 9.5E-02 | 0 (0) | 0 (0) | 0.0E+00 | 24.3 (15) | 29.1 (20) | 9.5E-02 |
| YHR154W | RTT107 | 18.3 (15) | 25 (25) | 4.2E-02 | 0 (0) | 0 (0) | 0.0E+00 | 18.3 (15) | 25 (25) | 4.2E-02 |
| YOR138C | RUP1 | 21.8 (5) | 32.8 (5) | 3.1E-01 | 0 (0) | 0 (0) | 0.0E+00 | 21.8 (5) | 32.8 (5) | 3.1E-01 |
| YDR129C | SAC6 | 14.7 (30) | 25.8 (60) | 2.4E-05 | 0 (0) | 0 (0) | 0.0E+00 | 14.7 (30) | 25.8 (60) | 2.4E-05 |
| YDR502C | SAM2 | 26.3 (35) | 24.5 (35) | 5.4E-01 | 0 (0) | 0 (0) | 0.0E+00 | 26.3 (35) | 24.5 (35) | 5.4E-01 |
| YHR103W | SBE22 | 24.4 (15) | 25 (25) | 7.9E-01 | 0 (0) | 0 (0) | 0.0E+00 | 24.4 (15) | 25 (25) | 7.9E-01 |
| YOR165W | SEY1 | 16.6 (5) | 32.8 (5) | 1.1E-01 | 0 (0) | 0 (0) | 0.0E+00 | 16.6 (5) | 32.8 (5) | 1.1E-01 |
| YOR140W | SFL1 | 23.9 (25) | 28.4 (25) | 8.5E-02 | 0 (0) | 0 (0) | 0.0E+00 | 23.9 (25) | 28.4 (25) | 8.5E-02 |
| YBR258C | SHG1 | 22.5 (10) | 29.6 (10) | 3.4E-02 | 0 (0) | 0 (0) | 0.0E+00 | 22.5 (10) | 29.6 (10) | 3.4E-02 |
| YBR263W | SHM1 | 28.5 (15) | 28.5 (15) | 6.9E-01 | 0 (0) | 0 (0) | 0.0E+00 | 28.5 (15) | 28.5 (15) | 6.9E-01 |
| YDR078C | SHU2 | 24.8 (10) | 29.6 (10) | 3.3E-01 | 0 (0) | 0 (0) | 0.0E+00 | 24.8 (10) | 29.6 (10) | 3.3E-01 |
| YGR112W | SHY1 | 14.8 (5) | 32.8 (5) | 5.6E-02 | 0 (0) | 0 (0) | 0.0E+00 | 14.8 (5) | 32.8 (5) | 5.6E-02 |
| YOR137C | SIA1 | 29.8 (25) | 28.4 (25) | 9.6E-01 | 0 (0) | 0 (0) | 0.0E+00 | 29.8 (25) | 28.4 (25) | 9.6E-01 |
| YMR175W | SIP18 | 18.6 (20) | 28.2 (30) | 5.6E-04 | 0 (0) | 0 (0) | 0.0E+00 | 18.6 (20) | 28.2 (30) | 5.6E-04 |
| YKR101W | SIR1 | 19.4 (5) | 28.8 (5) | 4.6E-01 | 0 (0) | 0 (0) | 0.0E+00 | 19.4 (5) | 28.8 (5) | 4.6E-01 |
| YDL047W | SIT4 | 25.9 (80) | 28.3 (85) | 2.3E-01 | 0 (0) | 0 (0) | 0.0E+00 | 25.9 (80) | 28.3 (85) | 2.3E-01 |
| YDR515W | SLF1 | 25.5 (20) | 24.4 (20) | 9.4E-01 | 0 (0) | 0 (0) | 0.0E+00 | 25.5 (20) | 24.4 (20) | 9.4E-01 |
| YHR030C | SLT2 | 24.2 (45) | 27 (45) | 8.6E-02 | 0 (0) | 0 (0) | 0.0E+00 | 24.2 (45) | 27 (45) | 8.6E-02 |
| YBR228W | SLX1 | 25.9 (35) | 24.1 (40) | 5.1E-01 | 0 (0) | 0 (0) | 0.0E+00 | 25.9 (35) | 24.1 (40) | 5.1E-01 |
| YGR081C | SLX9 | 22.2 (10) | 29.3 (10) | 1.8E-01 | 0 (0) | 0 (0) | 0.0E+00 | 22.2 (10) | 29.3 (10) | 1.8E-01 |
| YHR050W | SMF2 | 23.9 (25) | 26.3 (25) | 1.1E-01 | 0 (0) | 0 (0) | 0.0E+00 | 23.9 (25) | 26.3 (25) | 1.1E-01 |
| YDR477W | SNF1 | 38.8 (40) | 25.5 (40) | 2.4E-06 | 26.1 | 27 (120) | 3.5E-01 | 29.8 (140) | 26.6 (160) | 4.9E-02 |

| | | | | | | | | | | | |
|---|---|---|---|---|---|---|---|---|---|---|---|
| | | | | | (100) | | | | | | |
| YDR073W | SNF11 | 30.5 (30) | 28.4 (30) | 6.9E-01 | 0 (0) | 0 (0) | 0.0E+00 | 30.5 (30) | 28.4 (30) | 6.9E-01 |
| YHR163W | SOL3 | 28.9 (15) | 25 (25) | 7.5E-01 | 0 (0) | 0 (0) | 0.0E+00 | 28.9 (15) | 25 (25) | 7.5E-01 |
| YAL047C | SPC72 | 4 (5) | 28.8 (5) | 7.9E-03 | 0 (0) | 0 (0) | 0.0E+00 | 4 (5) | 28.8 (5) | 7.9E-03 |
| YHR136C | SPL2 | 15.6 (5) | 22.4 (5) | 1.3E-01 | 0 (0) | 0 (0) | 0.0E+00 | 15.6 (5) | 22.4 (5) | 1.3E-01 |
| YHR152W | SPO12 | 24.2 (5) | 22.4 (5) | 1.0E+00 | 0 (0) | 0 (0) | 0.0E+00 | 24.2 (5) | 22.4 (5) | 1.0E+00 |
| YHR014W | SPO13 | 28.3 (35) | 26.5 (45) | 4.1E-01 | 0 (0) | 0 (0) | 0.0E+00 | 28.3 (35) | 26.5 (45) | 4.1E-01 |
| YHR153C | SPO16 | 17 (5) | 22.4 (5) | 3.1E-01 | 0 (0) | 0 (0) | 0.0E+00 | 17 (5) | 22.4 (5) | 3.1E-01 |
| YDR104C | SPO71 | 27.3 (15) | 30.1 (20) | 3.9E-01 | 0 (0) | 0 (0) | 0.0E+00 | 27.3 (15) | 30.1 (20) | 3.9E-01 |
| YGR059W | SPR3 | 28.8 (25) | 25.3 (25) | 2.4E-01 | 0 (0) | 0 (0) | 0.0E+00 | 28.8 (25) | 25.3 (25) | 2.4E-01 |
| YHR139C | SPS100 | 27.5 (15) | 25 (25) | 9.6E-01 | 0 (0) | 0 (0) | 0.0E+00 | 27.5 (15) | 25 (25) | 9.6E-01 |
| YGR104C | SRB5 | 22.2 (5) | 32.8 (5) | 2.1E-01 | 0 (0) | 0 (0) | 0.0E+00 | 22.2 (5) | 32.8 (5) | 2.1E-01 |
| YKR091W | SRL3 | 18.4 (5) | 28.8 (5) | 2.5E-01 | 0 (0) | 0 (0) | 0.0E+00 | 18.4 (5) | 28.8 (5) | 2.5E-01 |
| YKR092C | SRP40 | 22.6 (25) | 24.2 (25) | 6.1E-01 | 0 (0) | 0 (0) | 0.0E+00 | 22.6 (25) | 24.2 (25) | 6.1E-01 |
| YDR293C | SSD1 | 26.7 (105) | 27.5 (105) | 4.9E-01 | 0 (0) | 0 (0) | 0.0E+00 | 26.7 (105) | 27.5 (105) | 4.9E-01 |
| YHR066W | SSF1 | 38.5 (20) | 24.6 (65) | 2.1E-03 | 27.4 (40) | 25.3 (60) | 3.3E-01 | 31.1 (60) | 24.9 (125) | 6.1E-03 |
| YDR312W | SSF2 | 22.3 (10) | 27.4 (10) | 4.7E-01 | 0 (0) | 0 (0) | 0.0E+00 | 22.3 (10) | 27.4 (10) | 4.7E-01 |
| YLR452C | SST2 | 31 (25) | 25.7 (25) | 6.8E-02 | 22.7 (40) | 21.1 (20) | 3.9E-01 | 25.9 (65) | 23.6 (45) | 3.0E-01 |
| YMR053C | STB2 | 29 (40) | 24.4 (40) | 3.1E-01 | 20.6 (60) | 21.2 (40) | 5.2E-01 | 24 (100) | 22.8 (80) | 8.8E-01 |
| YHR178W | STB5 | 20.2 (5) | 22.4 (5) | 4.0E-01 | 0 (0) | 0 (0) | 0.0E+00 | 20.2 (5) | 22.4 (5) | 4.0E-01 |
| YLR362W | STE11 | 22.7 (45) | 24.9 (50) | 1.6E-01 | 23.1 (20) | 30.9 (20) | 6.8E-03 | 22.8 (65) | 26.6 (70) | 1.2E-02 |
| YLR389C | STE23 | 24.2 (15) | 23.7 (15) | 9.5E-01 | 0 (0) | 0 (0) | 0.0E+00 | 24.2 (15) | 23.7 (15) | 9.5E-01 |
| YLR375W | STP3 | 24.8 (25) | 25.4 (35) | 2.4E-01 | 0 (0) | 0 (0) | 0.0E+00 | 24.8 (25) | 25.4 (35) | 2.4E-01 |
| YMR054W | STV1 | 24.1 (25) | 25.7 (40) | 4.6E-01 | 0 (0) | 0 (0) | 0.0E+00 | 24.1 (25) | 25.7 (40) | 4.6E-01 |
| YDR310C | SUM1 | 23.7 (10) | 27.4 (10) | 4.5E-01 | 0 (0) | 0 (0) | 0.0E+00 | 23.7 (10) | 27.4 (10) | 4.5E-01 |
| YDR297W | SUR2 | 29.8 (25) | 25.9 (25) | 6.8E-02 | 0 (0) | 0 (0) | 0.0E+00 | 29.8 (25) | 25.9 (25) | 6.8E-02 |
| YLR372W | SUR4 | 27.6 (35) | 24.5 (35) | 3.6E-01 | 0 (0) | 0 (0) | 0.0E+00 | 27.6 (35) | 24.5 (35) | 3.6E-01 |
| YDR320C | SWA2 | 26.2 (15) | 29.1 (20) | 2.9E-01 | 0 (0) | 0 (0) | 0.0E+00 | 26.2 (15) | 29.1 (20) | 2.9E-01 |
| YDR126W | SWF1 | 19.7 (20) | 27.8 (30) | 2.0E-03 | 0 (0) | 0 (0) | 0.0E+00 | 19.7 (20) | 27.8 (30) | 2.0E-03 |
| YDR260C | SWM1 | 31.1 (20) | 26.9 (35) | 1.9E-01 | 0 (0) | 0 (0) | 0.0E+00 | 31.1 (20) | 26.9 (35) | 1.9E-01 |
| YOR179C | SYC1 | 28.3 (25) | 28.4 (25) | 8.6E-01 | 0 (0) | 0 (0) | 0.0E+00 | 28.3 (25) | 28.4 (25) | 8.6E-01 |

| | | | | | | | | | | |
|---|---|---|---|---|---|---|---|---|---|---|
| YLR354C | TAL1 | 29.2 (45) | 24.5 (95) | 1.3E-02 | 0 (0) | 0 (0) | 0.0E+00 | 29.2 (45) | 24.5 (95) | 1.3E-02 |
| YBR223C | TDP1 | 22.4 (10) | 29.6 (10) | 1.8E-01 | 0 (0) | 0 (0) | 0.0E+00 | 22.4 (10) | 29.6 (10) | 1.8E-01 |
| YBR240C | THI2 | 32.5 (30) | 28.4 (30) | 3.4E-01 | 0 (0) | 0 (0) | 0.0E+00 | 32.5 (30) | 28.4 (30) | 3.4E-01 |
| YHR167W | THP2 | 19 (5) | 22.4 (5) | 3.1E-01 | 0 (0) | 0 (0) | 0.0E+00 | 19 (5) | 22.4 (5) | 3.1E-01 |
| YKR059W | TIF1 | 30.1 (75) | 26.7 (85) | 2.5E-02 | 27.2 (120) | 23.8 (100) | 6.6E-03 | 28.3 (195) | 25.1 (185) | 1.3E-03 |
| YDR105C | TMS1 | 23.9 (10) | 29.6 (10) | 1.5E-01 | 0 (0) | 0 (0) | 0.0E+00 | 23.9 (10) | 29.6 (10) | 1.5E-01 |
| YER175C | TMT1 | 23.8 (65) | 25 (75) | 1.1E-01 | 0 (0) | 0 (0) | 0.0E+00 | 23.8 (65) | 25 (75) | 1.1E-01 |
| YMR060C | TOM37 | 17.2 (5) | 28.8 (5) | 4.2E-01 | 0 (0) | 0 (0) | 0.0E+00 | 17.2 (5) | 28.8 (5) | 4.2E-01 |
| YHR117W | TOM71 | 24.6 (5) | 22.4 (5) | 6.9E-01 | 0 (0) | 0 (0) | 0.0E+00 | 24.6 (5) | 22.4 (5) | 6.9E-01 |
| YJR066W | TOR1 | 33.3 (630) | 25.3 (640) | 6.0E-40 | 34 (80) | 28.5 (100) | 3.5E-04 | 33.4 (710) | 25.7 (740) | 3.1E-42 |
| YGR096W | TPC1 | 21.7 (25) | 28.4 (25) | 1.4E-02 | 0 (0) | 0 (0) | 0.0E+00 | 21.7 (25) | 28.4 (25) | 1.4E-02 |
| YKR050W | TRK2 | 16.6 (5) | 29 (5) | 2.4E-01 | 0 (0) | 0 (0) | 0.0E+00 | 16.6 (5) | 29 (5) | 2.4E-01 |
| YDR120C | TRM1 | 22.5 (10) | 29.6 (10) | 1.5E-01 | 0 (0) | 0 (0) | 0.0E+00 | 22.5 (10) | 29.6 (10) | 1.5E-01 |
| YKR056W | TRM2 | 22.8 (10) | 27.4 (10) | 3.6E-01 | 0 (0) | 0 (0) | 0.0E+00 | 22.8 (10) | 27.4 (10) | 3.6E-01 |
| YHR106W | TRR2 | 26.3 (25) | 26.3 (25) | 9.1E-01 | 0 (0) | 0 (0) | 0.0E+00 | 26.3 (25) | 26.3 (25) | 9.1E-01 |
| YOR115C | TRS33 | 25.3 (10) | 29.3 (10) | 4.5E-01 | 0 (0) | 0 (0) | 0.0E+00 | 25.3 (10) | 29.3 (10) | 4.5E-01 |
| YLR425W | TUS1 | 17.6 (5) | 28.8 (5) | 2.1E-01 | 0 (0) | 0 (0) | 0.0E+00 | 17.6 (5) | 28.8 (5) | 2.1E-01 |
| YDR100W | TVP15 | 15.6 (10) | 29.6 (10) | 1.1E-03 | 0 (0) | 0 (0) | 0.0E+00 | 15.6 (10) | 29.6 (10) | 1.1E-03 |
| YDR084C | TVP23 | 21.4 (10) | 29.6 (10) | 1.2E-02 | 0 (0) | 0 (0) | 0.0E+00 | 21.4 (10) | 29.6 (10) | 1.2E-02 |
| YKR088C | TVP38 | 24.4 (5) | 28.8 (5) | 1.0E+00 | 0 (0) | 0 (0) | 0.0E+00 | 24.4 (5) | 28.8 (5) | 1.0E+00 |
| YGR080W | TWF1 | 17.4 (5) | 32.8 (5) | 5.6E-02 | 0 (0) | 0 (0) | 0.0E+00 | 17.4 (5) | 32.8 (5) | 5.6E-02 |
| YHR111W | UBA4 | 20 (5) | 22.4 (5) | 7.4E-01 | 0 (0) | 0 (0) | 0.0E+00 | 20 (5) | 22.4 (5) | 7.4E-01 |
| YDR092W | UBC13 | 22.2 (10) | 29.6 (10) | 1.6E-01 | 0 (0) | 0 (0) | 0.0E+00 | 22.2 (10) | 29.6 (10) | 1.6E-01 |
| YDR059C | UBC5 | 22.2 (10) | 29.6 (10) | 1.7E-01 | 0 (0) | 0 (0) | 0.0E+00 | 22.2 (10) | 29.6 (10) | 1.7E-01 |
| YKR098C | UBP11 | 21.6 (5) | 28.8 (5) | 5.7E-01 | 0 (0) | 0 (0) | 0.0E+00 | 21.6 (5) | 28.8 (5) | 5.7E-01 |
| YOR124C | UBP2 | 28.5 (15) | 27 (25) | 6.0E-01 | 0 (0) | 0 (0) | 0.0E+00 | 28.5 (15) | 27 (25) | 6.0E-01 |
| YML021C | UNG1 | 15.8 (5) | 28.8 (5) | 1.7E-01 | 0 (0) | 0 (0) | 0.0E+00 | 15.8 (5) | 28.8 (5) | 1.7E-01 |
| YGR072W | UPF3 | 24.6 (25) | 26.7 (25) | 2.5E-01 | 0 (0) | 0 (0) | 0.0E+00 | 24.6 (25) | 26.7 (25) | 2.5E-01 |
| YNL229C | URE2 | 31.9 (25) | 24.6 (45) | 1.2E-03 | 30.6 (40) | 25.3 (60) | 2.1E-02 | 31.1 (65) | 25 (105) | 3.7E-04 |
| YKR060W | UTP30 | 20.6 (5) | 29 (5) | 3.9E-01 | 0 (0) | 0 (0) | 0.0E+00 | 20.6 (5) | 29 (5) | 3.9E-01 |

| | | | | | | | | | | |
|---|---|---|---|---|---|---|---|---|---|---|
| YLR386W | VAC14 | 18.5 (10) | 22.7 (10) | 3.4E-01 | 0 (0) | 0 (0) | 0.0E+00 | 18.5 (10) | 22.7 (10) | 3.4E-01 |
| YOR106W | VAM3 | 14.4 (5) | 32.8 (5) | 3.2E-02 | 0 (0) | 0 (0) | 0.0E+00 | 14.4 (5) | 32.8 (5) | 3.2E-02 |
| YDR247W | VHS1 | 34 (20) | 25.9 (55) | 2.6E-02 | 25.4 (40) | 25.3 (60) | 4.0E-01 | 28.2 (60) | 25.6 (115) | 4.3E-01 |
| YLR373C | VID22 | 12.2 (10) | 22.7 (10) | 3.4E-02 | 0 (0) | 0 (0) | 0.0E+00 | 12.2 (10) | 22.7 (10) | 3.4E-02 |
| YGR105W | VMA21 | 10.7 (14) | 24.9 (45) | 2.7E-06 | 0 (0) | 0 (0) | 0.0E+00 | 10.7 (14) | 24.9 (45) | 2.7E-06 |
| YHR060W | VMA22 | 12.6 (5) | 22.4 (5) | 5.6E-02 | 0 (0) | 0 (0) | 0.0E+00 | 12.6 (5) | 22.4 (5) | 5.6E-02 |
| YLR447C | VMA6 | 12 (5) | 28.8 (5) | 3.2E-02 | 0 (0) | 0 (0) | 0.0E+00 | 12 (5) | 28.8 (5) | 3.2E-02 |
| YOR132W | VPS17 | 25.8 (5) | 32.8 (5) | 4.5E-01 | 0 (0) | 0 (0) | 0.0E+00 | 25.8 (5) | 32.8 (5) | 4.5E-01 |
| YLR360W | VPS38 | 29.3 (25) | 25.4 (35) | 5.5E-01 | 0 (0) | 0 (0) | 0.0E+00 | 29.3 (25) | 25.4 (35) | 5.5E-01 |
| YDR080W | VPS41 | 16.7 (10) | 29.6 (10) | 4.5E-03 | 0 (0) | 0 (0) | 0.0E+00 | 16.7 (10) | 29.6 (10) | 4.5E-03 |
| YJR044C | VPS55 | 26.8 (20) | 24.4 (20) | 7.6E-01 | 0 (0) | 0 (0) | 0.0E+00 | 26.8 (20) | 24.4 (20) | 7.6E-01 |
| YHR134W | WSS1 | 14.2 (5) | 22.4 (5) | 1.5E-01 | 0 (0) | 0 (0) | 0.0E+00 | 14.2 (5) | 22.4 (5) | 1.5E-01 |
| YHR161C | YAP1801 | 25.7 (15) | 25 (25) | 7.3E-01 | 0 (0) | 0 (0) | 0.0E+00 | 25.7 (15) | 25 (25) | 7.3E-01 |
| YDR259C | YAP6 | 24.1 (10) | 27.4 (10) | 5.7E-01 | 0 (0) | 0 (0) | 0.0E+00 | 24.1 (10) | 27.4 (10) | 5.7E-01 |
| YHR135C | YCK1 | 23.4 (5) | 22.4 (5) | 6.9E-01 | 0 (0) | 0 (0) | 0.0E+00 | 23.4 (5) | 22.4 (5) | 6.9E-01 |
| YDR057W | YOS9 | 26.2 (90) | 29 (90) | 9.5E-03 | 0 (0) | 0 (0) | 0.0E+00 | 26.2 (90) | 29 (90) | 9.5E-03 |
| YBR264C | YPT10 | 25 (30) | 26.5 (30) | 6.0E-01 | 0 (0) | 0 (0) | 0.0E+00 | 25 (30) | 26.5 (30) | 6.0E-01 |
| YHR105W | YPT35 | 28.8 (25) | 24.6 (65) | 4.1E-01 | 0 (0) | 0 (0) | 0.0E+00 | 28.8 (25) | 24.6 (65) | 4.1E-01 |
| YOR172W | YRM1 | 30.6 (25) | 28.4 (25) | 5.4E-01 | 0 (0) | 0 (0) | 0.0E+00 | 30.6 (25) | 28.4 (25) | 5.4E-01 |
| YOR162C | YRR1 | 29.7 (20) | 29 (35) | 7.1E-01 | 0 (0) | 0 (0) | 0.0E+00 | 29.7 (20) | 29 (35) | 7.1E-01 |
| YNR065C | YSN1 | 24.8 (5) | 28.8 (5) | 8.9E-01 | 0 (0) | 0 (0) | 0.0E+00 | 24.8 (5) | 28.8 (5) | 8.9E-01 |
| YDR285W | ZIP1 | 28.3 (20) | 27.2 (25) | 8.8E-01 | 21.2 (20) | 29.7 (20) | 2.7E-02 | 24.7 (40) | 28.3 (45) | 8.9E-02 |
| YAL058CA | | 23.6 (5) | 28.8 (5) | 9.5E-01 | 0 (0) | 0 (0) | 0.0E+00 | 23.6 (5) | 28.8 (5) | 9.5E-01 |
| YBR224W | | 24.3 (10) | 29.6 (10) | 1.7E-01 | 0 (0) | 0 (0) | 0.0E+00 | 24.3 (10) | 29.6 (10) | 1.7E-01 |
| YBR225W | | 17.6 (10) | 29.6 (10) | 2.5E-02 | 0 (0) | 0 (0) | 0.0E+00 | 17.6 (10) | 29.6 (10) | 2.5E-02 |
| YBR226C | | 31.8 (20) | 29.6 (20) | 4.9E-01 | 0 (0) | 0 (0) | 0.0E+00 | 31.8 (20) | 29.6 (20) | 4.9E-01 |
| YBR230C | | 21.4 (10) | 29.6 (10) | 1.5E-01 | 0 (0) | 0 (0) | 0.0E+00 | 21.4 (10) | 29.6 (10) | 1.5E-01 |
| YBR235W | | 24.3 (10) | 29.6 (10) | 6.8E-02 | 0 (0) | 0 (0) | 0.0E+00 | 24.3 (10) | 29.6 (10) | 6.8E-02 |
| YBR238C | | 34.9 (20) | 27.9 (20) | 1.3E-02 | 33.9 (40) | 25.3 (60) | 1.9E-03 | 34.2 (60) | 25.9 (80) | 4.4E-05 |
| YBR239C | | 24.1 (10) | 29.6 (10) | 3.6E-01 | 0 (0) | 0 (0) | 0.0E+00 | 24.1 (10) | 29.6 (10) | 3.6E-01 |
| YBR241C | | 23.8 (10) | 29.6 (10) | 2.4E-01 | 0 (0) | 0 (0) | 0.0E+00 | 23.8 (10) | 29.6 (10) | 2.4E-01 |

| | | | | | | | | | |
|---|---|---|---|---|---|---|---|---|---|
| YBR242W | 27.7 (25) | 26.6 (30) | 5.7E-01 | 0 (0) | 0 (0) | 0.0E+00 | 27.7 (25) | 26.6 (30) | 5.7E-01 |
| YBR246W | 25 (10) | 29.6 (10) | 3.1E-01 | 0 (0) | 0 (0) | 0.0E+00 | 25 (10) | 29.6 (10) | 3.1E-01 |
| YBR250W | 25.5 (10) | 29.6 (10) | 2.7E-01 | 0 (0) | 0 (0) | 0.0E+00 | 25.5 (10) | 29.6 (10) | 2.7E-01 |
| YBR255W | 34.1 (20) | 25.5 (50) | 1.7E-03 | 31.6 (40) | 25.3 (60) | 2.1E-03 | 32.4 (60) | 25.3 (110) | 1.3E-05 |
| YBR259W | 27.1 (30) | 28.4 (30) | 4.5E-01 | 0 (0) | 0 (0) | 0.0E+00 | 27.1 (30) | 28.4 (30) | 4.5E-01 |
| YBR261C | 33.1 (20) | 27.9 (20) | 1.8E-01 | 0 (0) | 0 (0) | 0.0E+00 | 33.1 (20) | 27.9 (20) | 1.8E-01 |
| YBR266C | 37.3 (80) | 27.3 (80) | 1.4E-07 | 36.4 (40) | 24.8 (40) | 1.4E-06 | 37 (120) | 26.5 (120) | 1.5E-12 |
| YBR267W | 37.5 (50) | 27.3 (50) | 4.6E-05 | 36.5 (40) | 24.8 (40) | 1.9E-05 | 37.1 (90) | 26.2 (90) | 3.7E-09 |
| YCL006C | 31.5 (25) | 25.1 (55) | 3.2E-02 | 0 (0) | 0 (0) | 0.0E+00 | 31.5 (25) | 25.1 (55) | 3.2E-02 |
| YCL022C | 22 (5) | 28.8 (5) | 4.6E-01 | 0 (0) | 0 (0) | 0.0E+00 | 22 (5) | 28.8 (5) | 4.6E-01 |
| YCL023C | 31.7 (10) | 28.5 (10) | 4.3E-01 | 0 (0) | 0 (0) | 0.0E+00 | 31.7 (10) | 28.5 (10) | 4.3E-01 |
| YCL074W | 17.8 (5) | 28.8 (5) | 3.8E-01 | 0 (0) | 0 (0) | 0.0E+00 | 17.8 (5) | 28.8 (5) | 3.8E-01 |
| YCL075W | 28.4 (30) | 26.9 (30) | 7.5E-01 | 0 (0) | 0 (0) | 0.0E+00 | 28.4 (30) | 26.9 (30) | 7.5E-01 |
| YCL076W | 23 (5) | 28.8 (5) | 6.9E-01 | 0 (0) | 0 (0) | 0.0E+00 | 23 (5) | 28.8 (5) | 6.9E-01 |
| YDL061W | 25.5 (10) | 22.7 (10) | 4.3E-01 | 0 (0) | 0 (0) | 0.0E+00 | 25.5 (10) | 22.7 (10) | 4.3E-01 |
| YDR049W | 22.4 (10) | 29.6 (10) | 4.5E-02 | 0 (0) | 0 (0) | 0.0E+00 | 22.4 (10) | 29.6 (10) | 4.5E-02 |
| YDR051C | 22.1 (10) | 29.6 (10) | 5.3E-02 | 0 (0) | 0 (0) | 0.0E+00 | 22.1 (10) | 29.6 (10) | 5.3E-02 |
| YDR056C | 28.7 (20) | 26.9 (30) | 7.8E-01 | 0 (0) | 0 (0) | 0.0E+00 | 28.7 (20) | 26.9 (30) | 7.8E-01 |
| YDR061W | 25.1 (10) | 29.6 (10) | 2.7E-01 | 0 (0) | 0 (0) | 0.0E+00 | 25.1 (10) | 29.6 (10) | 2.7E-01 |
| YDR063W | 23.8 (10) | 29.6 (10) | 6.9E-02 | 0 (0) | 0 (0) | 0.0E+00 | 23.8 (10) | 29.6 (10) | 6.9E-02 |
| YDR065W | 15.6 (10) | 29.6 (10) | 2.8E-03 | 0 (0) | 0 (0) | 0.0E+00 | 15.6 (10) | 29.6 (10) | 2.8E-03 |
| YDR066C | 27.5 (15) | 28.5 (15) | 9.7E-01 | 0 (0) | 0 (0) | 0.0E+00 | 27.5 (15) | 28.5 (15) | 9.7E-01 |
| YDR067C | 25.6 (10) | 29.6 (10) | 5.2E-01 | 0 (0) | 0 (0) | 0.0E+00 | 25.6 (10) | 29.6 (10) | 5.2E-01 |
| YDR089W | 20.8 (10) | 29.6 (10) | 3.4E-02 | 0 (0) | 0 (0) | 0.0E+00 | 20.8 (10) | 29.6 (10) | 3.4E-02 |
| YDR090C | 25 (20) | 27.6 (35) | 1.0E-01 | 0 (0) | 0 (0) | 0.0E+00 | 25 (20) | 27.6 (35) | 1.0E-01 |
| YDR094W | 23.6 (10) | 29.6 (10) | 1.4E-01 | 0 (0) | 0 (0) | 0.0E+00 | 23.6 (10) | 29.6 (10) | 1.4E-01 |
| YDR095C | 20.1 (10) | 29.6 (10) | 3.6E-03 | 0 (0) | 0 (0) | 0.0E+00 | 20.1 (10) | 29.6 (10) | 3.6E-03 |
| YDR102C | 26.7 (20) | 29.6 (20) | 2.3E-01 | 0 (0) | 0 (0) | 0.0E+00 | 26.7 (20) | 29.6 (20) | 2.3E-01 |
| YDR107C | 24.7 (10) | 29.6 (10) | 4.5E-01 | 0 (0) | 0 (0) | 0.0E+00 | 24.7 (10) | 29.6 (10) | 4.5E-01 |
| YDR109C | 25 (10) | 29.6 (10) | 2.0E-01 | 0 (0) | 0 (0) | 0.0E+00 | 25 (10) | 29.6 (10) | 2.0E-01 |
| YDR112W | 20.9 (10) | 29.6 (10) | 4.5E-02 | 0 (0) | 0 (0) | 0.0E+00 | 20.9 (10) | 29.6 (10) | 4.5E-02 |

| | | | | | | | | | |
|---|---|---|---|---|---|---|---|---|---|
| YDR117C | 23.2 (10) | 29.6 (10) | 1.7E-01 | 0 (0) | 0 (0) | 0.0E+00 | 23.2 (10) | 29.6 (10) | 1.7E-01 |
| YDR119W | 29.4 (25) | 29.9 (30) | 7.7E-01 | 0 (0) | 0 (0) | 0.0E+00 | 29.4 (25) | 29.9 (30) | 7.7E-01 |
| YDR124W | 35.4 (20) | 27.9 (20) | 1.5E-02 | 22.6 (40) | 25.3 (60) | 8.2E-02 | 26.8 (60) | 25.9 (80) | 9.2E-01 |
| YDR128W | 19.6 (10) | 29.6 (10) | 8.0E-03 | 0 (0) | 0 (0) | 0.0E+00 | 19.6 (10) | 29.6 (10) | 8.0E-03 |
| YDR131C | 24.7 (10) | 29.6 (10) | 1.6E-01 | 0 (0) | 0 (0) | 0.0E+00 | 24.7 (10) | 29.6 (10) | 1.6E-01 |
| YDR132C | 17.9 (10) | 29.6 (10) | 1.0E-02 | 0 (0) | 0 (0) | 0.0E+00 | 17.9 (10) | 29.6 (10) | 1.0E-02 |
| YDR133C | 22.9 (10) | 29.6 (10) | 1.2E-01 | 0 (0) | 0 (0) | 0.0E+00 | 22.9 (10) | 29.6 (10) | 1.2E-01 |
| YDR134C | 19.8 (10) | 29.6 (10) | 1.0E-02 | 0 (0) | 0 (0) | 0.0E+00 | 19.8 (10) | 29.6 (10) | 1.0E-02 |
| YDR248C | 34.6 (25) | 29.5 (25) | 2.5E-02 | 24 (40) | 25.3 (60) | 2.4E-01 | 28.1 (65) | 26.5 (85) | 4.6E-01 |
| YDR249C | 25 (15) | 29.1 (20) | 6.6E-02 | 0 (0) | 0 (0) | 0.0E+00 | 25 (15) | 29.1 (20) | 6.6E-02 |
| YDR250C | 33.5 (30) | 28.4 (35) | 5.1E-02 | 0 (0) | 0 (0) | 0.0E+00 | 33.5 (30) | 28.4 (35) | 5.1E-02 |
| YDR262W | 21.4 (5) | 29 (5) | 4.6E-01 | 0 (0) | 0 (0) | 0.0E+00 | 21.4 (5) | 29 (5) | 4.6E-01 |
| YDR266C | 30.4 (20) | 26.9 (35) | 3.0E-01 | 0 (0) | 0 (0) | 0.0E+00 | 30.4 (20) | 26.9 (35) | 3.0E-01 |
| YDR274C | 35.6 (30) | 28.4 (35) | 8.7E-03 | 19.7 (40) | 21.1 (20) | 2.1E-01 | 26.5 (70) | 25.7 (55) | 8.0E-01 |
| YDR278C | 34.1 (15) | 29.4 (15) | 3.8E-01 | 0 (0) | 0 (0) | 0.0E+00 | 34.1 (15) | 29.4 (15) | 3.8E-01 |
| YDR282C | 17 (5) | 29 (5) | 9.5E-02 | 0 (0) | 0 (0) | 0.0E+00 | 17 (5) | 29 (5) | 9.5E-02 |
| YDR286C | 28 (20) | 26.9 (35) | 8.3E-01 | 0 (0) | 0 (0) | 0.0E+00 | 28 (20) | 26.9 (35) | 8.3E-01 |
| YDR287W | 10 (5) | 29 (5) | 3.2E-02 | 0 (0) | 0 (0) | 0.0E+00 | 10 (5) | 29 (5) | 3.2E-02 |
| YDR291W | 18.6 (5) | 29 (5) | 2.5E-01 | 0 (0) | 0 (0) | 0.0E+00 | 18.6 (5) | 29 (5) | 2.5E-01 |
| YDR306C | 27.8 (20) | 26.9 (35) | 6.5E-01 | 0 (0) | 0 (0) | 0.0E+00 | 27.8 (20) | 26.9 (35) | 6.5E-01 |
| YDR307W | 35.1 (20) | 26.9 (35) | 5.6E-03 | 23.2 (40) | 23.8 (40) | 7.1E-01 | 27.2 (60) | 25.2 (75) | 4.7E-01 |
| YDR314C | 35.9 (15) | 29.4 (15) | 4.5E-01 | 29.7 (20) | 21.3 (20) | 4.7E-03 | 32.4 (35) | 24.7 (35) | 1.1E-02 |
| YDR316W | 16.8 (5) | 29 (5) | 1.5E-01 | 0 (0) | 0 (0) | 0.0E+00 | 16.8 (5) | 29 (5) | 1.5E-01 |
| YDR317W | 28.1 (20) | 26.9 (35) | 6.7E-01 | 0 (0) | 0 (0) | 0.0E+00 | 28.1 (20) | 26.9 (35) | 6.7E-01 |
| YDR319C | 30.7 (20) | 26.9 (35) | 3.4E-01 | 0 (0) | 0 (0) | 0.0E+00 | 30.7 (20) | 26.9 (35) | 3.4E-01 |
| YDR326C | 28.4 (25) | 25.4 (35) | 4.0E-01 | 0 (0) | 0 (0) | 0.0E+00 | 28.4 (25) | 25.4 (35) | 4.0E-01 |
| YDR330W | 25.4 (5) | 29 (5) | 1.0E+00 | 0 (0) | 0 (0) | 0.0E+00 | 25.4 (5) | 29 (5) | 1.0E+00 |
| YDR333C | 19.2 (5) | 29 (5) | 2.1E-01 | 0 (0) | 0 (0) | 0.0E+00 | 19.2 (5) | 29 (5) | 2.1E-01 |
| YDR334W | 24.1 (30) | 26.9 (30) | 1.1E-01 | 0 (0) | 0 (0) | 0.0E+00 | 24.1 (30) | 26.9 (30) | 1.1E-01 |
| YDR336W | 26.8 (25) | 27.6 (25) | 6.8E-01 | 0 (0) | 0 (0) | 0.0E+00 | 26.8 (25) | 27.6 (25) | 6.8E-01 |
| YDR417C | 28.5 (20) | 22.7 (20) | 6.2E-02 | 0 (0) | 0 (0) | 0.0E+00 | 28.5 (20) | 22.7 (20) | 6.2E-02 |

| | | | | | | | | | |
|---|---|---|---|---|---|---|---|---|---|
| YDR444W | 27.3 (10) | 22.7 (10) | 3.8E-01 | 0 (0) | 0 (0) | 0.0E+00 | 27.3 (10) | 22.7 (10) | 3.8E-01 |
| YDR506C | 27.3 (30) | 25.3 (40) | 5.1E-01 | 0 (0) | 0 (0) | 0.0E+00 | 27.3 (30) | 25.3 (40) | 5.1E-01 |
| YER181C | 27.6 (25) | 24 (55) | 3.1E-01 | 0 (0) | 0 (0) | 0.0E+00 | 27.6 (25) | 24 (55) | 3.1E-01 |
| YER184C | 21.2 (10) | 22.7 (10) | 4.0E-01 | 0 (0) | 0 (0) | 0.0E+00 | 21.2 (10) | 22.7 (10) | 4.0E-01 |
| YER185W | 25.6 (20) | 24.4 (20) | 9.9E-01 | 0 (0) | 0 (0) | 0.0E+00 | 25.6 (20) | 24.4 (20) | 9.9E-01 |
| YER186C | 31.6 (25) | 24 (55) | 8.0E-03 | 24.4 (40) | 25.3 (60) | 3.3E-01 | 27.2 (65) | 24.7 (115) | 2.8E-01 |
| YER187W | 32.8 (35) | 25.4 (35) | 2.5E-02 | 24 (40) | 25.3 (60) | 1.4E-01 | 28.1 (75) | 25.3 (95) | 5.1E-01 |
| YGL199C | 21.2 (10) | 25.1 (10) | 3.2E-01 | 0 (0) | 0 (0) | 0.0E+00 | 21.2 (10) | 25.1 (10) | 3.2E-01 |
| YGL214W | 31.7 (25) | 27.1 (35) | 2.0E-01 | 0 (0) | 0 (0) | 0.0E+00 | 31.7 (25) | 27.1 (35) | 2.0E-01 |
| YGL217C | 30.2 (25) | 27.1 (35) | 3.5E-01 | 0 (0) | 0 (0) | 0.0E+00 | 30.2 (25) | 27.1 (35) | 3.5E-01 |
| YGL235W | 32.6 (25) | 27.1 (35) | 4.0E-02 | 0 (0) | 0 (0) | 0.0E+00 | 32.6 (25) | 27.1 (35) | 4.0E-02 |
| YGR011W | 26.8 (25) | 27.1 (35) | 8.1E-01 | 0 (0) | 0 (0) | 0.0E+00 | 26.8 (25) | 27.1 (35) | 8.1E-01 |
| YGR018C | 25.7 (10) | 28.5 (10) | 8.8E-01 | 0 (0) | 0 (0) | 0.0E+00 | 25.7 (10) | 28.5 (10) | 8.8E-01 |
| YGR022C | 23.7 (10) | 28.5 (10) | 6.0E-01 | 0 (0) | 0 (0) | 0.0E+00 | 23.7 (10) | 28.5 (10) | 6.0E-01 |
| YGR054W | 23.7 (10) | 29.3 (10) | 2.9E-01 | 0 (0) | 0 (0) | 0.0E+00 | 23.7 (10) | 29.3 (10) | 2.9E-01 |
| YGR058W | 19.8 (5) | 32.8 (5) | 1.6E-01 | 0 (0) | 0 (0) | 0.0E+00 | 19.8 (5) | 32.8 (5) | 1.6E-01 |
| YGR064W | 27.4 (25) | 28.4 (25) | 8.5E-01 | 0 (0) | 0 (0) | 0.0E+00 | 27.4 (25) | 28.4 (25) | 8.5E-01 |
| YGR066C | 25.5 (20) | 25.9 (65) | 3.5E-01 | 0 (0) | 0 (0) | 0.0E+00 | 25.5 (20) | 25.9 (65) | 3.5E-01 |
| YGR067C | 27.2 (15) | 27 (25) | 9.7E-01 | 0 (0) | 0 (0) | 0.0E+00 | 27.2 (15) | 27 (25) | 9.7E-01 |
| YGR068C | 26.6 (25) | 25.3 (25) | 8.2E-01 | 0 (0) | 0 (0) | 0.0E+00 | 26.6 (25) | 25.3 (25) | 8.2E-01 |
| YGR069W | 27.8 (25) | 25.3 (25) | 2.1E-01 | 0 (0) | 0 (0) | 0.0E+00 | 27.8 (25) | 25.3 (25) | 2.1E-01 |
| YGR071C | 13.8 (5) | 32.8 (5) | 3.2E-02 | 0 (0) | 0 (0) | 0.0E+00 | 13.8 (5) | 32.8 (5) | 3.2E-02 |
| YGR079W | 22.6 (10) | 29.3 (10) | 1.2E-01 | 0 (0) | 0 (0) | 0.0E+00 | 22.6 (10) | 29.3 (10) | 1.2E-01 |
| YGR107W | 29.8 (25) | 25.3 (25) | 3.5E-01 | 0 (0) | 0 (0) | 0.0E+00 | 29.8 (25) | 25.3 (25) | 3.5E-01 |
| YGR111W | 22.2 (5) | 32.8 (5) | 2.9E-01 | 0 (0) | 0 (0) | 0.0E+00 | 22.2 (5) | 32.8 (5) | 2.9E-01 |
| YGR122W | 23.9 (10) | 29.3 (10) | 2.9E-01 | 0 (0) | 0 (0) | 0.0E+00 | 23.9 (10) | 29.3 (10) | 2.9E-01 |
| YHR022C | 21.8 (5) | 22.4 (5) | 8.4E-01 | 0 (0) | 0 (0) | 0.0E+00 | 21.8 (5) | 22.4 (5) | 8.4E-01 |
| YHR029C | 21 (5) | 22.4 (5) | 5.1E-01 | 0 (0) | 0 (0) | 0.0E+00 | 21 (5) | 22.4 (5) | 5.1E-01 |
| YHR033W | 23.8 (5) | 22.4 (5) | 8.7E-01 | 0 (0) | 0 (0) | 0.0E+00 | 23.8 (5) | 22.4 (5) | 8.7E-01 |
| YHR035W | 22 (5) | 22.4 (5) | 9.4E-01 | 0 (0) | 0 (0) | 0.0E+00 | 22 (5) | 22.4 (5) | 9.4E-01 |
| YHR048W | 23.6 (5) | 22.4 (5) | 8.9E-01 | 0 (0) | 0 (0) | 0.0E+00 | 23.6 (5) | 22.4 (5) | 8.9E-01 |

| | | | | | | | | | |
|---|---|---|---|---|---|---|---|---|---|
| YHR049CA | 24.4 (5) | 22.4 (5) | 4.8E-01 | 0 (0) | 0 (0) | 0.0E+00 | 24.4 (5) | 22.4 (5) | 4.8E-01 |
| YHR049W | 23.8 (5) | 22.4 (5) | 9.5E-01 | 0 (0) | 0 (0) | 0.0E+00 | 23.8 (5) | 22.4 (5) | 9.5E-01 |
| YHR078W | 21 (5) | 22.4 (5) | 1.0E+00 | 0 (0) | 0 (0) | 0.0E+00 | 21 (5) | 22.4 (5) | 1.0E+00 |
| YHR080C | 23.8 (5) | 22.4 (5) | 7.3E-01 | 0 (0) | 0 (0) | 0.0E+00 | 23.8 (5) | 22.4 (5) | 7.3E-01 |
| YHR087W | 23.1 (15) | 25 (25) | 5.1E-01 | 0 (0) | 0 (0) | 0.0E+00 | 23.1 (15) | 25 (25) | 5.1E-01 |
| YHR095W | 16.8 (5) | 22.4 (5) | 1.5E-01 | 0 (0) | 0 (0) | 0.0E+00 | 16.8 (5) | 22.4 (5) | 1.5E-01 |
| YHR096W | 26.5 (15) | 25 (25) | 9.2E-01 | 0 (0) | 0 (0) | 0.0E+00 | 26.5 (15) | 25 (25) | 9.2E-01 |
| YHR097C | 26.8 (15) | 25 (25) | 4.4E-01 | 0 (0) | 0 (0) | 0.0E+00 | 26.8 (15) | 25 (25) | 4.4E-01 |
| YHR100C | 15 (5) | 22.4 (5) | 2.2E-01 | 0 (0) | 0 (0) | 0.0E+00 | 15 (5) | 22.4 (5) | 2.2E-01 |
| YHR112C | 24.6 (5) | 22.4 (5) | 6.0E-01 | 0 (0) | 0 (0) | 0.0E+00 | 24.6 (5) | 22.4 (5) | 6.0E-01 |
| YHR113W | 28.6 (20) | 24.6 (65) | 2.4E-01 | 0 (0) | 0 (0) | 0.0E+00 | 28.6 (20) | 24.6 (65) | 2.4E-01 |
| YHR115C | 20.2 (5) | 22.4 (5) | 7.4E-01 | 0 (0) | 0 (0) | 0.0E+00 | 20.2 (5) | 22.4 (5) | 7.4E-01 |
| YHR116W | 10 (5) | 22.4 (5) | 1.6E-02 | 0 (0) | 0 (0) | 0.0E+00 | 10 (5) | 22.4 (5) | 1.6E-02 |
| YHR121W | 24.4 (5) | 22.4 (5) | 7.4E-01 | 0 (0) | 0 (0) | 0.0E+00 | 24.4 (5) | 22.4 (5) | 7.4E-01 |
| YHR125W | 20.6 (5) | 22.4 (5) | 6.5E-01 | 0 (0) | 0 (0) | 0.0E+00 | 20.6 (5) | 22.4 (5) | 6.5E-01 |
| YHR126C | 19 (25) | 26.3 (25) | 2.9E-03 | 0 (0) | 0 (0) | 0.0E+00 | 19 (25) | 26.3 (25) | 2.9E-03 |
| YHR130C | 35 (30) | 24.7 (35) | 1.1E-04 | 23.7 (40) | 25.3 (60) | 2.1E-01 | 28.6 (70) | 25 (95) | 1.0E-01 |
| YHR138C | 28 (25) | 26.3 (25) | 3.0E-01 | 0 (0) | 0 (0) | 0.0E+00 | 28 (25) | 26.3 (25) | 3.0E-01 |
| YHR139CA | 27.5 (15) | 25 (25) | 3.1E-01 | 0 (0) | 0 (0) | 0.0E+00 | 27.5 (15) | 25 (25) | 3.1E-01 |
| YHR151C | 27.2 (30) | 24.7 (35) | 4.6E-01 | 0 (0) | 0 (0) | 0.0E+00 | 27.2 (30) | 24.7 (35) | 4.6E-01 |
| YHR155W | 19.4 (5) | 22.4 (5) | 6.0E-01 | 0 (0) | 0 (0) | 0.0E+00 | 19.4 (5) | 22.4 (5) | 6.0E-01 |
| YHR159W | 22 (5) | 22.4 (5) | 8.9E-01 | 0 (0) | 0 (0) | 0.0E+00 | 22 (5) | 22.4 (5) | 8.9E-01 |
| YHR182W | 17.2 (5) | 22.4 (5) | 2.9E-01 | 0 (0) | 0 (0) | 0.0E+00 | 17.2 (5) | 22.4 (5) | 2.9E-01 |
| YIL001W | 31.8 (25) | 27.6 (25) | 1.5E-01 | 0 (0) | 0 (0) | 0.0E+00 | 31.8 (25) | 27.6 (25) | 1.5E-01 |
| YIL056W | 21 (10) | 22.7 (10) | 6.5E-01 | 0 (0) | 0 (0) | 0.0E+00 | 21 (10) | 22.7 (10) | 6.5E-01 |
| YIL102C | 26.5 (25) | 25.2 (25) | 8.2E-01 | 0 (0) | 0 (0) | 0.0E+00 | 26.5 (25) | 25.2 (25) | 8.2E-01 |
| YIL158W | 23.8 (25) | 25.4 (35) | 2.4E-01 | 0 (0) | 0 (0) | 0.0E+00 | 23.8 (25) | 25.4 (35) | 2.4E-01 |
| YJR039W | 25 (10) | 22.7 (10) | 3.6E-01 | 0 (0) | 0 (0) | 0.0E+00 | 25 (10) | 22.7 (10) | 3.6E-01 |
| YKR045C | 28.2 (20) | 28.4 (35) | 7.3E-01 | 0 (0) | 0 (0) | 0.0E+00 | 28.2 (20) | 28.4 (35) | 7.3E-01 |
| YKR047W | 23.2 (5) | 29 (5) | 6.5E-01 | 0 (0) | 0 (0) | 0.0E+00 | 23.2 (5) | 29 (5) | 6.5E-01 |
| YKR051W | 25.4 (5) | 29 (5) | 8.8E-01 | 0 (0) | 0 (0) | 0.0E+00 | 25.4 (5) | 29 (5) | 8.8E-01 |

| | | | | | | | | | |
|---|---|---|---|---|---|---|---|---|---|
| YKR064W | 25.6 (20) | 28.4 (35) | 1.3E-01 | 0 (0) | 0 (0) | 0.0E+00 | 25.6 (20) | 28.4 (35) | 1.3E-01 |
| YKR087C | 9.2 (5) | 28.8 (5) | 1.6E-02 | 0 (0) | 0 (0) | 0.0E+00 | 9.2 (5) | 28.8 (5) | 1.6E-02 |
| YKR089C | 23.3 (20) | 26.9 (30) | 1.1E-01 | 0 (0) | 0 (0) | 0.0E+00 | 23.3 (20) | 26.9 (30) | 1.1E-01 |
| YKR100C | 20.8 (5) | 28.8 (5) | 5.8E-01 | 0 (0) | 0 (0) | 0.0E+00 | 20.8 (5) | 28.8 (5) | 5.8E-01 |
| YKR105C | 29.3 (20) | 24.8 (50) | 8.3E-02 | 21.2 (40) | 21.1 (20) | 9.7E-01 | 23.9 (60) | 23.7 (70) | 9.9E-01 |
| YLR262CA | 29.8 (20) | 26.9 (30) | 3.3E-01 | 0 (0) | 0 (0) | 0.0E+00 | 29.8 (20) | 26.9 (30) | 3.3E-01 |
| YLR345W | 19.2 (15) | 23.7 (15) | 1.2E-01 | 0 (0) | 0 (0) | 0.0E+00 | 19.2 (15) | 23.7 (15) | 1.2E-01 |
| YLR349W | 30.1 (25) | 25.4 (35) | 1.4E-01 | 0 (0) | 0 (0) | 0.0E+00 | 30.1 (25) | 25.4 (35) | 1.4E-01 |
| YLR352W | 23.4 (25) | 24 (55) | 8.2E-01 | 0 (0) | 0 (0) | 0.0E+00 | 23.4 (25) | 24 (55) | 8.2E-01 |
| YLR356W | 19 (10) | 22.7 (10) | 3.0E-01 | 0 (0) | 0 (0) | 0.0E+00 | 19 (10) | 22.7 (10) | 3.0E-01 |
| YLR364W | 27.6 (25) | 25.4 (35) | 4.5E-01 | 0 (0) | 0 (0) | 0.0E+00 | 27.6 (25) | 25.4 (35) | 4.5E-01 |
| YLR365W | 23.8 (15) | 23.7 (15) | 7.4E-01 | 0 (0) | 0 (0) | 0.0E+00 | 23.8 (15) | 23.7 (15) | 7.4E-01 |
| YLR366W | 30.9 (30) | 25.5 (40) | 8.5E-02 | 0 (0) | 0 (0) | 0.0E+00 | 30.9 (30) | 25.5 (40) | 8.5E-02 |
| YLR374C | 19 (10) | 22.7 (10) | 7.6E-01 | 0 (0) | 0 (0) | 0.0E+00 | 19 (10) | 22.7 (10) | 7.6E-01 |
| YLR385C | 26.4 (25) | 25.4 (35) | 7.7E-01 | 0 (0) | 0 (0) | 0.0E+00 | 26.4 (25) | 25.4 (35) | 7.7E-01 |
| YLR387C | 25.9 (25) | 25.4 (35) | 6.1E-01 | 0 (0) | 0 (0) | 0.0E+00 | 25.9 (25) | 25.4 (35) | 6.1E-01 |
| YLR422W | 33.9 (30) | 26.9 (30) | 2.4E-02 | 20.8 (40) | 21.1 (20) | 7.0E-01 | 26.4 (70) | 24.5 (50) | 6.5E-01 |
| YLR426W | 18.6 (25) | 25.8 (25) | 7.6E-03 | 0 (0) | 0 (0) | 0.0E+00 | 18.6 (25) | 25.8 (25) | 7.6E-03 |
| YLR428C | 28.2 (20) | 26.9 (30) | 7.7E-01 | 0 (0) | 0 (0) | 0.0E+00 | 28.2 (20) | 26.9 (30) | 7.7E-01 |
| YLR434C | 22.8 (5) | 28.8 (5) | 4.5E-01 | 0 (0) | 0 (0) | 0.0E+00 | 22.8 (5) | 28.8 (5) | 4.5E-01 |
| YLR437C | 21.8 (20) | 26.9 (30) | 5.0E-02 | 0 (0) | 0 (0) | 0.0E+00 | 21.8 (20) | 26.9 (30) | 5.0E-02 |
| YLR444C | 23.4 (20) | 26.9 (30) | 1.1E-01 | 0 (0) | 0 (0) | 0.0E+00 | 23.4 (20) | 26.9 (30) | 1.1E-01 |
| YLR445W | 13 (5) | 28.8 (5) | 3.2E-02 | 0 (0) | 0 (0) | 0.0E+00 | 13 (5) | 28.8 (5) | 3.2E-02 |
| YLR446W | 24.2 (5) | 28.8 (5) | 6.9E-01 | 0 (0) | 0 (0) | 0.0E+00 | 24.2 (5) | 28.8 (5) | 6.9E-01 |
| YLR456W | 21.6 (5) | 28.8 (5) | 5.2E-01 | 0 (0) | 0 (0) | 0.0E+00 | 21.6 (5) | 28.8 (5) | 5.2E-01 |
| YLR460C | 19.5 (20) | 26.9 (30) | 7.2E-03 | 0 (0) | 0 (0) | 0.0E+00 | 19.5 (20) | 26.9 (30) | 7.2E-03 |
| YML010CB | 23.9 (30) | 26.9 (30) | 2.4E-01 | 0 (0) | 0 (0) | 0.0E+00 | 23.9 (30) | 26.9 (30) | 2.4E-01 |
| YMR052CA | 26.2 (25) | 25.4 (35) | 8.9E-01 | 0 (0) | 0 (0) | 0.0E+00 | 26.2 (25) | 25.4 (35) | 8.9E-01 |
| YMR057C | 28.2 (25) | 24 (55) | 4.4E-01 | 0 (0) | 0 (0) | 0.0E+00 | 28.2 (25) | 24 (55) | 4.4E-01 |
| YMR158CB | 19.6 (5) | 28.8 (5) | 2.9E-01 | 0 (0) | 0 (0) | 0.0E+00 | 19.6 (5) | 28.8 (5) | 2.9E-01 |
| YMR194CA | 21.4 (5) | 28.8 (5) | 6.9E-01 | 0 (0) | 0 (0) | 0.0E+00 | 21.4 (5) | 28.8 (5) | 6.9E-01 |

| | | | | | | | | | |
|---|---|---|---|---|---|---|---|---|---|
| YMR326C | 15.6 (5) | 28.8 (5) | 1.3E-01 | 0 (0) | 0 (0) | 0.0E+00 | 15.6 (5) | 28.8 (5) | 1.3E-01 |
| YNR061C | 26.9 (20) | 26.9 (30) | 4.6E-01 | 0 (0) | 0 (0) | 0.0E+00 | 26.9 (20) | 26.9 (30) | 4.6E-01 |
| YNR062C | 23.4 (20) | 26.9 (30) | 1.2E-01 | 0 (0) | 0 (0) | 0.0E+00 | 23.4 (20) | 26.9 (30) | 1.2E-01 |
| YNR063W | 22.4 (5) | 28.8 (5) | 6.8E-01 | 0 (0) | 0 (0) | 0.0E+00 | 22.4 (5) | 28.8 (5) | 6.8E-01 |
| YNR064C | 18.2 (5) | 28.8 (5) | 3.3E-01 | 0 (0) | 0 (0) | 0.0E+00 | 18.2 (5) | 28.8 (5) | 3.3E-01 |
| YNR066C | 14 (5) | 28.8 (5) | 4.0E-02 | 0 (0) | 0 (0) | 0.0E+00 | 14 (5) | 28.8 (5) | 4.0E-02 |
| YNR068C | 12.4 (5) | 28.8 (5) | 5.6E-02 | 0 (0) | 0 (0) | 0.0E+00 | 12.4 (5) | 28.8 (5) | 5.6E-02 |
| YOR008CA | 29.2 (20) | 26.9 (30) | 1.9E-01 | 0 (0) | 0 (0) | 0.0E+00 | 29.2 (20) | 26.9 (30) | 1.9E-01 |
| YOR097C | 30.6 (25) | 25.3 (25) | 2.2E-01 | 0 (0) | 0 (0) | 0.0E+00 | 30.6 (25) | 25.3 (25) | 2.2E-01 |
| YOR105W | 25.3 (25) | 25.3 (25) | 8.2E-01 | 0 (0) | 0 (0) | 0.0E+00 | 25.3 (25) | 25.3 (25) | 8.2E-01 |
| YOR111W | 28.3 (40) | 25.4 (85) | 2.3E-01 | 32.1 (20) | 31 (40) | 7.2E-01 | 29.5 (60) | 27.2 (125) | 2.1E-01 |
| YOR112W | 25.8 (25) | 25.3 (25) | 9.4E-01 | 0 (0) | 0 (0) | 0.0E+00 | 25.8 (25) | 25.3 (25) | 9.4E-01 |
| YOR114W | 25.8 (5) | 32.8 (5) | 5.5E-01 | 0 (0) | 0 (0) | 0.0E+00 | 25.8 (5) | 32.8 (5) | 5.5E-01 |
| YOR118W | 28.6 (20) | 25.9 (65) | 6.0E-01 | 0 (0) | 0 (0) | 0.0E+00 | 28.6 (20) | 25.9 (65) | 6.0E-01 |
| YOR121C | 21.2 (5) | 32.8 (5) | 2.2E-01 | 0 (0) | 0 (0) | 0.0E+00 | 21.2 (5) | 32.8 (5) | 2.2E-01 |
| YOR129C | 18 (5) | 32.8 (5) | 1.3E-01 | 0 (0) | 0 (0) | 0.0E+00 | 18 (5) | 32.8 (5) | 1.3E-01 |
| YOR131C | 23.7 (10) | 29.3 (10) | 3.6E-01 | 0 (0) | 0 (0) | 0.0E+00 | 23.7 (10) | 29.3 (10) | 3.6E-01 |
| YOR135C | 31 (20) | 25.4 (65) | 7.3E-02 | 29.5 (40) | 24.8 (40) | 2.4E-03 | 30 (60) | 25.2 (105) | 3.2E-04 |
| YOR139C | 25.6 (10) | 29.3 (10) | 5.0E-01 | 0 (0) | 0 (0) | 0.0E+00 | 25.6 (10) | 29.3 (10) | 5.0E-01 |
| YOR152C | 21.8 (5) | 32.8 (5) | 1.3E-01 | 0 (0) | 0 (0) | 0.0E+00 | 21.8 (5) | 32.8 (5) | 1.3E-01 |
| YOR154W | 25.6 (5) | 32.8 (5) | 3.8E-01 | 0 (0) | 0 (0) | 0.0E+00 | 25.6 (5) | 32.8 (5) | 3.8E-01 |
| YOR161C | 22.9 (10) | 29.3 (10) | 1.6E-01 | 0 (0) | 0 (0) | 0.0E+00 | 22.9 (10) | 29.3 (10) | 1.6E-01 |
| YOR164C | 22.6 (5) | 32.8 (5) | 3.1E-01 | 0 (0) | 0 (0) | 0.0E+00 | 22.6 (5) | 32.8 (5) | 3.1E-01 |
| YOR166C | 33.7 (25) | 28.4 (25) | 6.3E-02 | 0 (0) | 0 (0) | 0.0E+00 | 33.7 (25) | 28.4 (25) | 6.3E-02 |
| YOR170W | 23.3 (10) | 29.3 (10) | 2.1E-01 | 0 (0) | 0 (0) | 0.0E+00 | 23.3 (10) | 29.3 (10) | 2.1E-01 |
| YOR175C | 22.9 (10) | 29.3 (10) | 9.6E-02 | 0 (0) | 0 (0) | 0.0E+00 | 22.9 (10) | 29.3 (10) | 9.6E-02 |

**References:**

1. Kaeberlein M, Powers RW, 3rd, Steffen KK, Westman EA, Hu D, et al. (2005) Regulation of yeast replicative life span by TOR and Sch9 in response to nutrients. Science 310: 1193-1196.
2. Kaeberlein M, Kennedy BK (2005) Large-scale identification in yeast of conserved ageing genes. Mech Ageing Dev 126: 17-21.
