# Supplementary material for: Shortest-Path Network Analysis Is a Useful Approach toward Identifying Genetic Determinants of Longevity
Source: PLoS One. 2008 Nov 25;3(11):e3802. doi: 10.1371/journal.pone.0003802 (PMC2583956; doi:10.1371/journal.pone.0003802)
Supplement: Table S6 — Genes contained in both the randomly selected set of 564 single-gene deletion strains and the predicted longevity associated genes in the binding shortest path longevity network. (0.12 MB PDF) [file pone.0003802.s008.pdf]

Table S6. Genes contained in both the randomly selected set of 564 single-gene deletion strains and the predicted longevity associated genes in the binding shortest path longevity network. (**A**) Replicative life span for each haploid deletion (Δ) and experiment matched wild type mother cells are shown as mean replicative life span with number of cells assayed in parentheses. *P*-value is calculated by a Wilcoxon Rank-Sum test. Pooled haploid data refers to pooled *MATα* and *MAT***a** deletion and wild type data. The binding shortest path longevity network is still enriched for both (**B**) long-lived and **(C)** short-lived deletion strains, relative to the set of randomly selected deleltion strains (R564), when these 11 genes are excluded from the analysis**.** The *p*-value category refers to the results of a Fisher's exact test comparing the frequency of (**B**) increased or (**C**) decreased RLS in the R564 set to the Binding SPLN. *Denotes removal of the 11 overlapping ORFs. Statistical analysis with the 11 overlapping ORFs included is shown in Tables 1 and 2.

A

		MATα			***MAT*a**			**Pooled haploid mating types**		
**ORF**	**GENE**	**Δ**	**BY4742**	**p-value**	**Δ**	**BY4741**	**p-value**	**Δ**	**WT Pooled**	**p-value**
YDR101C	ARX1	24.4 (25)	24.5 (35)	7.6E-01	0 (0)	0 (0)	0.0E+00	24.4 (25)	24.5 (35)	7.6E-01
YER177W	BMH1	20.1 (10)	22.7 (10)	6.8E-01	0 (0)	0 (0)	0.0E+00	20.1 (10)	22.7 (10)	6.8E-01
YDR099W	BMH2	26.1 (15)	28.5 (15)	3.0E-01	0 (0)	0 (0)	0.0E+00	26.1 (15)	28.5 (15)	3.0E-01
YHR142W	CHS7	18 (5)	22.4 (5)	4.6E-01	0 (0)	0 (0)	0.0E+00	18 (5)	22.4 (5)	4.6E-01
YOR144C	ELG1	25 (5)	32.8 (5)	3.8E-01	0 (0)	0 (0)	0.0E+00	25 (5)	32.8 (5)	3.8E-01
YDR283C	GCN2	25.7 (495)	27 (565)	2.8E-02	23.3 (180)	28.8 (220)	1.1E-08	25.1 (675)	27.5 (785)	8.5E-07
YKR048C	NAP1	24.6 (45)	27.2 (45)	6.3E-02	0 (0)	0 (0)	0.0E+00	24.6 (45)	27.2 (45)	6.3E-02
YLR350W	ORM2	22.6 (10)	22.7 (10)	7.0E-01	0 (0)	0 (0)	0.0E+00	22.6 (10)	22.7 (10)	7.0E-01
YGR087C	PDC6	24.5 (25)	24.2 (45)	7.6E-01	0 (0)	0 (0)	0.0E+00	24.5 (25)	24.2 (45)	7.6E-01
YDR129C	SAC6	14.7 (30)	25.8 (60)	2.4E-05	0 (0)	0 (0)	0.0E+00	14.7 (30)	25.8 (60)	2.4E-05
YDR477W	SNF1	38.8 (40)	25.5 (40)	2.4E-06	26.1 (100)	27 (120)	3.5E-01	29.8 (140)	26.6 (160)	4.9E-02

B

Dataset	Both Haploid Mating Types	*p* - value	Pooled < 0.05	*p* - value
Binding SPLN* (77)	9.1 % (7)	-	16.9% (13)	-
R564* (553)	2.7% (15)	0.01	4.2% (23)	1.2×10^{-4}

C

Dataset	Mean RLS < 15	*p* - value	Mean RLS < 20	*p* - value
Binding SPLN* (77)	16.9 % (13)	-	41.6% (32)	-
R564* (553)	3.4% (19)	2.6×10^{-5}	17.4% (96)	4.0×10^{-6}
