# Supplementary material for: Shortest-Path Network Analysis Is a Useful Approach toward Identifying Genetic Determinants of Longevity
Source: PLoS One. 2008 Nov 25;3(11):e3802. doi: 10.1371/journal.pone.0003802 (PMC2583956; doi:10.1371/journal.pone.0003802)
Supplement: Table S7 — Enrichment of longevity-associated genes in the Composite shortest-path longevity network. (0.08 MB PDF) [file pone.0003802.s009.pdf]

Table S7. Enrichment of longevity-associated genes in the Composite shortest-path longevity network. (A) The Composite shortest path longevity network (SPLN) is enriched for single genes deletion mutants with increased replicative life span (RLS), relative to the entire yeast ORF deletion collection (DELSET), based on ongoing RLS analysis for both sets. The percentage of single-gene deletion strains that are significantly long-lived ($p < 0.05$, Wilcoxon Rank-Sum test) in the Composite SPLN or the DELSET is shown. Number of strains in each category shown in parentheses. The p-value category refers to the results of a Fisher's Exact test comparing the frequency of increased RLS in the DELSET, relative to the Composite SPLN. **(B)** The Composite SPLN is enriched for single genes deletion mutants with decreased RLS, relative to the DELSET, based on ongoing RLS analysis for both sets. The percentage of single-gene deletion strains with an observed mean RLS (MRLS) less than 20 or 15 in the Composite SPLN or the DELSET is shown. Number of strains in each category shown in parentheses. The *p*-value category refers to the results of a Fisher's Exact test comparing the frequency of short RLS in the DELSET, relative to the Binding SPLN.

A

Dataset	Both Haploid Mating Types	*p* - value	Pooled < 0.05	*p* - value
Composite SPLN (128)	6.3 % (8)	-	11.7% (15)	-
DELSET (4681)	1.5% (72)	1.7×10^{-3}	6.1% (288)	0.01

B

Dataset	Mean RLS < 15	*p* - value	Mean RLS < 20	*p* - value
Composite SPLN (128)	13.2 % (17)	-	36.7% (47)	-
DELSET (4681)	1.6% (77)	2.6×10^{-4}	17.5% (818)	2.7×10^{-7}
