# Supplementary material for: Shortest-Path Network Analysis Is a Useful Approach toward Identifying Genetic Determinants of Longevity
Source: PLoS One. 2008 Nov 25;3(11):e3802. doi: 10.1371/journal.pone.0003802 (PMC2583956; doi:10.1371/journal.pone.0003802)
Supplement: Table S8 — The first neighbors longevity network has less predictive power than the Binding SPLN. (0.17 MB PDF) [file pone.0003802.s010.pdf]

Table S8. The first neighbors longevity network has less predictive power than the Binding SPLN. (A) The first neighbors longevity network consists of 1375 gene/proteins, of which RLS data is has been obtained for 937 corresponding single-gene deletion mutants. The percentage of single-gene deletion strains that are long-lived in both mating types, long-lived when data from both mating types are pooled, or short-lived (mean life span < 15 or < 20 generations) is shown for the first neighbors longevity network and compared to the current dataset for the entire deletion collection (DELSET). The number of strains in each category is shown in parentheses. The *p*-value category refers to the results of a 2-tailed Fisher's Exact test comparing the DELSET to the First Neighbors in each category. **(B)** The components of the first neighbors longevity network.

A.

Dataset	DELSET (4681)	First Neighbors (968)	p-value
Long-lived both haploid mating types	1.5% (72)	1.9% (18)	0.48
Long-lived pooled	6.1% (288)	6.6% (62)	0.55
Short-lived < 15	5% (231)	8.2% (76)	2.0×10^{-4}
Short-lived < 20	17.5% (818)	23.6% (221)	1.8×10^{-5}

B.

ORF	Gene	ORF	Gene	ORF	Gene
YAL003W	EFB1	YBR039W	ATP3	YBR275C	RIF1
YAL005C	SSA1	YBR040W	FIG1	YBR039W	ATP3
YAL013W	DEP1	YBR052C	RFS1	YBR040W	FIG1
YAL016W	TPD3	YBR060C	ORC2	YBR052C	RFS1
YAL021C	CCR4	YBR083W	TEC1	YBR060C	ORC2
YAL024C	LTE1	YBR084W	MIS1	YBR083W	TEC1
YAL026C	DRS2	YBR085W	AAC3	YBR084W	MIS1
YAL028W	FRT2	YBR087W	RFC5	YBR085W	AAC3
YAL030W	SNC1	YBR093C	PHO5	YBR087W	RFC5
YAL038W	CDC19	YBR095C	RXT2	YBR093C	PHO5
YAL040C	CLN3	YBR103W	SIF2	YBR095C	RXT2

YAL055W	PEX22	YBR112C	CYC8	YBR103W	SIF2
YAL056W	GPB2	YBR122C	MRPL36	YBR112C	CYC8
YAR010C		YBR125C	PTC4	YBR122C	MRPL36
YAR014C	BUD14	YBR126C	TPS1	YBR125C	PTC4
YAR019C	CDC15	YBR128C	ATG14	YBR126C	TPS1
YBL003C	HTA2	YBR130C	SHE3	YBR128C	ATG14
YBL004W	UTP20	YBR133C	HSL7	YBR130C	SHE3
YBL007C	SLA1	YBR135W	CKS1	YBR133C	HSL7
YBL008W	HIR1	YBR136W	MEC1	YBR135W	CKS1
YBL014C	RRN6	YBR139W		YBR136W	MEC1
YBL015W	ACH1	YBR140C	IRA1	YBR139W	
YBL016W	FUS3	YBR142W	MAK5	YBR140C	IRA1
YBL022C	PIM1	YBR143C	SUP45	YBR142W	MAK5
YBL032W	HEK2	YBR150C	TBS1	YBR143C	SUP45
YBL034C	STU1	YBR154C	RPB5	YBR150C	TBS1
YBL038W	MRPL16	YBR160W	CDC28	YBR154C	RPB5
YBL046W	PSY4	YBR164C	ARL1	YBR160W	CDC28
YBL047C	EDE1	YBR169C	SSE2	YBR164C	ARL1
YBL051C	PIN4	YBR183W	YPC1	YBR169C	SSE2
YBL061C	SKT5	YBR184W		YBR183W	YPC1
YBL075C	SSA3	YBR193C	MED8	YBR184W	
YBL076C	ILS1	YBR195C	MSI1	YBR193C	MED8
YBL084C	CDC27	YBR198C	TAF5	YBR195C	MSI1
YBL085W	BOI1	YBR202W	MCM7	YBR198C	TAF5
YBL098W	BNA4	YBR205W	KTR3	YBR202W	MCM7
YBL100WC		YBR218C	PYC2	YBR205W	KTR3
YBL103C	RTG3	YBR225W		YBR218C	PYC2
YBL104C		YBR227C	MCX1	YBR225W	
YBL105C	PKC1	YBR231C	SWC5	YBR227C	MCX1
YBL108W		YBR238C		YBR231C	SWC5
YBR009C	HHF1	YBR245C	ISW1	YBR238C	
YBR010W	HHT1	YBR247C	ENP1	YBR245C	ISW1
YBR011C	IPP1	YBR248C	HIS7	YBR247C	ENP1
YBR018C	GAL7	YBR249C	ARO4	YBR248C	HIS7
YBR019C	GAL10	YBR252W	DUT1	YBR249C	ARO4
YBR023C	CHS3	YBR255W		YBR252W	DUT1
YBR025C	OLA1	YBR264C	YPT10	YBR255W	
YBR028C		YBR266C	SLM6	YBR264C	YPT10
YBR029C	CDS1	YBR267W	REI1	YBR266C	SLM6
YBR031W	RPL4A	YBR268W	MRPL37	YBR267W	REI1
YBR036C	CSG2	YBR274W	CHK1	YBR268W	MRPL37
YBR274W	CHK1	YDL058W	USO1	YDR054C	CDC34
YBR275C	RIF1	YDL059C	RAD59	YDR060W	MAK21
YBR276C	PPS1	YDL060W	TSR1	YDR061W	
YBR279W	PAF1	YDL065C	PEX19	YDR074W	TPS2
YBR281C	DUG2	YDL074C	BRE1	YDR075W	PPH3
YBR287W		YDL076C	RXT3	YDR076W	RAD55
YBR289W	SNF5	YDL085W	NDE2	YDR085C	AFR1
YBR290W	BSD2	YDL097C	RPN6	YDR088C	SLU7
YCL009C	ILV6	YDL100C	GET3	YDR091C	RLI1
YCL016C	DCC1	YDL108W	KIN28	YDR096W	GIS1
YCL025C	AGP1	YDL110C	TMA17	YDR099W	BMH2
YCL029C	BIK1	YDL112W	TRM3	YDR101C	ARX1
YCL030C	HIS4	YDL117W	CYK3	YDR103W	STE5

YCL035C	GRX1	YDL120W	YFH1	YDR108W	GSG1
YCL040W	GLK1	YDL124W		YDR110W	FOB1
YCL046W		YDL126C	CDC48	YDR115W	
YCL061C	MRC1	YDL127W	PCL2	YDR126W	SWF1
YCL065W		YDL129W		YDR129C	SAC6
YCL076W		YDL132W	CDC53	YDR137W	RGP1
YCR005C	CIT2	YDL134C	PPH21	YDR138W	HPR1
YCR009C	RVS161	YDL137W	ARF2	YDR141C	DOP1
YCR018C	SRD1	YDL140C	RPO21	YDR143C	SAN1
YCR018CA		YDL147W	RPN5	YDR146C	SWI5
YCR019W	MAK32	YDL150W	RPC53	YDR148C	KGD2
YCR030C	SYP1	YDL153C	SAS10	YDR162C	NBP2
YCR033W	SNT1	YDL155W	CLB3	YDR168W	CDC37
YCR036W	RBK1	YDL160C	DHH1	YDR170WA	
YCR038C	BUD5	YDL166C	FAP7	YDR172W	SUP35
YCR039C	MATALPHA2	YDL185W	TFP1	YDR181C	SAS4
YCR040W	MATALPHA1	YDL188C	PPH22	YDR188W	CCT6
YCR041W		YDL191W	RPL35A	YDR190C	RVB1
YCR046C	IMG1	YDL192W	ARF1	YDR195W	REF2
YCR053W	THR4	YDL195W	SEC31	YDR196C	
YCR057C	PWP2	YDL203C		YDR200C	VPS64
YCR061W		YDL213C	NOP6	YDR201W	SPC19
YCR066W	RAD18	YDL214C	PRR2	YDR207C	UME6
YCR071C	IMG2	YDL220C	CDC13	YDR208W	MSS4
YCR084C	TUP1	YDL226C	GCS1	YDR212W	TCP1
YCR093W	CDC39	YDL240W	LRG1	YDR214W	AHA1
YCR096C	HMRA2	YDR001C	NTH1	YDR216W	ADR1
YCR098C	GIT1	YDR004W	RAD57	YDR217C	RAD9
YCR105W	ADH7	YDR006C	SOK1	YDR224C	HTB1
YDL002C	NHP10	YDR007W	TRP1	YDR226W	ADK1
YDL003W	MCD1	YDR009W	GAL3	YDR227W	SIR4
YDL007W	RPT2	YDR017C	KCS1	YDR233C	RTN1
YDL022W	GPD1	YDR023W	SES1	YDR235W	PRP42
YDL029W	ARP2	YDR026C		YDR237W	MRPL7
YDL035C	GPR1	YDR027C	VPS54	YDR257C	SET7
YDL040C	NAT1	YDR028C	REG1	YDR259C	YAP6
YDL042C	SIR2	YDR032C	PST2	YDR264C	AKR1
YDL047W	SIT4	YDR037W	KRS1	YDR265W	PEX10
YDL051W	LHP1	YDR040C	ENA1	YDR285W	ZIP1
YDL056W	MBP1	YDR050C	TPI1	YDR287W	INM2
YDR288W	NSE3	YDR510W	SMT3	YER093C	TSC11
YDR292C	SRP101	YDR516C	EMI2	YER095W	RAD51
YDR293C	SSD1	YDR522C	SPS2	YER099C	PRS2
YDR295C	HDA2	YDR543C		YER103W	SSA4
YDR296W	MHR1	YDR544C		YER109C	FLO8
YDR297W	SUR2	YDR545W	YRF1-1	YER110C	KAP123
YDR298C	ATP5	YEL002C	WBP1	YER111C	SWI4
YDR301W	CFT1	YEL003W	GIM4	YER114C	BOI2
YDR309C	GIC2	YEL009C	GCN4	YER120W	SCS2
YDR310C	SUM1	YEL013W	VAC8	YER123W	YCK3
YDR320C	SWA2	YEL015W	EDC3	YER125W	RSP5
YDR322W	MRPL35	YEL021W	URA3	YER132C	PMD1
YDR328C	SKP1	YEL024W	RIP1	YER133W	GLC7
YDR331W	GPI8	YEL025C		YER138C	

YDR334W	SWR1	YEL026W	SNU13	YER146W	LSM5
YDR341C		YEL030W	ECM10	YER148W	SPT15
YDR342C	HXT7	YEL031W	SPF1	YER149C	PEA2
YDR343C	HXT6	YEL032W	MCM3	YER151C	UBP3
YDR353W	TRR1	YEL034W	HYP2	YER154W	OXA1
YDR363W	ESC2	YEL040W	UTR2	YER155C	BEM2
YDR363WA	SEM1	YEL043W		YER160C	
YDR365C	ESF1	YEL044W	IES6	YER169W	RPH1
YDR369C	XRS2	YEL054C	RPL12A	YER172C	BRR2
YDR373W	FRQ1	YEL055C	POL5	YER173W	RAD24
YDR377W	ATP17	YEL056W	HAT2	YER177W	BMH1
YDR380W	ARO10	YEL058W	PCM1	YFL007W	BLM10
YDR381W	YRA1	YEL060C	PRB1	YFL022C	FRS2
YDR385W	EFT2	YEL061C	CIN8	YFL031W	HAC1
YDR388W	RVS167	YER005W	YND1	YFL033C	RIM15
YDR389W	SAC7	YER006W	NUG1	YFL034CB	MOB2
YDR392W	SPT3	YER009W	NTF2	YFL037W	TUB2
YDR394W	RPT3	YER016W	BIM1	YFL039C	ACT1
YDR395W	SXM1	YER020W	GPA2	YFL042C	
YDR398W	UTP5	YER021W	RPN3	YFL045C	SEC53
YDR405W	MRP20	YER022W	SRB4	YFR001W	LOC1
YDR410C	STE14	YER023W	PRO3	YFR004W	RPN11
YDR412W	RRP17	YER025W	GCD11	YFR005C	SAD1
YDR415C		YER027C	GAL83	YFR009W	GCN20
YDR418W	RPL12B	YER033C	ZRG8	YFR010W	UBP6
YDR419W	RAD30	YER040W	GLN3	YFR013W	IOC3
YDR427W	RPN9	YER042W	MXR1	YFR017C	
YDR428C	BNA7	YER043C	SAH1	YFR019W	FAB1
YDR436W	PPZ2	YER050C	RSM18	YFR021W	ATG18
YDR440W	DOT1	YER052C	HOM3	YFR028C	CDC14
YDR446W	ECM11	YER057C	HMF1	YFR037C	RSC8
YDR448W	ADA2	YER062C	HOR2	YFR040W	SAP155
YDR460W	TFB3	YER069W	ARG5,6	YFR044C	DUG1
YDR462W	MRPL28	YER070W	RNR1	YFR047C	BNA6
YDR485C	VPS72	YER082C	UTP7	YFR051C	RET2
YDR490C	PKH1	YER083C	GET2	YFR052W	RPN12
YDR496C	PUF6	YER088C	DOT6	YFR053C	HXK1
YDR505C	PSP1	YER090W	TRP2	YGL001C	ERG26
YDR507C	GIN4	YER091C	MET6	YGL004C	RPN14
YGL008C	PMA1	YGL241W	KAP114	YGR232W	NAS6
YGL009C	LEU1	YGL244W	RTF1	YGR233C	PHO81
YGL014W	PUF4	YGL245W	GUS1	YGR234W	YHB1
YGL016W	KAP122	YGL246C	RAI1	YGR239C	PEX21
YGL019W	CKB1	YGL248W	PDE1	YGR240C	PFK1
YGL020C	GET1	YGL251C	HFM1	YGR244C	LSC2
YGL023C	PIB2	YGL252C	RTG2	YGR245C	SDA1
YGL025C	PGD1	YGL255W	ZRT1	YGR248W	SOL4
YGL026C	TRP5	YGR006W	PRP18	YGR249W	MGA1
YGL027C	CWH41	YGR017W		YGR252W	GCN5
YGL035C	MIG1	YGR023W	MTL1	YGR255C	COQ6
YGL037C	PNC1	YGR040W	KSS1	YGR256W	GND2
YGL045W	RIM8	YGR060W	ERG25	YGR260W	TNA1
YGL048C	RPT6	YGR061C	ADE6	YGR262C	BUD32
YGL049C	TIF4632	YGR063C	SPT4	YGR267C	FOL2

YGL054C	ERV14	YGR066C		YGR282C	BGL2
YGL058W	RAD6	YGR070W	ROM1	YHL003C	LAG1
YGL064C	MRH4	YGR076C	MRPL25	YHL007C	STE20
YGL068W	MNP1	YGR078C	PAC10	YHL011C	PRS3
YGL071W	AFT1	YGR083C	GCD2	YHL030W	ECM29
YGL073W	HSF1	YGR086C	PIL1	YHL034C	SBP1
YGL080W		YGR087C	PDC6	YHL048W	COS8
YGL084C	GUP1	YGR090W	UTP22	YHR001WA	QCR10
YGL086W	MAD1	YGR094W	VAS1	YHR005C	GPA1
YGL099W	LSG1	YGR100W	MDR1	YHR008C	SOD2
YGL111W	NSA1	YGR104C	SRB5	YHR013C	ARD1
YGL115W	SNF4	YGR108W	CLB1	YHR014W	SPO13
YGL116W	CDC20	YGR111W		YHR016C	YSC84
YGL120C	PRP43	YGR113W	DAM1	YHR020W	
YGL121C	GPG1	YGR116W	SPT6	YHR023W	MYO1
YGL127C	SOH1	YGR121C	MEP1	YHR031C	RRM3
YGL130W	CEG1	YGR122W		YHR033W	
YGL131C	SNT2	YGR130C		YHR039C	MSC7
YGL137W	SEC27	YGR132C	PHB1	YHR041C	SRB2
YGL153W	PEX14	YGR133W	PEX4	YHR042W	NCP1
YGL158W	RCK1	YGR150C		YHR052W	CIC1
YGL163C	RAD54	YGR152C	RSR1	YHR053C	CUP1-1
YGL175C	SAE2	YGR154C	GTO1	YHR061C	GIC1
YGL178W	MPT5	YGR155W	CYS4	YHR064C	SSZ1
YGL179C	TOS3	YGR159C	NSR1	YHR074W	QNS1
YGL190C	CDC55	YGR161C	RTS3	YHR084W	STE12
YGL191W	COX13	YGR162W	TIF4631	YHR091C	MSR1
YGL193C		YGR170W	PSD2	YHR098C	SFB3
YGL194C	HOS2	YGR183C	QCR9	YHR099W	TRA1
YGL195W	GCN1	YGR192C	TDH3	YHR102W	KIC1
YGL197W	MDS3	YGR194C	XKS1	YHR104W	GRE3
YGL201C	MCM6	YGR196C	FYV8	YHR106W	TRR2
YGL207W	SPT16	YGR200C	ELP2	YHR110W	ERP5
YGL208W	SIP2	YGR202C	PCT1	YHR112C	
YGL234W	ADE5,7	YGR214W	RPS0A	YHR114W	BZZ1
YGL236C	MTO1	YGR220C	MRPL9	YHR115C	DMA1
YGL238W	CSE1	YGR229C	SMI1	YHR118C	ORC6
YGL240W	DOC1	YGR231C	PHB2	YHR119W	SET1
YHR128W	FUR1	YIL142W	CCT2	YJR007W	SUI2
YHR129C	ARP1	YIL143C	SSL2	YJR010CA	SPC1
YHR135C	YCK1	YIL147C	SLN1	YJR016C	ILV3
YHR142W	CHS7	YIL150C	MCM10	YJR017C	ESS1
YHR149C	SKG6	YIL153W	RRD1	YJR025C	BNA1
YHR152W	SPO12	YIL156W	UBP7	YJR027W	
YHR158C	KEL1	YIL159W	BNR1	YJR028W	
YHR165C	PRP8	YIR002C	MPH1	YJR032W	CPR7
YHR166C	CDC23	YIR019C	MUC1	YJR035W	RAD26
YHR169W	DBP8	YIR023W	DAL81	YJR042W	NUP85
YHR170W	NMD3	YIR038C	GTT1	YJR043C	POL32
YHR174W	ENO2	YJL003W	COX16	YJR046W	TAH11
YHR179W	OYE2	YJL005W	CYR1	YJR048W	CYC1
YHR183W	GND1	YJL008C	CCT8	YJR049C	UTR1
YHR186C	KOG1	YJL014W	CCT3	YJR053W	BFA1
YHR187W	IKI1	YJL015C		YJR057W	CDC8

YHR191C	CTF8	YJL016W		YJR065C	ARP3
YHR193C	EGD2	YJL020C	BBC1	YJR066W	TOR1
YHR194W	MDM31	YJL023C	PET130	YJR067C	YAE1
YHR200W	RPN10	YJL026W	RNR2	YJR068W	RFC2
YHR205W	SCH9	YJL030W	MAD2	YJR072C	NPA3
YHR206W	SKN7	YJL033W	HCA4	YJR073C	OPI3
YIL002C	INP51	YJL052W	TDH1	YJR075W	HOC1
YIL004C	BET1	YJL063C	MRPL8	YJR076C	CDC11
YIL020C	HIS6	YJL076W	NET1	YJR077C	MIR1
YIL026C	IRR1	YJL080C	SCP160	YJR078W	BNA2
YIL028W		YJL089W	SIP4	YJR079W	
YIL033C	BCY1	YJL092W	SRS2	YJR083C	ACF4
YIL035C	CKA1	YJL095W	BCK1	YJR090C	GRR1
YIL037C	PRM2	YJL098W	SAP185	YJR091C	JSN1
YIL038C	NOT3	YJL106W	IME2	YJR094C	IME1
YIL043C	CBR1	YJL109C	UTP10	YJR104C	SOD1
YIL049W	DFG10	YJL110C	GZF3	YJR109C	CPA2
YIL051C	MMF1	YJL114W		YJR112W	NNF1
YIL053W	RHR2	YJL115W	ASF1	YJR117W	STE24
YIL061C	SNP1	YJL116C	NCA3	YJR118C	ILM1
YIL069C	RPS24B	YJL122W	ALB1	YJR130C	STR2
YIL075C	RPN2	YJL123C		YJR134C	SGM1
YIL076W	SEC28	YJL125C	GCD14	YJR135C	MCM22
YIL078W	THS1	YJL128C	PBS2	YJR138W	IML1
YIL084C	SDS3	YJL129C	TRK1	YJR140C	HIR3
YIL094C	LYS12	YJL130C	URA2	YJR144W	MGM101
YIL095W	PRK1	YJL138C	TIF2	YJR145C	RPS4A
YIL106W	MOB1	YJL140W	RPB4	YJR152W	DAL5
YIL112W	HOS4	YJL141C	YAK1	YKL003C	MRP17
YIL119C	RPI1	YJL143W	TIM17	YKL008C	LAC1
YIL123W	SIM1	YJL153C	INO1	YKL010C	UFD4
YIL125W	KGD1	YJL164C	TPK1	YKL014C	URB1
YIL126W	STH1	YJL168C	SET2	YKL015W	PUT3
YIL128W	MET18	YJL176C	SWI3	YKL016C	ATP7
YIL129C	TAO3	YJL187C	SWE1	YKL038W	RGT1
YIL131C	FKH1	YJL197W	UBP12	YKL043W	PHD1
YIL136W	OM45	YJL207C	LAA1	YKL048C	ELM1
YKL049C	CSE4	YKR039W	GAP1	YLR178C	TFS1
YKL052C	ASK1	YKR042W	UTH1	YLR180W	SAM1
YKL056C	TMA19	YKR043C		YLR182W	SWI6
YKL059C	MPE1	YKR048C	NAP1	YLR189C	ATG26
YKL060C	FBA1	YKR056W	TRM2	YLR191W	PEX13
YKL062W	MSN4	YKR067W	GPT2	YLR197W	SIK1
YKL063C		YKR085C	MRPL20	YLR200W	YKE2
YKL067W	YNK1	YKR096W		YLR210W	CLB4
YKL071W		YKR101W	SIR1	YLR215C	CDC123
YKL073W	LHS1	YLL013C	PUF3	YLR216C	CPR6
YKL080W	VMA5	YLL016W		YLR223C	IFH1
YKL081W	TEF4	YLL021W	SPA2	YLR229C	CDC42
YKL085W	MDH1	YLL024C	SSA2	YLR231C	BNA5
YKL087C	CYT2	YLL026W	HSP104	YLR233C	EST1
YKL093W	MBR1	YLL028W	TPO1	YLR238W	FAR10
YKL095W	YJU2	YLL031C	GPI13	YLR246W	ERF2
YKL101W	HSL1	YLL033W	IRC19	YLR249W	YEF3

YKL104C	GFA1	YLL039C	UBI4	YLR258W	GSY2
YKL109W	HAP4	YLL040C	VPS13	YLR259C	HSP60
YKL112W	ABF1	YLL050C	COF1	YLR262C	YPT6
YKL113C	RAD27	YLL060C	GTT2	YLR265C	NEJ1
YKL119C	VPH2	YLR006C	SSK1	YLR274W	MCM5
YKL127W	PGM1	YLR022C	SDO1	YLR278C	
YKL134C	1-Oct	YLR027C	AAT2	YLR285W	NNT1
YKL135C	APL2	YLR039C	RIC1	YLR286C	CTS1
YKL139W	CTK1	YLR044C	PDC1	YLR287CA	RPS30A
YKL140W	TGL1	YLR048W	RPS0B	YLR291C	GCD7
YKL143W	LTV1	YLR053C		YLR292C	SEC72
YKL145W	RPT1	YLR055C	SPT8	YLR294C	
YKL149C	DBR1	YLR059C	REX2	YLR296W	
YKL150W	MCR1	YLR074C	BUD20	YLR303W	MET17
YKL152C	GPM1	YLR079W	SIC1	YLR304C	ACO1
YKL155C	RSM22	YLR080W	EMP46	YLR306W	UBC12
YKL165C	MCD4	YLR085C	ARP6	YLR310C	CDC25
YKL166C	TPK3	YLR086W	SMC4	YLR313C	SPH1
YKL167C	MRP49	YLR095C	IOC2	YLR320W	MMS22
YKL180W	RPL17A	YLR103C	CDC45	YLR324W	PEX30
YKL182W	FAS1	YLR106C	MDN1	YLR332W	MID2
YKL185W	ASH1	YLR109W	AHP1	YLR342W	FKS1
YKL189W	HYM1	YLR113W	HOG1	YLR347C	KAP95
YKL190W	CNB1	YLR116W	MSL5	YLR350W	ORM2
YKL193C	SDS22	YLR117C	CLF1	YLR353W	BUD8
YKL195W	MIA40	YLR120C	YPS1	YLR355C	ILV5
YKL203C	TOR2	YLR131C	ACE2	YLR362W	STE11
YKL204W	EAP1	YLR133W	CKI1	YLR370C	ARC18
YKL210W	UBA1	YLR134W	PDC5	YLR371W	ROM2
YKL217W	JEN1	YLR135W	SLX4	YLR372W	SUR4
YKL218C	SRY1	YLR143W		YLR384C	IKI3
YKR006C	MRPL13	YLR169W		YLR386W	VAC14
YKR008W	RSC4	YLR171W		YLR387C	REH1
YKR026C	GCN3	YLR174W	IDP2	YLR396C	VPS33
YKR029C	SET3	YLR175W	CBF5	YLR398C	SKI2
YKR034W	DAL80	YLR177W		YLR403W	SFP1
YLR406C	RPL31B	YMR012W	CLU1	YMR238W	DFG5
YLR410W	VIP1	YMR016C	SOK2	YMR242C	RPL20A
YLR417W	VPS36	YMR026C	PEX12	YMR246W	FAA4
YLR418C	CDC73	YMR028W	TAP42	YMR250W	GAD1
YLR421C	RPN13	YMR029C	FAR8	YMR255W	GFD1
YLR425W	TUS1	YMR037C	MSN2	YMR256C	COX7
YLR429W	CRN1	YMR039C	SUB1	YMR260C	TIF11
YLR438W	CAR2	YMR042W	ARG80	YMR261C	TPS3
YLR440C	SEC39	YMR047C	NUP116	YMR263W	SAP30
YLR442C	SIR3	YMR048W	CSM3	YMR266W	RSN1
YLR447C	VMA6	YMR049C	ERB1	YMR267W	PPA2
YLR448W	RPL6B	YMR052W	FAR3	YMR268C	PRP24
YLR452C	SST2	YMR059W	SEN15	YMR270C	RRN9
YLR453C	RIF2	YMR061W	RNA14	YMR272C	SCS7
YLR463C		YMR068W	AVO2	YMR273C	ZDS1
YLR465C	BSC3	YMR072W	ABF2	YMR274C	RCE1
YLR467W	YRF1-5	YMR074C		YMR277W	FCP1
YML006C	GIS4	YMR075W	RCO1	YMR280C	CAT8

YML007W	YAP1	YMR077C	VPS20	YMR284W	YKU70
YML008C	ERG6	YMR078C	CTF18	YMR285C	NGL2
YML009C	MRPL39	YMR081C	ISF1	YMR290C	HAS1
YML010W	SPT5	YMR086W		YMR291W	
YML011C	RAD33	YMR092C	AIP1	YMR293C	
YML017W	PSP2	YMR097C	MTG1	YMR294W	JNM1
YML025C	YML6	YMR105C	PGM2	YMR298W	LIP1
YML028W	TSA1	YMR106C	YKU80	YMR303C	ADH2
YML032C	RAD52	YMR109W	MYO5	YMR304W	UBP15
YML041C	VPS71	YMR125W	STO1	YMR307W	GAS1
YML046W	PRP39	YMR127C	SAS2	YMR312W	ELP6
YML048W	GSF2	YMR128W	ECM16	YMR319C	FET4
YML049C	RSE1	YMR139W	RIM11	YNL002C	RLP7
YML054C	CYB2	YMR144W		YNL004W	HRB1
YML057W	CMP2	YMR149W	SWP1	YNL005C	MRP7
YML059C	NTE1	YMR153W	NUP53	YNL006W	LST8
YML064C	TEM1	YMR154C	RIM13	YNL007C	SIS1
YML065W	ORC1	YMR163C	INP2	YNL009W	IDP3
YML069W	POB3	YMR164C	MSS11	YNL014W	HEF3
YML073C	RPL6A	YMR172W	HOT1	YNL021W	HDA1
YML074C	FPR3	YMR175W	SIP18	YNL025C	SSN8
YML078W	CPR3	YMR182C	RGM1	YNL027W	CRZ1
YML085C	TUB1	YMR186W	HSC82	YNL030W	HHF2
YML087C		YMR190C	SGS1	YNL037C	IDH1
YML089C		YMR193W	MRPL24	YNL038W	GPI15
YML091C	RPM2	YMR194W	RPL36A	YNL055C	POR1
YML094W	GIM5	YMR198W	CIK1	YNL061W	NOP2
YML098W	TAF13	YMR200W	ROT1	YNL063W	MTQ1
YML099C	ARG81	YMR219W	ESC1	YNL064C	YDJ1
YML100W	TSL1	YMR224C	MRE11	YNL066W	SUN4
YML109W	ZDS2	YMR225C	MRPL44	YNL073W	MSK1
YML110C	COQ5	YMR226C		YNL076W	MKS1
YML126C	ERG13	YMR227C	TAF7	YNL079C	TPM1
YML128C	MSC1	YMR228W	MTF1	YNL085W	MKT1
YMR011W	HXT2	YMR229C	RRP5	YNL088W	TOP2
YNL090W	RHO2	YNL271C	BNI1	YOL110W	SHR5
YNL093W	YPT53	YNL273W	TOF1	YOL115W	PAP2
YNL097C	PHO23	YNL280C	ERG24	YOL121C	RPS19A
YNL098C	RAS2	YNL283C	WSC2	YOL130W	ALR1
YNL101W	AVT4	YNL284C	MRPL10	YOL139C	CDC33
YNL102W	POL1	YNL289W	PCL1	YOL148C	SPT20
YNL107W	YAF9	YNL290W	RFC3	YOL149W	DCP1
YNL110C	NOP15	YNL298W	CLA4	YOL151W	GRE2
YNL113W	RPC19	YNL302C	RPS19B	YOL156W	HXT11
YNL116W	DMA2	YNL304W	YPT11	YOR007C	SGT2
YNL118C	DCP2	YNL307C	MCK1	YOR008C	SLG1
YNL123W	NMA111	YNL308C	KRI1	YOR014W	RTS1
YNL124W	NAF1	YNL309W	STB1	YOR018W	ROD1
YNL127W	FAR11	YNL314W	DAL82	YOR026W	BUB3
YNL129W	NRK1	YNL327W	EGT2	YOR027W	STI1
YNL131W	TOM22	YNL329C	PEX6	YOR028C	CIN5
YNL132W	KRE33	YNL334C	SNO2	YOR030W	DFG16
YNL135C	FPR1	YNL337W		YOR032C	HMS1
YNL138W	SRV2	YNL338W		YOR038C	HIR2

YNL139C	THO2
YNL142W	MEP2
YNL145W	MFA2
YNL151C	RPC31
YNL153C	GIM3
YNL154C	YCK2
YNL167C	SKO1
YNL172W	APC1
YNL175C	NOP13
YNL183C	NPR1
YNL186W	UBP10
YNL187W	
YNL189W	SRP1
YNL192W	CHS1
YNL197C	WHI3
YNL199C	GCR2
YNL201C	PSY2
YNL204C	SPS18
YNL206C	RTT106
YNL207W	RIO2
YNL214W	PEX17
YNL216W	RAP1
YNL229C	URE2
YNL233W	BNI4
YNL236W	SIN4
YNL239W	LAP3
YNL248C	RPA49
YNL250W	RAD50
YNL251C	NRD1
YNL252C	MRPL17
YNL259C	ATX1
YNL261W	ORC5
YNL262W	POL2
YNL264C	PDR17
YOR199W	
YOR201C	MRM1
YOR202W	HIS3
YOR207C	RET1
YOR209C	NPT1
YOR210W	RPB10
YOR212W	STE4
YOR213C	SAS5
YOR215C	
YOR217W	RFC1
YOR224C	RPB8
YOR230W	WTM1
YOR232W	MGE1
YOR238W	
YOR239W	ABP140
YOR244W	ESA1
YOR245C	DGA1
YOR249C	APC5
YOR257W	CDC31
YOR259C	RPT4
YNL339C	YRF1-6
YNR001C	CIT1
YNR003C	RPC34
YNR008W	LRO1
YNR016C	ACC1
YNR023W	SNF12
YNR035C	ARC35
YNR037C	RSM19
YNR050C	LYS9
YNR051C	BRE5
YNR052C	POP2
YNR053C	NOG2
YNR065C	
YOL004W	SIN3
YOL006C	TOP1
YOL012C	HTZ1
YOL017W	ESC8
YOL025W	LAG2
YOL026C	MIM1
YOL041C	NOP12
YOL044W	PEX15
YOL051W	GAL11
YOL061W	PRS5
YOL067C	RTG1
YOL068C	HST1
YOL077C	BRX1
YOL078W	AVO1
YOL081W	IRA2
YOL086C	ADH1
YOL094C	RFC4
YOL097C	WRS1
YOL107W	
YOL108C	INO4
YOL109W	ZEO1
YPL042C	SSN3
YPL043W	NOP4
YPL045W	VPS16
YPL046C	ELC1
YPL048W	CAM1
YPL049C	DIG1
YPL055C	LGE1
YPL059W	GRX5
YPL061W	ALD6
YPL066W	
YPL075W	GCR1
YPL076W	GPI2
YPL078C	ATP4
YPL083C	SEN54
YPL085W	SEC16
YPL086C	ELP3
YPL087W	YDC1
YPL093W	NOG1
YPL096CA	ERI1
YPL100W	ATG21
YOR039W	CKB2
YOR041C	
YOR043W	WHI2
YOR046C	DBP5
YOR047C	STD1
YOR052C	
YOR057W	SGT1
YOR061W	CKA2
YOR064C	YNG1
YOR070C	GYP1
YOR074C	CDC21
YOR080W	DIA2
YOR089C	VPS21
YOR092W	ECM3
YOR098C	NUP1
YOR101W	RAS1
YOR107W	RGS2
YOR116C	RPO31
YOR117W	RPT5
YOR122C	PFY1
YOR133W	EFT1
YOR135C	IRC14
YOR136W	IDH2
YOR140W	SFL1
YOR142W	LSC1
YOR144C	ELG1
YOR149C	SMP3
YOR150W	MRPL23
YOR156C	NFI1
YOR174W	MED4
YOR177C	MPC54
YOR181W	LAS17
YOR191W	ULS1
YOR198C	BFR1
YPL256C	CLN2
YPL258C	THI21
YPL259C	APM1
YPL266W	DIM1
YPL268W	PLC1
YPL271W	ATP15
YPR009W	SUT2
YPR010C	RPA135
YPR016C	TIF6
YPR018W	RLF2
YPR019W	MCM4
YPR020W	ATP20
YPR023C	EAF3
YPR025C	CCL1
YPR030W	CSR2
YPR031W	NTO1
YPR033C	HTS1
YPR034W	ARP7
YPR035W	GLN1
YPR046W	MCM16

YOR261C	RPN8	YPL101W	ELP4	YPR067W	ISA2
YOR267C	HRK1	YPL104W	MSD1	YPR069C	SPE3
YOR272W	YTM1	YPL106C	SSE1	YPR072W	NOT5
YOR290C	SNF2	YPL115C	BEM3	YPR080W	TEF1
YOR299W	BUD7	YPL126W	NAN1	YPR100W	MRPL51
YOR304W	ISW2	YPL129W	TAF14	YPR101W	SNT309
YOR310C	NOP58	YPL139C	UME1	YPR104C	FHL1
YOR312C	RPL20B	YPL143W	RPL33A	YPR108W	RPN7
YOR317W	FAA1	YPL151C	PRP46	YPR110C	RPC40
YOR326W	MYO2	YPL152W	RRD2	YPR111W	DBF20
YOR332W	VMA4	YPL153C	RAD53	YPR119W	CLB2
YOR335C	ALA1	YPL160W	CDC60	YPR120C	CLB5
YOR341W	RPA190	YPL161C	BEM4	YPR121W	THI22
YOR349W	CIN1	YPL169C	MEX67	YPR122W	AXL1
YOR358W	HAP5	YPL171C	OYE3	YPR129W	SCD6
YOR360C	PDE2	YPL173W	MRPL40	YPR133WA	TOM5
YOR361C	PRT1	YPL174C	NIP100	YPR135W	CTF4
YOR367W	SCP1	YPL178W	CBC2	YPR138C	MEP3
YOR368W	RAD17	YPL180W	TCO89	YPR141C	KAR3
YOR371C	GPB1	YPL181W	CTI6	YPR143W	RRP15
YOR383C	FIT3	YPL194W	DDC1	YPR160W	GPH1
YPL001W	HAT1	YPL203W	TPK2	YPR161C	SGV1
YPL004C	LSP1	YPL204W	HRR25	YPR163C	TIF3
YPL011C	TAF3	YPL209C	IPL1	YPR164W	MMS1
YPL012W	RRP12	YPL218W	SAR1	YPR165W	RHO1
YPL016W	SWI1	YPL225W		YPR167C	MET16
YPL017C	IRC15	YPL226W	NEW1	YPR169W	JIP5
YPL028W	ERG10	YPL232W	SSO1	YPR179C	HDA3
YPL031C	PHO85	YPL235W	RVB2	YPR181C	SEC23
YPL032C	SVL3	YPL237W	SUI3	YPR183W	DPM1
YPL036W	PMA2	YPL240C	HSP82	YPR187W	RPO26
YPL037C	EGD1	YPL248C	GAL4	YPR190C	RPC82
YPL038W	MET31	YPL252C	YAH1	YPR191W	QCR2
